# Exome sequencing-driven discovery of coding polymorphisms associated with common metabolic phenotypes

**DOI:** 10.1007/s00125-012-2756-1

**Published:** 2012-11-19

**Authors:** A. Albrechtsen, N. Grarup, Y. Li, T. Sparsø, G. Tian, H. Cao, T. Jiang, S. Y. Kim, T. Korneliussen, Q. Li, C. Nie, R. Wu, L. Skotte, A. P. Morris, C. Ladenvall, S. Cauchi, A. Stančáková, G. Andersen, A. Astrup, K. Banasik, A. J. Bennett, L. Bolund, G. Charpentier, Y. Chen, J. M. Dekker, A. S. F. Doney, M. Dorkhan, T. Forsen, T. M. Frayling, C. J. Groves, Y. Gui, G. Hallmans, A. T. Hattersley, K. He, G. A. Hitman, J. Holmkvist, S. Huang, H. Jiang, X. Jin, J. M. Justesen, K. Kristiansen, J. Kuusisto, M. Lajer, O. Lantieri, W. Li, H. Liang, Q. Liao, X. Liu, T. Ma, X. Ma, M. P. Manijak, M. Marre, J. Mokrosiński, A. D. Morris, B. Mu, A. A. Nielsen, G. Nijpels, P. Nilsson, C. N. A. Palmer, N. W. Rayner, F. Renström, R. Ribel-Madsen, N. Robertson, O. Rolandsson, P. Rossing, T. W. Schwartz, P. E. Slagboom, M. Sterner, D.E.S.I.R. Study Group, M. Tang, L. Tarnow, the DIAGRAM Consortium, T. Tuomi, E. van’t Riet, N. van Leeuwen, T. V. Varga, M. A. Vestmar, M. Walker, B. Wang, Y. Wang, H. Wu, F. Xi, L. Yengo, C. Yu, X. Zhang, J. Zhang, Q. Zhang, W. Zhang, H. Zheng, Y. Zhou, D. Altshuler, L. M. ‘t Hart, P. W. Franks, B. Balkau, P. Froguel, M. I. McCarthy, M. Laakso, L. Groop, C. Christensen, I. Brandslund, T. Lauritzen, D. R. Witte, A. Linneberg, T. Jørgensen, T. Hansen, J. Wang, R. Nielsen, O. Pedersen

**Affiliations:** 1 Centre of Bioinformatics, Faculty of Science, University of Copenhagen, Copenhagen, Denmark; 2 The Novo Nordisk Foundation Center for Basic Metabolic Research, Faculty of Health and Medical Sciences, University of Copenhagen, DIKU Building, Universitetsparken 1, 2100 Copenhagen Ø, Denmark; 3 BGI-Shenzhen, Beishan Industrial Zone, Yantian District, 518083 Shenzhen, China; 4 BGI-Tianjin, Tianjin, China; 5 Department of Integrative Biology, University of California, 3060 Valley Life Sciences, Bldg #3140, Berkeley, CA 94720-3140 USA; 6 Wellcome Trust Centre for Human Genetics, University of Oxford, Oxford, UK; 7 Department of Clinical Sciences, Diabetes and Endocrinology, Lund University and Lund University Diabetes Centre, Malmö, Sweden; 8 UMR CNRS 8199, Genomic and Metabolic Disease, Lille, France; 9 Department of Medicine, University of Eastern Finland and Kuopio University Hospital, Kuopio, Finland; 10 Department of Human Nutrition, Faculty of Science, University of Copenhagen, Copenhagen, Denmark; 11 Oxford Centre for Diabetes, Endocrinology and Metabolism, University of Oxford, Oxford, UK; 12 Institute of Human Genetics, Aarhus University, Aarhus, Denmark; 13 Department of Endocrinology-Diabetology, Corbeil-Essonnes Hospital, Corbeil-Essonnes, France; 14 EMGO Institute for Health and Care Research, VU University Medical Center, Amsterdam, the Netherlands; 15 Diabetes Research Centre, Biomedical Research Institute, University of Dundee, Ninewells Hospital, Dundee, UK; 16 Pharmacogenomics Centre, Biomedical Research Institute, University of Dundee, Ninewells Hospital, Dundee, UK; 17 Department of General Practice and Primary Health Care, University of Helsinki, Helsinki, Finland; 18 Vasa Health Care Center, Vaasa, Finland; 19 Genetics of Complex Traits, Institute of Biomedical and Clinical Science, Peninsula Medical School, University of Exeter, Exeter, UK; 20 Diabetes Genetics, Institute of Biomedical and Clinical Science, Peninsula Medical School, University of Exeter, Exeter, UK; 21 Department of Public Health and Clinical Medicine, Umeå University, Umeå, Sweden; 22 Chinese PLA General Hospital, Beijing, China; 23 Centre for Diabetes, Blizard Institute, Queen Mary University of London, London, UK; 24 Vipergen Aps, Copenhagen, Denmark; 25 School of Bioscience and Biotechnology, South China University of Technology, Guangzhou, China; 26 Department of Biology, Faculty of Science, University of Copenhagen, Copenhagen, Denmark; 27 Steno Diabetes Center, Gentofte, Denmark; 28 Institut inter Regional pour la Santé (IRSA), La Riche, France; 29 Department of Endocrinology, Diabetology and Nutrition, Bichat-Claude Bernard University Hospital, Assistance Publique des Hôpitaux de Paris, Paris, France; 30 Inserm U695, Université Denis Diderot Paris 7, Paris, France; 31 Laboratory for Molecular Pharmacology, Department of Pharmacology, Faculty of Health and Medical Sciences, University of Copenhagen, Copenhagen, Denmark; 32 Department of Clinical Biochemistry, Vejle Hospital, Vejle, Denmark; 33 Department of Clinical Sciences, Medicine, Lund University, Malmö, Sweden; 34 Department of Clinical Sciences, Genetic and Molecular Epidemiology Unit, Skåna University Hospital, Lund University, Malmö, Sweden; 35 Section of Molecular Epidemiology, Leiden University Medical Center, Leiden, the Netherlands; 36 Netherlands Center for Healthy Ageing, Leiden, the Netherlands; 37 Department of Medicine, Helsinki University Hospital, Helsinki, Finland; 38 Folkhälsan Research Center, Helsinki, Finland; 39 Department of Molecular Cell Biology, Leiden University Medical Center, Leiden, the Netherlands; 40 Diabetes Research Group, School of Clinical Medical Sciences, Newcastle University, Newcastle upon Tyne, UK; 41 Analytic and Translational Genetics Unit, Massachusetts General Hospital, Boston, MA USA; 42 Broad Institute of Harvard and MIT, Cambridge, MA USA; 43 Department of Nutrition, Harvard School of Public Health, Boston, MA USA; 44 Inserm CESP U1018, Villejuif, France; 45 Genomic Medicine, Hammersmith Hospital, Imperial College London, London, UK; 46 Oxford National Institute for Health Research Biomedical Research Centre, Churchill Hospital, Oxford, UK; 47 Department of Internal Medicine and Endocrinology, Vejle Hospital, Vejle, Denmark; 48 Institute of Regional Health Research, University of Southern Denmark, Odense, Denmark; 49 Department of General Practice, Aarhus University, Aarhus, Denmark; 50 Research Centre for Prevention and Health, Glostrup University Hospital, Glostrup, Denmark; 51 Faculty of Health and Medical Sciences, University of Copenhagen, Copenhagen, Denmark; 52 Faculty of Medicine, University of Aalborg, Aalborg, Denmark; 53 Faculty of Health Sciences, University of Southern Denmark, Odense, Denmark; 54 Department of Statistics, University of California, Berkeley, CA USA; 55 Faculty of Health Sciences, Aarhus University, Aarhus, Denmark; 56 Hagedorn Research Institute, Gentofte, Denmark; 57 Institute of Biomedical Science, Faculty of Health and Medical Sciences, University of Copenhagen, Copenhagen, Denmark

**Keywords:** Exome sequencing, Genetic epidemiology, Genetics, Lipids, Next-generation sequencing, Obesity, Type 2 diabetes

## Abstract

**Aims/hypothesis:**

Human complex metabolic traits are in part regulated by genetic determinants. Here we applied exome sequencing to identify novel associations of coding polymorphisms at minor allele frequencies (MAFs) >1% with common metabolic phenotypes.

**Methods:**

The study comprised three stages. We performed medium-depth (8×) whole exome sequencing in 1,000 cases with type 2 diabetes, BMI >27.5 kg/m^2^ and hypertension and in 1,000 controls (stage 1). We selected 16,192 polymorphisms nominally associated (*p* < 0.05) with case–control status, from four selected annotation categories or from loci reported to associate with metabolic traits. These variants were genotyped in 15,989 Danes to search for association with 12 metabolic phenotypes (stage 2). In stage 3, polymorphisms showing potential associations were genotyped in a further 63,896 Europeans.

**Results:**

Exome sequencing identified 70,182 polymorphisms with MAF >1%. In stage 2 we identified 51 potential associations with one or more of eight metabolic phenotypes covered by 45 unique polymorphisms. In meta-analyses of stage 2 and stage 3 results, we demonstrated robust associations for coding polymorphisms in *CD300LG* (fasting HDL-cholesterol: MAF 3.5%, *p* = 8.5 × 10^−14^), *COBLL1* (type 2 diabetes: MAF 12.5%, OR 0.88, *p* = 1.2 × 10^−11^) and *MACF1* (type 2 diabetes: MAF 23.4%, OR 1.10, *p* = 8.2 × 10^−10^).

**Conclusions/interpretation:**

We applied exome sequencing as a basis for finding genetic determinants of metabolic traits and show the existence of low-frequency and common coding polymorphisms with impact on common metabolic traits. Based on our study, coding polymorphisms with MAF above 1% do not seem to have particularly high effect sizes on the measured metabolic traits.

**Electronic supplementary material:**

The online version of this article (doi:10.1007/s00125-012-2756-1) contains peer-reviewed but unedited supplementary material, which is available to authorised users.

## Introduction

Over the last few years, genome-wide association studies (GWAS) have led to substantial progress in mapping common genetic variation with impact on common phenotypes including those of the metabolic syndrome [1–10]. This advance has revealed hundreds of genetic determinants of human complex phenotypes [1]. Despite this progress a major part of the heritable contribution to variation in most widespread metabolic traits remains unaccounted for [11]. Thus, for type 2 diabetes and related metabolic traits it has been estimated that 10–30% of the observed heritability can be attributed to the hitherto identified variants [2, 4, 8, 10].

DNA sequencing has emerged as a powerful technology enabling detection of low-frequency and rare variation not captured by initial GWAS design and in future studies the GWAS approach may be complemented by imputation of single nucleotide polymorphisms (SNPs) from whole-genome sequencing of a subset of individuals [12]. Sequencing of all genes in the genome (exome) [13, 14] is an alternative approach relying on the hypothesis that functional disease-associated variation resides in the coding regions. Exome sequencing has proven valuable in the search for mutations responsible for Mendelian diseases [15, 16] and emerging reports suggest the benefit of applying large-scale exome sequencing to uncover variation associated with complex human traits [17, 18].

**Fig. 1:**
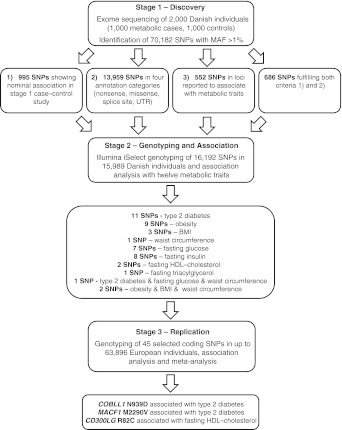
Overview of the study. UTR, untranslated region

Here we present the results of a first-generation medium-pass (8×) exome sequencing approach in 2,000 Danish individuals (stage 1) with follow-up of 16,192 SNPs in 15,989 Danes (stage 2) and replication of 45 SNPs, discovered in a joint analysis of stage 1 and 2, in up to 63,896 Europeans (stage 3) (Fig. 1). To achieve sufficient statistical power a large number of the SNPs selected from stage 1 were genotyped in the much larger sample size in stage 2 making the statistical power comparable to a study where all individuals from both stage 1 and 2 are genotyped for all SNPs [19]. Our objective was to find novel associations of coding variants at minor allele frequencies (MAFs) above 1% with metabolic phenotypes.

## Methods

### Study populations

Danish individuals investigated in stage 1 and 2 of the study were selected from five Danish centres (electronic supplementary material [ESM] Table 1). Exome sequencing in stage 1 (Fig. 1) was performed in 2,000 individuals. Of these, 1,000 were cases recruited based on the presence of type 2 diabetes, BMI >27.5 kg/m^2^ and hypertension (systolic/diastolic BP >140/90 mmHg or use of antihypertensive medication) to represent common forms of type 2 diabetes and 1,000 were control individuals who all had fasting plasma glucose <5.6 mmol/l, 2 h post-OGTT plasma glucose <7.8 mmol/l, BMI <27.5 kg/m^2^ and BP <140/90 mmHg (ESM Table 2). In stage 2, 16,192 SNPs were analysed in all 15,989 Danish individuals recruited from five Danish centres (ESM Table 1) in order to perform association mapping of metabolic traits. The individuals in whom exome sequencing was performed in stage 1 were, to obtain called genotypes, among the 15,989 samples genotyped in stage 2. Data from the five Danish centres were pooled in stage 2 analyses. In brief, type 2 diabetes association studies were performed in 4,854 cases defined by WHO 1999 criteria [20] and in 7,325 non-diabetic control individuals. Obesity was studied in 5,488 obese cases (BMI ≥30 kg/m^2^) and 4,851 lean controls (BMI <25 kg/m^2^) while hypertension was investigated in 7,299 cases (BP >140/90 mmHg or treated with antihypertensive medication) and 3,290 controls (BP <140/90 mmHg). In analysis of quantifiable metabolic traits, BMI and waist circumference were studied in all samples (*n* up to 14,819) with available phenotype data excluding individuals treated with insulin. In studies of fasting plasma glucose (*n* = 9,087) and fasting serum insulin (*n* = 8,419) all previously diagnosed and treated diabetes patients (*n* = 1,743) were excluded while individuals treated with lipid-lowering drugs (*n* = 110) were excluded in analyses of fasting lipid levels (*n* = 13,326). In studies of systolic and diastolic BP all individuals treated with antihypertensive medication (*n* = 968) were excluded leaving 12,651 individuals for analyses. Clinical samples from six different European countries were investigated in replication studies of selected SNPs (stage 3) (ESM Table 3). All participants in the study gave written informed consent. The studies were conducted in accordance with the Declaration of Helsinki II and were approved by the local Ethical Committees.

### Exon capture, Illumina sequencing and quality control of exome sequencing outcome

Exome capture by a NimbleGen 2.1M HD array (target region 34.1 Mb, 21,810 genes) and Illumina GAII sequencing were performed on DNA from the 2,000 individuals by methods previously described [14]. Samples were not randomised in the capture and sequencing processes. The effective reads (ESM Table 4) were aligned to the human reference genome (assembly hg18, NCBI build 36.3) using SOAPaligner (http://soap.genomics.org.cn/, accessed 01/03/2009). The average sequencing depth per sample was 11× and 96% of targeted bases were covered by at least one read (ESM Fig. 1). All uniquely mapped reads were used for further analyses. The nucleotide mismatch rates were estimated from the proportion of mismatches between all bases from uniquely aligned reads. The mismatch rates ranged from 0.41% to 2.65% in the 2,000 samples with a median of 1%. To investigate the error distributions in the data, the average base quality (the Q-score) of all sequencing reads was examined. When increasing the Q-score threshold the average depth declined. The Q20 threshold (1% error rate by quality score definition) was applied in all further analyses. Further quality control was done by investigating the mismatch rates of aligned bases with different quality scores and by examining the distribution of per-base sequencing depth (ESM Fig. 2). Due to the use of the Q20 threshold and multiple hits restrictions approximately 20% of the sequencing data was discarded and the average depth per site declined from 11× to 8× (8.2× in controls, 7.7× in cases, ESM Fig. 3).

The data quality for individual samples was evaluated using called genotypes by comparing with previously genotyped SNPs and by comparing phenotypic sex with genetically determined sex estimated from the heterozygosity of the SNPs on the X-chromosome. Following bar-coding and sex comparison, 1,974 samples (986 cases and 988 controls) were available for SNP detection and association analyses.

### SNP detection and allele frequency estimation in exome sequencing data

Two different approaches to obtain genotype likelihoods were applied. SOAPsnp (http://soap.genomics.org.cn/, accessed 01/03/2009) [21] was used to generate genotype likelihoods, which were used to call genotypes for quality control. For allele frequency estimation and association analysis we estimated the type-specific error rates directly from the putative polymorphic sites and used these error rates combined with the base counts at each position to obtain the genotype likelihoods [20]. As a first step in the identification of SNPs for association testing the allele frequencies of all putative polymorphic sites were estimated using the allele frequency estimator by Li et al [14]. A high error rate of 0.25% was assumed for all error types. Putative polymorphic sites were those with an allele frequency above 0.25%. Then, the allele frequencies were estimated using a maximum likelihood estimator [22], which assumes that the sites are diallelic and takes the uncertainty in the minor allele into account by summing likelihoods over all possible three minor alleles. The discovered SNPs were compared with HapMap data for overlapping SNPs and showed high concordance (ESM Fig. 4). Comparison of allele frequencies with SNPs genotyped in stage 2 (see below) showed high correlation (ESM Fig. 5).

### Association analyses in stage 1

We identified 70,182 variable sites with an allele frequency higher than 1% and a total depth per site summed across all individuals above 1,000× corresponding to 0.5× per individual (Table 1, ESM Table 5). Before performing association analysis on the sequencing data we chose to include multiple stringent filters. This was done to remove SNPs that either were likely to be errors or showed bias that could be correlated with case–control status (ESM Fig. 6). Filters based on the base quality scores and based on biases observed in sequencing time were applied. The case–control association analyses were performed using a likelihood ratio test directly on the observed reads taking the uncertainty of the reads into account [22]. No covariates were included in the analysis.

**Table 1:** Sequencing of 1,974 Danish individuals identified 70,182 SNPs with MAF >1%

SNP annotation	No. of identified SNPs
Nonsense	243
Non-synonymous	20,202
Splice site	301
3′ UTR/5′ UTR	2,756
Synonymous	20,251
Near gene	239
Intron	25,801
Intergenic	389
Total	70,182

The annotation of 70,182 SNPs was performed using the SeattleSNP annotator (http://gvs.gs.washington.edu/SeattleSeqAnnotation/, accessed 01/07/2009) and dbSNP (www.ncbi.nlm.nih.gov/projects/SNP/, accessed 01/07/2009) annotation tools. A proportion of the SNPs were annotated as located in introns or near genes. This is largely due to the fact that sequencing reads sometimes overlap with other parts of the genome in the proximity. SNPs annotated as nonsense, non-synonymous, splice site, and 3′ UTR/5′ UTR were selected for stage 2 genotyping

UTR, untranslated region

Although the filtering removed the bias from the single SNP analysis, burden tests are much more sensitive to small biases because the bias accumulates when analysing multiple variants. Therefore, no burden tests were performed.

### SNP selection for stage 2

SNPs were selected for genotyping in stage 2 from the exome sequencing based on three criteria: (1) SNPs nominally associated (*p* < 0.05) with case–control status in stage 1 were selected; (2) all SNPs annotated to one of the four annotation categories (i.e. all variants annotated as nonsense variants, non-synonymous variants, variants located in splice sites or variants in untranslated regions) were prioritised regardless of association *p* value; (3) synonymous variants in 192 loci previously associated with common metabolic traits at genome-wide significance. After quality control (see below), 16,192 SNPs were available for analyses. Of these, 995 SNPs were selected based on the first criterion, 13,959 based on the second criterion while 686 fulfilled both the first and the second criteria. Finally, 552 SNPs were selected based on the third criterion (Fig. 1).

### Stage 2 genotyping, quality control and association analyses

SNPs selected from stage 1 were genotyped in 16,988 samples in stage 2 by a custom-designed Illumina iSelect array. Samples were randomised before genotyping. Quality control of samples included removing closely related individuals, individuals with an extreme inbreeding coefficient, individuals with a low call rate, individuals with a mislabelled sex and individuals with a high discordance rate to previously genotyped SNPs. The quality control criteria were fulfilled by 15,989 individuals. Genotypes were obtained for 18,744 SNPs. Of the SNPs discovered in stage 1, 5.1% were not polymorphic when genotyped in stage 2. The SNPs were filtered based on their MAF (>0.5%), genotype call rate (>95%), Hardy–Weinberg equilibrium (*p* > 10^−7^) or cross-hybridisation with the X-chromosome, with 16,192 SNPs passing all filters.

Two analyses were done on stage 2 data. First, an enrichment analysis of SNPs selected from stage 1 based on nominal association (*n* = 1,681) was performed estimating the over-representation of low *p* values in analyses of type 2 diabetes, obesity and hypertension in stage 2 data. Second, we performed single SNP association analyses, including stage 2 genotyping data for all 15,989 stage 1 and stage 2 individuals, with 12 variables and SNPs for replication in stage 3 were selected based on these analyses. Three binary (type 2 diabetes, obesity, hypertension) and nine quantitative traits (BMI, waist circumference, systolic and diastolic BP, fasting levels of plasma glucose and serum insulin, cholesterol, HDL-cholesterol and triacylglycerol) were analysed. Association analysis of each SNP was performed using linear or logistic regression assuming an additive or log-additive model. Principal component analysis was performed using the covariance matrix [23] and the first principal component and sex were included in the model as covariates. All quantitative traits were rank normalised to a normal distribution before analysis. No inflations in test statistics for the 12 traits were observed after correction by genomic control (λ_GC_ 1.00–1.09, ESM Fig. 7).

### SNP selection for stage 3

To follow up on the most promising associations from stage 2 association analyses we selected top hits for 12 different metabolic traits. SNPs were selected based on association for each trait (*p* < 10^−3^ for type 2 diabetes, obesity, BMI, waist circumference, fasting glucose, fasting insulin and *p* < 10^−4^ for all other traits). SNPs in linkage disequilibrium (LD) (*r* ^2^ > 0.2) with a known genome-wide significant associated lead SNP for the given metabolic trait were excluded. Phased data from the 1000 Genomes project were used to estimate LD. For the lipid traits we additionally defined a known associated locus as the region spanning 250 Kb up- and downstream of the known associated SNP. All SNPs within these regions were excluded for follow-up.

### Stage 3 genotyping

Forty-five SNPs were selected for stage 3 replication and were genotyped in up to 63,896 individuals from seven centres (ESM Table 3). The trait-specific sample sizes are described in ESM Table 6.

### Meta-analysis of stage 3 data and meta-analysis stage 2 and stage 3 results

First, association results from the seven centres of stage 3 were combined by meta-analysis to obtain an overall replication result. Second, the Danish discovery data from stage 2 were meta-analysed with the seven replication centres to obtain an overall combined result. The effect for each SNP was estimated by inclusion in a fixed-effects meta-analysis using METAL [24]. For quantifiable traits an overall *z* statistic relative to each reference allele was estimated based on *p* values and direction of effects adjusted for the number of individuals in each sample. For dichotomous traits the estimate was weighted according to the estimated SEs by using the inverse corresponding SE.

In meta-analysis of all data, we applied a Bonferroni correction for the number of SNPs and the number of traits analysed (*p*_corrected_ = 0.05/(70,182 × 12) = 5.9 × 10^−8^). This correction is conservative as we did not take into account SNP or trait correlations. The corrected threshold is close to conventional genome-wide significance level (*p* = 5 × 10^−8^).

### Gene expression analysis

Gene expression levels of *CD300LG*, *COBLL1*, *MACF1*, *ACP1*, *ZFAND2B*, *GPSM1*, *PRRC2A* and *GRB14* were quantified by TaqMan real-time PCR (Applied Biosystems, Foster City, CA, USA) in a human tissue mRNA panel including aorta, leucocytes, total brain, hippocampus, hypothalamus, pituitary gland, colon, total small intestine, jejunum, ileum, adipose tissue, kidney, liver, pancreas, skeletal muscle and placenta (ClonTech Laboratories, Mountain View, CA, USA).

### Further information

Additional description of methods can be found in the ESM Methods & Results.

## Results

### Whole exome sequencing (stage 1)

The workflow of the project is shown in Fig. 1. In stage 1, 2,000 individuals were exome sequenced to a median coverage per individual of 91% of the target region. Hereof, 986 metabolic cases and 988 controls with an average depth of 8× fulfilled quality filtering (ESM Fig. 2, ESM Table 4). In total, 70,182 low-frequency and common variants with an estimated MAF above 1% were identified (Table 1, ESM Table 5, ESM Fig. 8). In the initial association analyses with case–control status a general inflation of the test statistics was observed. Application of a stringent set of SNP filtering criteria to remove the bias and restriction of the association analysis to the 48,035 SNPs that fulfilled all filtering criteria resulted in a low inflation rate (λ_GC_ 1.05) (ESM Fig. 9). As expected no strong associations were found but instead, as part of the study design, a number of SNPs in the tail of the *p* value distribution were selected for genotyping in stage 2.

### Association with metabolic traits in Danish individuals (stage 2)

To follow up on the outcome of exome sequencing-based SNP discoveries and association analysis in a larger sample set, 16,192 SNPs were analysed in 15,989 Danes. Of these SNPs, 54% were not present on any of the most commonly used GWAS arrays and 50% were not imputable from GWAS data using HapMap as reference panel.

Initially we performed an enrichment analysis for the SNPs nominally associated with case–control status in exome sequencing data (*p* < 0.05, *n* = 1,681). These analyses showed an excess of low *p* values in stage 2 association results for type 2 diabetes (ESM Figs 10, 11). The estimated fraction of associated SNPs was 3.1%, corresponding to 52 expected true associations among the 1,681 SNPs, yet some might be associated due to LD with the same causal variant. No excess of low *p* values were found in analyses of obesity and hypertension (ESM Figs 12, 13).

**Fig. 2:**
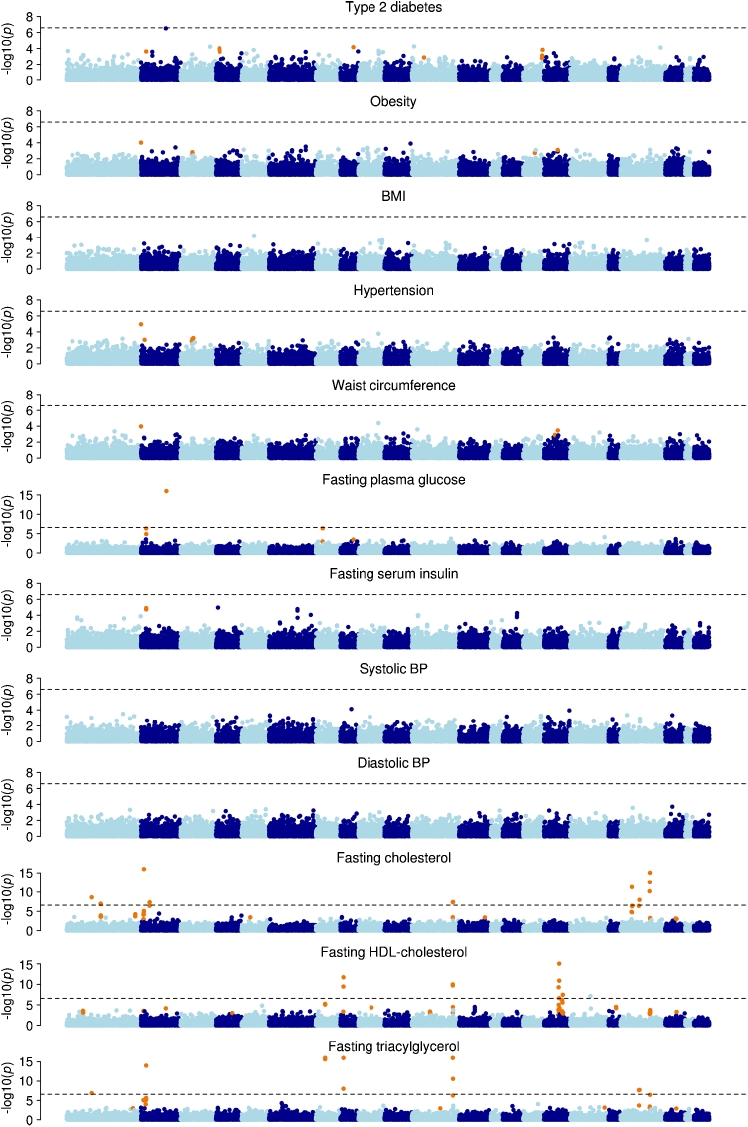
Manhattan plots of 16,192 SNPs for 12 metabolic traits in up to 15,989 Danish individuals (stage 2). For each of the traits the −log_10_(*p*) was plotted against the chromosome position. SNPs that have been established as known genome-wide associated signals for each trait are marked in orange. The dotted line indicates Bonferroni correction significance threshold corrected for 16,192 SNPs and 12 traits. The association analyses were performed with logistic or linear regression adjusted for first principal component and sex. All *p* values were corrected by genomic control

In further analyses of the 16,192 SNPs all 15,989 individuals were included to increase statistical power and SNPs for follow-up in stage 3 were prioritised from examinations of three binary traits (type 2 diabetes, obesity and hypertension) and nine quantifiable traits (BMI, waist circumference, systolic and diastolic BP, fasting levels of plasma glucose and serum insulin, total cholesterol, HDL-cholesterol and triacylglycerol). In analyses of the 12 metabolic phenotypes the strongest novel associations were demonstrated between the *CD300LG* R82C missense variant and fasting HDL-cholesterol (β = −0.18, *p* = 7.2 × 10^−8^) and the *COBLL1* rs7607980 variant and type 2 diabetes (OR 0.80, *p* = 7.2 × 10^−8^). These were the only associations with *p* values below 10^−6^ while a number of potential associations with uncorrected *p* values below 10^−4^ were detected (Fig. 2, ESM Fig. 7).

SNPs showing potential novel association with one or more of the 12 traits were selected for replication in stage 3. This selection yielded 51 associations for eight traits covered by 45 unique SNPs (ESM Table 7). SNPs were selected from association results of type 2 diabetes (11 SNPs), obesity (nine SNPs), BMI (three SNPs), waist circumference (one SNP), fasting glucose (seven SNPs), fasting insulin (eight SNPs), fasting HDL-cholesterol (two SNPs) and fasting triacylglycerol (one SNP). A SNP in *ELOVL3* showed potential association with both type 2 diabetes, fasting plasma glucose and waist circumference while two SNPs in *ACP1* and *SLC27A4* were selected from analyses of obesity, BMI and waist circumference (ESM Table 8). We did not identify any SNPs from association results of hypertension, systolic and diastolic BP and fasting total cholesterol that fulfilled the selection criteria.

### Replication of selected associations in European samples (stage 3)

The 45 SNPs covering the 51 potential associations discovered in stage 2 were genotyped in up to 63,896 Europeans for replication in stage 3 (Fig. 1, ESM Table 6). Meta-analysis of stage 3 replication data showed nominal replication (*p* < 0.05) for the same trait in a consistent direction for seven of the 51 selected associations (ESM Table 9). In meta-analysis of Danish stage 2 data and stage 3 replication data, three SNPs were associated at *p* < 5.9 × 10^−8^ (Table 2, ESM Figs 14, 15). A low-frequency (MAF 3.5%) non-synonymous (R82C) polymorphism in *CD300LG* was associated with lower fasting levels of serum HDL-cholesterol while two common (MAF 12.5% and 23.4%, respectively) non-synonymous polymorphisms in *COBLL1* and *MACF1* were associated with type 2 diabetes (Table 2). All three replicated SNPs in *CD300LG*, *COBLL1* and *MACF1* were selected for stage 2 based on their annotation (missense) and the *COBLL1* variant was also selected based on its stage 1 association *p* value. The effect of the minor allele of *CD300LG* R82C on fasting HDL-cholesterol in repeated analyses in replication cohorts without rank normalisation of HDL-cholesterol was 0.051–0.072 mmol/l. Further in-silico replication data for *COBLL1* and *MACF1* were obtained from existing GWAS meta-analysis data [2] (ESM Table 10) while no previous association data exists for the *CD300LG* variant. In additional analyses of other metabolic traits, the *CD300LG* variant also associated (*p* < 0.001) with increased fasting serum triacylglycerol while the *MACF1* rs2296172 variant also associated with decreased fasting HDL-cholesterol (ESM Table 11). No secondary associations were seen for the *COBLL1* rs7607980 variant. Analyses of gene expression in a tissue panel showed that *CD300LG* is expressed in adipose tissue, skeletal muscle and placenta (ESM Fig. 16). Data showed that *COBLL1* is expressed in pancreatic islets and kidney and to some degree in skeletal muscle, liver and adipose tissue while *MACF1* is expressed in more tissues including pancreas and skeletal muscle.

**Table 2:** Genome-wide significant associations with metabolic phenotypes for coding polymorphisms

Trait	Basic information					Discovery (stage 2)		Replication (stage 3)			Combined	
	rs (dbSNP 129)	Chr-position (build 36)	Gene	Effect/other allele	EAF (%)	*n*	*p* value	*n*	Estimate	*p* value	*n*	*p* value
Fasting serum HDL-cholesterol	None	17-39281652	*CD300LG*	T/C	3.5	13,063	7.2 × 10^−8^	20,822	−0.14 (0.027)	1.5 × 10^−7^	33,885	8.5 × 10^−14^
Type 2 diabetes	rs7607980	2-165259447	*COBLL1*	C/T	12.5	12,177	3 × 10^−7^	36,407	0.88 (0.84–0.93)	5.4 × 10^−6^	48,584	1.2 × 10^−11^
Type 2 diabetes	rs2296172	1-39608404	*MACF1*	G/A	23.4	12,175	0.00065	63,896	1.10 (1.06–1.14)	5.8 × 10^−7^	76,071	8.2 × 10^−10^

Estimates are OR (95% CI) for binary variables (type 2 diabetes) or beta (SE) on a rank normalised scale for quantifiable traits (fasting serum HDL-cholesterol). Reported estimates are based on replication (stage 3) data. Estimates of effects and *p* values for binary traits in replication and combined meta-analyses were calculated based on effect size and SE where effect size was weighted according to the SEs by using the inverse corresponding SE. For quantifiable traits an overall *z* statistic was calculated relative to each reference allele estimated based on *p* value and direction of effect adjusted for the number of individuals in each sample. Alleles are given on the positive strand. Chromosome and position for SNPs are stated according to Build 36.3 (hg18). More details are given in ESM Table 9 and ESM Figs 14, 15

Chr, chromosome; EAF, effect allele frequency

## Discussion

To discover novel associations between coding polymorphisms with a MAF above 1% and common metabolic traits we sequenced the exomes of 1,974 Danes to a depth of 8× and subsequently performed a two-stage follow-up in 15,989 Danes and in a further 63,896 Europeans. We identified a low-frequency amino-acid polymorphism in *CD300LG* associated with fasting HDL-cholesterol and two common amino-acid polymorphisms in *COBLL1* and *MACF1* associated with type 2 diabetes.

While the outcome of this comprehensive study may seem modest it remains a first-line report of challenges with large-scale next-generation sequencing studies of complex traits. Strengths of the study include the thorough replication in European samples, bringing high confidence in the reported associations, yet notable drawbacks are related to the early-stage exome capture technology and sequencing with a relatively low depth; together with bias in the sequencing data, in part coming from lack of sample randomisation, leading to the inability to assess the impact of rare variation alone or as gene-based combinations.

The effect of *CD300LG* R82C on fasting HDL-cholesterol was higher than all but one of the GWAS-identified HDL-cholesterol-associated variants [10]. CD300LG is a type I membrane glycoprotein that contains a single immunoglobulin V-like domain [25, 26]. The protein has been proposed to serve multiple functions, including endocytosis of various immunoglobulins [25] and mediation of L-selectin-dependent lymphocyte rolling [26], and has been shown to bind a broad range of polar lipids [27]. In-silico prediction by PolyPhen and SIFT indicated that the non-conservative R82C substitution is damaging to protein function suggesting that R82C could be the functional variant in this locus. Obviously, functional studies are needed to provide further evidence of the role of this variant.

The variants N939D in *COBLL1* and M2290V in *MACF1* were associated with type 2 diabetes, yet non-coding SNPs in these loci have previously been associated with other metabolic phenotypes [3, 6, 10, 28]. In the *COBLL1* locus (ESM Fig. 17) the intergenic rs10195252 is reported to associate with fasting triacylglycerol [10] and waist-to-hip ratio in women while the intergenic rs3923113 was reported to associate with type 2 diabetes in a GWAS in individuals of South Asian ancestry [3]. *COBLL1* N939D found here is in partial LD with these variants (HapMap release 27: *r* ^2^ = 0.18 and *r* ^2^ = 0.20, respectively) and conditional analysis showed that *COBLL1* N939D carries the effect on type 2 diabetes when conditioning on rs10195252 or rs3923113. Two SNPs in the region have been implicated in the regulation of fasting circulating levels of triacylglycerol and HDL-cholesterol [10]. *COBLL1* N939D is in high LD (HapMap release 27: *r* ^2^ = 0.98) with the HDL-cholesterol-associated variant [10], and we confirmed the association with HDL-cholesterol for this locus. *COBLL1* N939D is also in high LD (HapMap release 27: *r* ^2^ = 0.97) with rs12328675 reported to associate with fasting triacylglycerol; however, we observed no association with fasting triacylglycerol levels. The M2290V variant in *MACF1* was shown to increase the risk of type 2 diabetes (Table 2) and subsequent analyses of related metabolic phenotypes showed that the same allele also decreased fasting serum HDL-cholesterol levels. *PABPC4* rs4660293, which is correlated with *MACF1* rs2296172 (HapMap CEU release 27: *r* ^2^ = 0.64) has previously been reported to associate with HDL-cholesterol [10]. The biological functions of the associated variants in *COBLL1* and *MACF1* are unknown; variants in the *COBLL1* locus may, however, influence expression of nearby *GRB14* to change insulin sensitivity [6, 29].

In the present sequencing-based study initiated in early 2008 we applied exome sequencing to a depth of 8× in 1,974 individuals to discover variants associated with metabolic traits. This and other reports [17, 18] constitute the first indications that exome sequencing is a useful tool in complex traits genetics. Yet, for studying low-frequency and common variation not captured by the standard GWAS design the most cost-effective design for the near future may be to impute variants in standard SNP chip genotyped samples based on whole-genome sequence reference panels such as data from the 1000 Genomes Project [30]. In this context, studies based on SNP chip genotyping and imputation based on a local genome-wide sequencing reference set have lately been published [12]. Interestingly, a recently published report suggested that extremely low-pass whole-genome sequencing (0.1–0.5×) and imputation from 1000 Genomes Project reference panel is more cost-efficient than array genotyping for the study of variants with MAF above 1% [31]. While these approaches may work for low-frequency and common variation, the study of rare variation (MAF <0.5–1%) necessitates resequencing to capture the spectrum of variation. As highlighted by restraints in the present study, issues of sequencing depth, sample size and unbiased data generation are of foremost importance. Deep unbiased exome sequencing will also allow for burden test analyses of the combined impact on phenotype of multiple rare and low-frequency variants in a given locus or in other functional units such as a biologically relevant pathway [32].

In conclusion, we performed medium-depth exome sequencing in 2,000 individuals with follow-up in up to 76,071 Europeans and discovered three amino-acid polymorphisms with a frequency above 1% associated with specific metabolic phenotypes. Therefore, low-frequency and common coding polymorphisms with impact on metabolic traits do exist but they do not seem to be widespread. This study serves as an indication of the utility of exome sequencing in complex metabolic traits.

## Electronic supplementary material

Below is the link to the electronic supplementary material.

ESM List of members of the D.E.S.I.R. Study Group and the DIAGRAM Consortium (PDF 221 kb)

ESM Methods and Results (PDF 562 kb)

ESM Fig. 1 (PDF 200 kb)

ESM Fig. 2 (PDF 263 kb)

ESM Fig. 3 (PDF 223 kb)

ESM Fig. 4 (PDF 228 kb)

ESM Fig. 5 (PDF 339 kb)

ESM Fig. 6 (PDF 224 kb)

ESM Fig. 7 (PDF 474 kb)

ESM Fig. 8 (PDF 241 kb)

ESM Fig. 9 (PDF 330 kb)

ESM Fig. 10 (PDF 334 kb)

ESM Fig. 11 (PDF 473 kb)

ESM Fig. 12 (PDF 481 kb)

ESM Fig. 13 (PDF 473 kb)

ESM Fig. 14 (PDF 341 kb)

ESM Fig. 15 (PDF 332 kb)

ESM Fig. 16 (PDF 391 kb)

ESM Fig. 17 (PDF 420 kb)

ESM Table 1 (PDF 200 kb)

ESM Table 2 (PDF 199 kb)

ESM Table 3 (XLS 72 kb)

ESM Table 4 (PDF 197 kb)

ESM Table 5 (PDF 198 kb)

ESM Table 6 (PDF 317 kb)

ESM Table 7 (PDF 321 kb)

ESM Table 8 (PDF 233 kb)

ESM Table 9 (PDF 348 kb)

ESM Table 10 (PDF 290 kb)

ESM Table 11 (PDF 296 kb)

## Abbreviations

GWAS: Genome-wide association study
LD: Linkage disequilibrium
MAF: Minor allele frequency
SNP: Single-nucleotide polymorphism
