# Supplementary material for: Exome sequencing-driven discovery of coding polymorphisms associated with common metabolic phenotypes
Source: Diabetologia. 2012 Nov 19;56(2):298–310. doi: 10.1007/s00125-012-2756-1 (PMC3536959; doi:10.1007/s00125-012-2756-1)
Supplement: Supplementary file 2 — (PDF 562 kb) [file 125_2012_2756_MOESM2_ESM.pdf]

ELECTRONIC SUPPLEMENTARY MATERIAL

Exome sequencing-driven discovery of variants associated with common metabolic phenotypes

A. Albrechtsen*, N. Grarup*, Y. Li*, T. Sparsø*, G. Tian*, H. Cao, T. Jiang, S. Y. Kim, T. Korneliussen, Q. Li, C. Nie, R. Wu, L. Skotte, A. P. Morris, C. Ladenvall, S. Cauchi, A. Stančáková, G. Andersen, A. Astrup, K. Banasik, A. J. Bennett, L. Bolund, G. Charpentier, Y. Chen, J. M. Dekker, A. S.F. Doney, M. Dorkhan, T. Forsen, T. M. Frayling, C. J. Groves, Y. Gui, G. Hallmans, A. T. Hattersley, K. He, G. A. Hitman, J. Holmkvist, S. Huang, H. Jiang, X. Jin, J. M. Justesen, K. Kristiansen, J. Kuusisto, M. Lajer, O. Lantieri, W. Li, H. Liang, Q. Liao, X. Liu, T. Ma, X. Ma, M. Manijak, M. Marre, J. Mokrosiński, A. D. Morris, B. Mu, A. A. Nielsen, G. Nijpels, P. Nilsson, C. N.A. Palmer, N. William Rayner, F. Renström, R. Ribel-Madsen, N. Robertson, O. Rolandsson, P. Rossing, T. W. Schwartz, P. Slagboom, M. Sterner, D.E.S.I.R. Study Group, M. Tang, L. Tarnow, the DIAGRAM Consortium, T. Tuomi, E. van't Riet, N. van Leeuwen, T. V. Varga, M. A. Vestmar, M. Walker, B. Wang, Y. Wang, H. Wu, F. Xi, L. Yengo, C. Yu, X. Zhang, J. Zhang, Q. Zhang, W. Zhang, H. Zheng, Y. Zhou, D. Altshuler, L. M. t Hart, P. W. Franks, B. Balkau, P. Froguel, M. I. McCarthy, M. Laakso, L. Groop, C. Christensen, I. Brandslund, T. Lauritzen, D. R. Witte, A. Linneberg, T. Jørgensen, T. Hansen, J. Wang, R. Nielsen, O. Pedersen

TABLE OF CONTENTS

ESM METHODS & RESULTS 3

1. Study populations 3

1.1. Description of the Danish study samples applied in stage 1 discovery and stage 2 genotyping and association studies 3

1.2. Application of Danish study samples in stage 1 exome sequencing and stage 2 Illumina iSelect BeadChip follow-up genotyping 4

1.3. Study samples applied in external replication studies (Stage 3) 5

1.4. Biochemical and anthropometric measures 9

2. Exome sequencing of 2,000 individuals 9

2.1. Preparation of DNA for exon capturing and sequencing 9

2.2. Outcome of exome sequencing of 2,000 samples 10

2.3. Quality control of outcome of exome sequencing 10

2.4. Individual sample quality control using called genotypes 11

2.5. SNP detection and allele frequency estimation 11

2.6. SNP filters for association 13
2.7. Association testing 14
3. Stage 2: Genotyping and association mapping in of 16,192 coding variants in 15,989 individuals .15
3.1. SNP selection from stage 1 for Illumina iSelect genotyping in 16,988 Danish individuals 15
3.2. Illumina iSelect genotyping....15
3.3. Statistical analysis of stage 2 data 16
3.4. Results of stage 2 genotyping and association studies....17
4. Stage 3: Validation of selected tentative associations in up to 63,896 European individuals....18
4.1. Selection of SNPs for validation in stage 3 genotyping....18
4.2. Stage 3 statistical analysis....18
5. Gene expression profiling 18
5.1. Tissue RNA samples 19
5.2. cDNA synthesis 19
5.3. Real-time PCR 19
6. URLs 19
7. References 19

ESM METHODS & RESULTS

1. Study populations

1.1. Description of the Danish study samples applied in stage 1 discovery and stage 2 genotyping and association studies

Clinical and biochemical characteristics for all study sample groups are shown in ESM Table 1. All individuals participating in the discovery studies were of Danish nationality. Informed written consent was obtained from all study participants. The studies were conducted in accordance with the Declaration of Helsinki II and were approved by the local Ethical Committees.

1.1.1. INTER99

The Inter99 cohort is a randomized, non-pharmacological intervention study for the prevention of ischaemic heart disease, conducted on 6,784 randomly ascertained participants aged 30 to 60 years at the Research Centre for Prevention and Health in Glostrup, Denmark [1] (ClinicalTrials.gov: NCT00289237). An oral glucose tolerance test (OGTT) was performed with measurement of plasma glucose and serum insulin at fasting and 30 and 120 min after glucose intake. Subsequently, 6,094 participants of Danish nationality and with available DNA were classified as having normal glucose tolerance (*n*=4,525), impaired fasting glycaemia (*n*=504), impaired glucose tolerance (*n*=693), screen-detected type 2 diabetes (*n*=253), or previously diagnosed type 2 diabetes (*n*=119) according to World Health Organization (WHO) 1999 criteria. Detailed characteristics of Inter99 have been published previously [1-3].

1.1.2. HEALTH2006

Health2006 is a population-based epidemiological study of general health, diabetes and cardiovascular disease of individuals aged 18-74 years [4]. In addition to fasting biochemistry these individuals have had step test to objectively quantify physical fitness. Health2006 was conducted at the Research Centre for Prevention and Health in Glostrup, Denmark.

1.1.3. TYPE 2 DIABETES CASE-CONTROL INDIVIDUALS ASCERTAINED AT STENO DIABETES CENTER

A sample of clinical-onset type 2 diabetes patients and non-diabetic control individuals were ascertained at the outpatient clinic at Steno Diabetes Center, Copenhagen. An OGTT was performed in all control individuals to exclude individuals with unknown diabetes or states of prediabetes according to WHO 1999 criteria [5].

1.1.4. ADDITION STUDY SCREENING COHORT

The Danish ADDITION Study (Anglo–Danish–Dutch Study of Intensive Treatment in People with Screen-Detected Diabetes in Primary Care) is a high-risk screening and intervention study for type 2 diabetes in general practice sampled by Department of General Practice at University of Aarhus, Denmark (ClinicalTrials.gov ID-no: NCT00237548) [6]. The 8,662 participants from the initial screening cohort with available DNA included 1,626 participants with screen-detected and untreated type 2 diabetes and 7,036 non-diabetic subjects. Patients with type 2 diabetes were diagnosed by two independent diabetic values at baseline investigation or at one year follow-up.

1.1.5. VEJLE BIOBANK

Vejle Biobank is a sample of clinical-onset type 2 diabetes patients and non-diabetic control individuals with similar age and sex distribution examined at Vejle Hospital during a three year period. Control individuals were non-diabetic by self-report and by a fasting plasma glucose test according to WHO 1999 criteria [5].

1.2. Application of Danish study samples in stage 1 exome sequencing and stage 2 Illumina iSelect BeadChip follow-up genotyping

Exome sequencing was performed in 1,000 metabolic cases all of whom were enriched for cardiovascular risk factors (type 2 diabetes, obesity and hypertension) and 1,000 individuals who were lean, glucose-tolerant and had normal blood pressure. Cases were selected from the study samples described above based on presence of all of the following criteria: 1) diagnosis of type 2 diabetes with age at diagnosis between 30 and 70 years of age, 2) body mass index (BMI) > 27.5 kg/m^2, 3) waist circumference > 94 cm (men) or > 80 cm (women), and 4) hypertension defined as a measured blood pressure above 140/90 mmHg or use of anti-hypertensive medication. Diabetes patients were excluded from the investigation if they displayed one of the following: a history of pancreatitis or haemochromatosis, GAD65 antibody positivity, fasting serum C-peptide level below 150 pmol/l or type 1 diabetes among 1st degree relatives. Control individuals were selected based on presence of all of the criteria: 1) normal glucose tolerance at fasting and after 2 hrs during an OGTT applying American Diabetes Association criteria [7] (fasting plasma glucose < 5.6 mmol/l and 2 hrs post OGTT plasma glucose < 7.8 mmol) and 2) BMI < 27.5 kg/m^2 and 3) age at investigation above 35 years. Clinical and biochemical characteristics of 2,000 exome sequenced individuals are shown in ESM Table 2. These data demonstrated clinical differences between cases and controls in a range of clinical metabolic parameters including measures of glycaemia, measures of obesity, fasting lipid levels and blood pressure. Metabolic cases involved in exome sequencing were ascertained from Steno Diabetes Center (*n*=610), the ADDITION study (*n*=354) or from Inter99 (*n*=36). Control individuals were ascertained from Inter99 (*n*=851) or from Steno Diabetes Center (*n*=149). In stage 2 association studies of 16,192 SNPs, three binary and nine quantitative traits were investigated in the study samples described above and shown in ESM Table 1. A detailed clinical and biochemical description of the individuals involved in each analysis is shown in ESM Table 1.

1) Type 2 diabetes: Clinical-onset and screen-detected type 2 diabetes patients defined by WHO 1999 criteria [5] were selected from Steno Diabetes Center (*n*=1,798), the ADDITION study (*n*=1,869), Vejle Biobank (*n*=878), the Inter99 study (*n*=189) and Health2006 (*n*=120). Cases were excluded if they displayed one of the following: a history of pancreatitis or haemochromatosis, GAD65 antibody positivity, fasting serum C-peptide level below 150 pmol/l or type 1 diabetes among 1st degree relatives. Control individuals were selected from population-based study samples and had normal fasting plasma glucose (< 6.1 mmol/l)[5]. An OGTT was performed in 4,380 of 7,325 controls and these individuals also had normal 2 hrs plasma glucose (< 7.8 mmol/l) [5]. The control individuals were sampled from Inter99 (*n*=4,382), Health2006 (*n*=2,246), Vejle Biobank (*n*=448) and Steno Diabetes Center (*n*=249). In total, 4,854 cases and 7,325 control individuals were analyzed.
2) Obesity: A case-control analysis of obesity involved 5,488 cases with BMI ≥ 30 kg/m^2 and 4,851 controls with BMI < 25 kg/m^2 sampled from Inter99 (*n*-case=1,008, *n*-control=2,505), Health2006 (*n*-case=467, *n*-control=1317), ADDITION (*n*-case=2,854), Steno Diabetes Center (*n*-case=832, *n*-control=507) and Vejle Biobank (*n*-case=522, *n*-control=327).
3) Hypertension: A case-control analysis of hypertension involving 7,299 cases with blood pressure >140/90 mmHg and/or taking antihypertensive medication and 3,290 controls with blood pressure <140/90 mmHg and not taking antihypertensive medication.
4) BMI: BMI was studied as a quantitative trait in all available samples where individuals treated with insulin were excluded. The analysis included 14,819 individuals sampled from Inter99 (*n*=5,845),

Health2006 (*n*=2,899), ADDITION (*n*=3,705), Steno Diabetes Center (*n*=1,404) and Vejle Biobank (*n*=966).

5) Waist circumference: Waist circumference was studied as a quantitative trait in all available samples where individuals treated with insulin were excluded. The analysis included 14,538 individuals sampled from Inter99 (*n*=5,836), Health2006 (*n*=2,900), ADDITION (*n*=3,693), Steno Diabetes Center (*n*=1,068) and Vejle Biobank (*n*=1,041).
6) Fasting plasma glucose: In analysis of fasting plasma glucose all individuals with previously diagnosed diabetes were excluded. The analysis included 9,087 individuals sampled from Inter99 (*n*=5,760), Health2006 (*n*=2,601), Vejle Biobank (*n*=449) and Steno Diabetes Center (*n*=277).
7) Fasting serum insulin: In analysis of fasting serum insulin all individuals with previously diagnosed diabetes were excluded. The analysis included 8,419 individuals sampled from Inter99 (*n*=5,541), Health2006 (*n*=2,601) and Steno Diabetes Center (*n*=277).
8) Systolic blood pressure: In the analysis of blood pressure all individuals on antihypertensive medication (*n*=968) were excluded. The analysis involved 12,651 individuals.
9) Diastolic blood pressure: In the analysis of blood pressure all individuals on antihypertensive medication (*n*=968) were excluded. The analysis involved 12,651 individuals.
10) Fasting serum total cholesterol: In analysis of fasting serum total cholesterol all individuals treated with lipid-lowering medication were excluded. The analysis included 13,183 individuals sampled from Inter99 (*n*=5,782), Health2006 (*n*=2,710), ADDITION (*n*=3,659) and Steno Diabetes Center (*n*=1,032).
11) Fasting serum HDL-cholesterol: In analysis of fasting serum HDL-cholesterol all individuals treated with lipid-lowering medication were excluded. The analysis included 13,063 individuals sampled from Inter99 (*n*=5,780), Health2006 (*n*=2,710), ADDITION (*n*=3,590) and Steno Diabetes Center (*n*=983).
12) Fasting serum triacylglycerol: In analysis of fasting serum HDL-cholesterol all individuals treated with lipid-lowering medication were excluded. The analysis included 13,326 individuals sampled from Inter99 (*n*=5,786), Health2006 (*n*=2,902), ADDITION (*n*=3,661) and Steno Diabetes Center (*n*=977).

1.3. Study samples applied in external replication studies (Stage 3)

Clinical samples from six different European countries were investigated in replication studies of selected SNPs. Clinical characteristics of the replication cohorts are shown in ESM Table 3.

1.3.1. VEJLE BIOBANK

In replication studies further samples from Vejle Biobank were used. These individuals were ascertained as described in ESM Methods section 1.1.5. The biobank and studies were conducted in accordance with the Declaration of Helsinki II and were approved by the local Ethical Committees.

1.3.2. FRENCH

D.E.S.I.R.

The D.E.S.I.R. ("Data from an Epidemiological Study on the Insulin Resistance syndrome") cohort is a French cohort of middle-aged subjects (aged between 30 and 65 years at inclusion) who were clinically and biologically evaluated at inclusion and at 3, 6, and 9 years after the entry into the study [8]. Participants were recruited from volunteers insured by the French social security system, which offers periodic health examinations free of charge. They came from ten health examination centres in the western-central part of France. The protocol was approved by the Ethics Committee for the Protection of Subjects for Biomedical Research of Bicêtre Hospital.

Obese cases

The obese adults (OBA) are unrelated French Caucasians recruited by the CNRS UMR8199 laboratory and the Nutrition Department of Hotel-Dieu Hospital (Paris) from pedigrees with family history of obesity [9]. The protocol was approved by the Ethics Committee for the Protection of Subjects for Biomedical Research.

Type 2 diabetes cases

The group of type 2 diabetic subjects (Corbeil) consisted of unrelated French Caucasian patients of the Endocrinology-Diabetology Department at Corbeil-Essonnes Hospital [10]. The protocol was approved by the Ethics Committee for the Protection of Subjects for Biomedical Research.

1.3.3. FINNISH

The METSIM Study

The METSIM (Metabolic Syndrome in Men) study is a population-based cross-sectional study comprising a total of 10,197 men. Participants, aged from 45 to 70 years, were randomly selected from the population register of Kuopio town, Eastern Finland (population of 95,000) and examined within years 2005-2010. Every participant had one-day outpatient visit to the Clinical Research Unit at the University of Kuopio (presently named as University of Eastern Finland), including an interview on the history of previous diseases and current drug treatment, and an evaluation of glucose tolerance and cardiovascular risk factors. Fasting blood samples were drawn after 12 hrs of fasting followed by an OGTT. The study was approved by the Ethics Committee of the University of Kuopio and Kuopio University Hospital, and it was in accordance with the Helsinki Declaration. Height and weight were measured to the nearest 0.5 cm and 0.1 kg, respectively. BMI was calculated as weight (kg) divided by height (m) squared. Waist (at the midpoint between the lateral iliac crest and lowest rib) and hip circumference (at the level of the trochanter major) were measured to the nearest 0.5 cm. A 2-hr OGTT (75 g of glucose) was performed, and samples for plasma glucose and insulin were drawn at 0, 30 and 120 min.

1.3.4. BRITISH

1958 Birth Cohort (58BC)

The 1958 Birth Cohort (also known as the National Child Development Study) is a longitudinal study of individuals born in England, Wales and Scotland, during one week in 1958. From an original sample of over 17,000 births, survivors were followed up at ages 7, 11, 16, 23, 33 and 42 years. In a biomedical examination at 44-45 years, 9,377 cohort members were visited at home providing 7,692 blood samples of which 7,222 had available DNA. All subjects gave written informed consent and the project protocols were approved by the relevant research ethics committees in the UK.

UK Blood Services (UKBS)

The UKBS cohort consists of individuals recruited through the UK Blood Services (NHSBT in England, SNBTS in Scotland and WBS in Wales), forming a UK national repository of anonymized samples of DNA from 3,622 blood donors of which 3,139 had available DNA. All subjects gave written informed consent and the project protocols were approved by the relevant research ethics committees in the UK.

UK Type 2 Diabetes Genetics Consortium (WTD)

All cases and controls were of European White descent, living in the Tayside region of Dundee when recruited. The diagnosis of diabetes was based on either current prescribed treatment with diabetes-specific medication or, in the case of individuals treated with diet alone, laboratory evidence of hyperglycemia as defined by WHO. Patients were excluded if they had an established (clinical and/or molecular) diagnosis of monogenic diabetes (e.g. maturity-onset diabetes of the young or mitochondrial diabetes) or if they had been treated with regular

insulin therapy within one year of diagnosis. Controls had not been diagnosed with diabetes at the time of recruitment (or subsequently). A total of 13,663 samples had DNA available. This study was approved by the Tayside Medical Ethics Committee and informed consent was obtained from all subjects.

Warren 2 (W2)

The Diabetes UK Warren 2 repository Samples were collected in five main centres (Exeter, London, Oxford, Norwich and Newcastle) but with nationwide representation. All were of British/Irish European descent, diagnosed between age 25 and 75. Approximately 30% of cases were explicitly recruited as part of multiplex sibships, and ~25% represented the type 2 diabetes offspring within parent-offspring “triads” or “duos” (that is, offspring from sibships with only one parent available). The remainders were recruited as isolated cases ascertained for early age at diagnosis compared to the population distribution. Diagnosis of diabetes was based on either current prescribed treatment with diabetes-specific medication or, in the case of those treated with diet alone, historical or contemporary laboratory evidence of hyperglycaemia (as defined by WHO). Other forms of diabetes were excluded by standard clinical criteria based on personal and family history giving 2,067 samples with DNA available. Informed consent for participation was obtained from all subjects and relatives, after approval had been granted by the relevant Research Ethics Committees.

1.3.5. SCANDINAVIAN

MDC cardiovascular cohort (MDC-CC)

The MDC study is a population-based cohort with baseline examination in 1991-1996 and consists of 28,449 individuals [11]. Eligible participants were men born between 1923 and 1945 (age range 46 to 73 years) and women born between 1923 and 1950 (age range 45 to 73 years) with Swedish reading and writing skills. From this cohort, 6,103 patients were randomly selected and referred to as the MDC cardiovascular cohort (MDC-CC) [12]. These patients were re-evaluated to characterize cardiovascular and metabolic risk factors. Diabetes mellitus at baseline was defined as self-report of a physician diagnosis or use of diabetes medication or fasting blood glucose ≥ 6.1 mmol/l (corresponding to fasting plasma glucose concentration of ≥ 7.0 mmol/l). 2,423 individuals free from diabetes at baseline were included in the current study. The study protocols were approved by the ethics committee of Lund University. All participants provided written informed consent.

Scania Diabetes Registry (SDR)

A Diabetes Registry in Southern Sweden (the Scania Diabetes Registry) was initiated in 1996 and hitherto 7,433 patients with different kinds of diabetes have been registered at the Department of Endocrinology, University hospital MAS, Malmö, Sweden, the Trelleborg hospital or health care centres in the Malmö and Trelleborg regions. The registry was initiated with the aim to 1) collect information needed for a more precise classification of diabetic subgroups, 2) identify genetic variants predisposing to micro- and macrovascular complications 3) study how identified bio- and genetic markers can predict development and progression of complications and 4) identify means to prevent the development of these complications. 3,341 subjects with type 2 diabetes and Scandinavian ethnicity were included in the current study. All patients gave their informed consent to be listed and the registry was approved by the Swedish Data Inspection Board. The Ethics Committee of Lund University approved the study.

All New Diabetics in Scania (ANDIS)

ANDiS is a regional research project in southern Sweden that was initiated in 2008. The registry aims to include all newly diagnosed patients at all ages in the Scania region during 2009-2013 and to describe the spectrum of diabetes subgroups in southern Sweden. When complete, the aim is to link genetic and phenotypic information at diagnosis of diabetes to outcome data and data on response to treatment. At present all 159 health care

centres and hospitals throughout the Scania region are participating in this effort. 1,422 individuals with type 2 diabetes and with Scandinavian ethnicity were included in the current study. All participants give their written informed consent to participate in the registry. The regional ethical review board in Lund approved the use of participants in the ANDiS registry for genetic analyses.

Prevalence, Prediction and Prevention of diabetes (PPP-Botnia)

The PPP-Botnia is a population-based study from the Botnia region in Finland. It includes 5,208 individuals (approximately 7% of the population) identified through the Population Registry and aged 18-75 years [13]. The aim of the study is 1) to study genetic and non-genetic risk factors for type 2 diabetes, 2) investigate the prevalence of glucose abnormalities and the metabolic syndrome in the adult population and 3) to use this information for prediction and prevention of the disease. Diagnosis of diabetes was confirmed from subject records or on the basis of a fasting plasma glucose concentration ≥ 7.0 mmol/l and/or 2 hr glucose ≥ 11.1 mmol/l. 4,049 normal glucose tolerant subjects were included in the current study. The participants gave their written informed consent and the study protocol was approved by the Ethics Committee of Helsinki University Hospital, Finland.

DIabetes REgistry in VAasa (DIREVA)

DIREVA is a collaborative project between Vaasa Central Hospital, Helsinki University and the Botnia project. The design of the registry is similar to the ANDiS registry (above) and the aim is to include all diabetes patients at all ages in the Vaasa region and create a permanent registry. In the Osthrobothian coastal district, two hospitals, primary care centres from 15 counties and three larger private care centres participate in the register. All participants gave their written informed consent to participate in the registry. 1,608 individuals with type 2 diabetes and with Scandinavian ethnicity (including Finland) were included in the current study. The ethics committee of the Vaasa Hospital district approved the use of participants in the DIREVA registry for genetic analyses.

1.3.6. DUTCH

The New Hoorn Study

The NHS study is a population based study in the West-Friesland region of the Netherlands which aims to identify risk factors for type 2 diabetes. Details have been described previously [14, 15]. Definition of glucose tolerance status in the NHS cohort was based on WHO criteria after a fasted OGTT. In addition, known diabetes was defined by the use of insulin or oral glucose lowering agents and (self-) reported known diabetes. From this study we enrolled 2,326 Caucasian subjects with normal glucose tolerance.

Diabetes Care System West Friesland

Furthermore we included participants with type 2 diabetes from the DCS West-Friesland study (*n*=7,515). The DCS provides diabetes care to type 2 diabetes subjects living in the same geographical region of the Netherlands as the NHS study [15-17]. In addition to the care of the general practitioners and their practice nurses, an extensive diabetes management program, is implemented. In short, new and existing type 2 diabetes patients living in the West-Friesland region in the North-Western part of the Netherlands are referred to the DCS and each patient visits the diabetes research centre annually for a laboratory and physical examination and advice on disease management. At each visit blood is drawn for routine biochemical analysis and if consent is given, once for DNA isolation (*n*=3,421). From this cohort we included 3,061 Caucasian subjects with type 2 diabetes.

The research performed for this study in the NHS and the DCS West Friesland study were approved by the medical ethics committee of the VU University Medical Center, Amsterdam, the Netherlands and were conducted according the principles of the declaration of Helsinki.

1.3.7. GLACIER

The GLACIER Study is a prospective, population-based cohort study comprising 19,547 adults from the northern Swedish county of Västerbotten, nested within the Northern Sweden Health and Disease Study. All GLACIER participants underwent detailed health and lifestyle examinations as part of the Västerbotten Intervention Programme, an ongoing population-based prospective cohort study focused on type 2 diabetes, cardiovascular disease, and common cancers. Baseline examinations for GLACIER participants were undertaken from 1985 through 2004. All participants gave written informed consent and the Regional Ethical Review Board in Umeå approved all aspects of the study. Weight (to the nearest 0.1 kg) and height (to the nearest 1 cm) were measured with a calibrated balance-beam scale and a wall-mounted stadiometer, respectively, with participants wearing indoor clothing without shoes. BMI was calculated as weight in kilograms divided by height in meters squared (kg/m^2). Capillary blood was drawn after an overnight fast, and a second sample was drawn 2 h after a standard 75-g oral glucose load. Capillary plasma glucose concentrations were measured with a Reflotron bench-top analyzer (Roche Diagnostics Scandinavia AB).

1.4. Biochemical and anthropometric measures

1.4.1. STAGE 1 AND STAGE 2 STUDY POPULATIONS

For participants in the five Danish study groups (ESM Table 1) blood samples were drawn after a 10-hr overnight fast. In all study groups plasma-glucose was analyzed by a glucose oxidase method (Granutest, Merck, Darmstadt, Germany). HbA_{1C} was measured by ion-exchange high performance liquid chromatography (normal reference range: 4.1-6.4 %) and serum-insulin (excluding des(31, 32) and intact proinsulin) was measured using the AutoDELFIA insulin kit (Perkin-Elmer, Wallac, Turku, Finland). In the Inter99 study, Health2006 survey and Vejle Biobank, serum triacylglycerol, total serum cholesterol and HDL-cholesterol were analyzed using enzymatic colorimetric methods (GPO-PAP and CHOD-PAP; Roche Molecular Biochemicals, Mannheim, Germany) while the Hitachi 971 system (Roche Diagnostics GmbH, Mannheim, Germany) was used for the ADDITION participants.

In all study populations applied in stage 1 sequencing and stage 2 Illumina iSelect genotyping, height and body weight for all participants were measured in light indoor clothes and without shoes, and BMI was calculated as weight (kg)/(height $(m))^2$. Waist circumference was measured in the upright position midway between the iliac crest and the lower costal margin, and hip circumference was measured at its maximum.

1.4.2. STAGE 3 STUDY POPULATIONS

Biochemical measures for individuals involved in replication stage 3 study samples are shown in ESM Table 3.

2. Exome sequencing of 2,000 individuals

2.1. Preparation of DNA for exon capturing and sequencing

2.1.1. EXON-CAPTURING AND ILLUMINA SEQUENCING

Exome capture and Illumina sequencing was performed on DNA from the 2,000 individuals as previously described [18]. On average, the sequencing depth per sample was 11× and 95.5% targeted bases were covered at least by one read.

2.1.2. ALIGNMENT OF READS USING SOAP-ALIGNER

We analyzed the reads that were generated by Illumina Genome Analyzer and discarded linker and adapter sequences that were introduced in experimental process, which resulted in a pre-processed dataset called "effective reads" (ESM Table 4). The effective reads were aligned to the human reference genome (assembly hg18, NCBI build 36.3) using SOAPaligner (http://soap.genomics.org.cn) with parameters set to "-a -D -o -r 1 -t -c -f 4". All "unique mapped reads" (defined as reads that had a single best alignment hit with a minimum number of mismatches) were used further for quality control, SNP identification and subsequent association analysis.

2.2. Outcome of exome sequencing of 2,000 samples

2.2.1. DATA PRODUCTION SUMMARY

After the alignment the total length of the captured target region was 34.1 Mb, including nearly all well-annotated exons as well as parts of other elements in 21,810 non-redundant genes and their alternative splicing forms. Hereafter, the full set of target regions is defined as the exome. Note that these elements are located on both strands of the DNA and they may be partially overlapping. As a result, the total length does not add up to 34.1 Mb.

The sequencing depth was calculated based on all "uniquely mapped reads" including low complexity sequences which have multiple best hits but were randomly aligned onto the exome region. Sequencing depth of the target region was defined by dividing all the uniquely mapped read bases by the length of the whole target region. The data production of all the raw reads including yield, length, and average depths are summarized in ESM Table 4. The exome coverage was defined by the proportion of the target region that was covered by at least one uniquely read base. Most samples had a coverage of more than 95% across the exome, suggesting that the exome capturing was successfully performed and that the exome target region was sufficiently covered (ESM Figure 1). To further investigate the evenness of the exome coverage, we examined the distribution of per-site sequencing depth (ESM Figure 2A). The number of reads that covered each base in the exome target region was counted and this was expected to follow a distribution that is theoretically a Poisson distribution. Most of the samples had a depth distribution that approximately followed a Poisson distribution shape, demonstrating that the exome-capturing target region was evenly sampled.

2.3. Quality control of outcome of exome sequencing

To ensure high-quality and high-throughput quality control of sequencing data several steps were taken. To remove low quality bases a minimum base quality score of Q20 was chosen (1% error rate by quality score definition). For comparison we plotted the distribution of per site sequencing depth after removal of all bases with a low quality (Q20) as well as all reads that were not unique (i.e. the reads had been mapped to more than one site during the alignment) (ESM Figure 2B). The same Poisson distribution was observed and based on the above arguments we concluded that applying a strict quality threshold of Q20 to the 2,000 Danish exomes resulted in a reliable dataset. Consequently, a Q20 threshold was applied in all further analysis. Due to the use of the Q20 threshold and multiple hits restrictions approximately 20% of the data was discarded and the average depth per site declined from 11X to 8X. Importantly, a few samples (approximately 1%) had an extremely high average depth of more than 25X clearly disturbing the average depth in each individual. In

addition, the median coverage (the amount of exome covered with at least one read) also decreased. The proportion of exomes (ESM Figure 2A) for sites with zero depth in unfiltered sequencing data showed a medium coverage of 1.00-0.04=0.96. After Q20 filtering, the low quality reads were excluded and the median coverage decreased to 0.91 (ESM Figure 2B).

2.4. Individual sample quality control using called genotypes

In general, inferring genotypes from low-coverage sequencing data is problematic due to the large uncertainties which are introduced in the genotypes. However, in the current section of the study called genotypes were used for the purpose of making individual quality control by comparing in-house genotyping data from previous studies with the called genotypes for these sites. The genotype calling is explained in ESM Methods section 2.5.7. We chose to work with genotypes with a posterior probability above 99% to ensure reliable genotype calls.

35 SNPs previously genotyped in the 2,000 individuals were used to investigate if some of the samples had been mislabelled or showed high genotype error. Using called genotypes from the sequencing data with a posterior probability above 99% the concordance with the previously genotyped SNPs was estimated. 23 individuals did not match the previously genotyped SNPs and were removed from the association study. Three individuals were sequenced twice and the sequencing data was merged.

Using called genotypes with a posterior probability above 99% we estimated the heterozygosity of the SNPs on the X-chromosome. Five individuals had disparate sex when comparing genotype and phenotype information and data from these individuals was consequently not used for the association analysis. As a result of the barcoding and sex comparison 1,974 samples (986 cases and 988 controls) were available for SNP detection and analysis.

2.5. SNP detection and allele frequency estimation

2.5.1. GENOTYPE LIKELIHOODS

We applied two different approaches to obtain genotype likelihoods. We used SOAPsnp [19] with the following parameters "-i -d -o -z '@' -F 1 -L 81 -T". These genotype likelihoods were utilized to call genotypes for the quality control. For the allele frequency estimation and association, we applied the method described by Kim *et al* [20]. This method estimates the type specific error rates for each pair of true and observed nucleotides directly from the putative polymorphic sites and uses these error rates combined with the base counts to obtain the genotype likelihoods using a multinomial model. The errors were estimated based on a random selection of 30,000 putative polymorphic sites. The putative SNP sites are enriched for errors because of the selection procedure that selects sites based on the observation of a high number of different bases. These are sites that either contain a high number of errors or are polymorphic. Even though these sites were enriched for mistakes due to the selection procedure, the type specific error rates were modest (all below 0.37%).

2.5.2. ESTIMATION OF ALLELE FREQUENCIES

As a first step in the identification of SNPs suitable for association testing the allele frequencies of all putative polymorphic sites were estimated based on the allele frequency estimator by Li *et al* [18]. A high error rate of 0.25% was assumed for all error types. Putative polymorphic sites were chosen as sites with an allele frequency above 0.25%.

After obtaining genotype likelihoods, we estimated the allele frequency using the maximum likelihood estimator developed by Kim *et al* [20], which does not require specification of a single minor allele for each site. This estimator assumes that the sites are diallelic, and takes the uncertainty in the minor allele into

account by summing likelihoods over all possible three minor alleles. This allele frequency estimator has a higher accuracy than the fast estimator of Li *et al* [18].

2.5.3. FALSE NEGATIVE RATE OF SNP IDENTIFICATION

The ability to identify SNPs in exome sequencing data was evaluated using data from the HapMap release 27 CEU population (ESM Figure 4). SNPs in the exome sequencing data were identified by calling sites with an allele frequency above 1%. Almost all HapMap SNPs with an allele frequency above 2% and about half of the SNPs with an allele frequency between 0.5% and 2% were identified in exome sequencing. Allele frequency estimates in the 90 unrelated European HapMap individuals have a high uncertainty, which is probably the main reason why we did not identify all HapMap variations with a MAF > 1%.

2.5.4. FALSE POSITIVE RATE OF SNP IDENTIFICATION

In the second stage genotyping project we selected 18,374 SNPs identified in the exome sequencing for genotyping. Of these 18,374 SNPs selected from the sequencing data, 1,110 did not replicate as SNPs in the genotyping effort. 180 of these SNPs are listed in dbSNPs and are probably the result of genotyping failures whereas the 930 remaining SNPs are probably false positive SNPs from the sequencing data. The high false positive rate (5.1%) is due to the fact that we did not apply any filters on the SNPs discovered in the exome sequencing (see ESM Methods section 2.6).

2.5.5. ESTIMATION OF THE PROBABILITY OF A SITE BEING POLYMORPHIC

We determined which sites were most likely polymorphic. This was performed by deriving ten different likelihoods for each site for each genotype using SOAPsnp [19] (see section 2.5.1). The major and minor allele was determined by selecting the two alleles with the highest counts in all individuals. Using the maximum likelihood of the allele frequency as a prior the probability of a site being polymorphic can be efficiently estimated assuming Hardy-Weinberg [18].

2.5.6. SELECTION OF SNPS SUITABLE FOR ASSOCIATION STUDIES

As a final call for reliable SNPs identified from 2,000 exome sequencing, we discarded all sites with a total depths below 1,000, an estimated allele frequency below 1% and a probability of being a variable site lower than 95%. 1,727,108 putative SNPs were found and of these sites 70,447 had a maximum likelihood allele frequency estimate higher than 1%. 70,219 sites also had a probability above 95% of being polymorphic. The 70,219 polymorphic sites were taken forward for annotation and subsequent association analysis. The frequency distribution is shown in for cases and controls in ESM Figure 8. Annotation was not successful for 37 SNPs because the number of coding bases was not a multiple of 3 bringing the number of SNPs to 70,182.

2.5.7. GENOTYPE CALLING

In some of the quality control analyses called genotypes were used instead of the genotype likelihoods, $p(X|G=g)$ where g is the number of minor alleles. The following procedure to call genotypes from the genotype likelihoods based on the sequencing data was applied. By using the aligned data we inferred the major and minor allele by choosing the two most frequently observed bases. Using the sequencing data the allele frequency, $\hat{f}$, was estimated using maximum likelihood (see section below). Assuming Hardy-Weinberg equilibrium we used the allele frequency as a prior in order to obtain a posterior probability of the genotypes

$$P\left(G=g\middle|X,\hat{f}\right)=\frac{p(X|G=g)p(G=g|\hat{f})}{\sum_{g'=0}^{2}p(X|G=g')p(G=g'|\hat{f})}$$

where

$$p\left(G=g\middle|\hat{f}\right)=\begin{cases}(1-\hat{f})^2 & if\ g=0\\ 2\hat{f}(1-\hat{f}) & if\ g=1\\ \hat{f}^2 & if\ g=2\end{cases}$$

Only a limited number of genotypes could accurately be called which is why most analysis are based directly on the genotype likelihoods.

2.5.8. ANNOTATION OF 70,219 SNPS USING THE SEATTLESNP ANNOTATOR

The SNPs were annotated using the SeattleSNP SNP annotator (http://gvs.gs.washington.edu/SeattleSeqAnnotation/). Multiple gene isoforms were merged, and the SNPs were annotated according to the most deleterious function type. The non-redundant table where a site was only included once. ESM Table 5 shows the non-redundant annotation where a SNP was included only once in the most deleterious functional category and only annotated to one gene. The row order of the functional types also shows the ranking of the different deleterious categories for SNPs. In the annotation tables the SNPs were separated into four additional categories to show whether the SNP is also in dbSNP 129 exclusively, in the 1000 Genomes Project (release of April 2009) exclusively, in both dbSNP and the 1000 Genomes Project or if the SNP is novel. Note that 37 SNPs could not be annotated because the number of coding bases was not a multiple of 3.

2.6. SNP filters for association

Before performing association analysis on the sequencing data we chose to include multiple stringent filters. This was done in order to remove SNPs that either were likely to be errors or SNPs that showed bias which could be correlated with case-control status. Filters based on the base quality scores and based on biases observed in sequencing time were applied.

2.6.1. QUALITY SCORE DISTRIBUTION (RST)

For each polymorphic site we tested for differences in the quality score distribution between the major and minor allele. SNPs were removed if the scores of the minor allele were significantly lower than for the major allele (Wilcoxon rank sum test, $P < 0.01$).

2.6.2. REPEATEDLY ALIGNED READS (REP)

We also tested whether the minor allele was enriched in repeatedly aligned reads. The repeatedly aligned reads were reads which map equally well to multiple locations on the genome. SNPs were removed if the minor allele was significantly enriched in the repeatedly aligned reads (Fisher's exact test, $P < 0.01$).

2.6.3. READ POSITIONS (EDGE)

The position of the bases in the reads covering a site should be random. We tested if this was the case and removed SNPs if the minor allele was significantly enriched within 10 bps of 5’ or 3’ ends of the reads (Fisher’s exact test, $P < 0.01$).

2.6.4. CLUSTERING IN SEQUENCING TIME (WAITING TIMES)

Three methods for testing for bias in the sequencing were applied. The first was based on waiting times between observing an allele. With N individuals we assigned each individuals to their rank in sequencing time s such that $s_i \in \{1,2,\ldots,\mathrm{N}\}$ is the rank of individual i. Let $a_i \in \{0,1\}$ denote whether an individual has the allele ($a_i = 1$) or not ($a_i = 0$). A distance between to individuals i and j was defined as

$$d(i,j) = \begin{cases} s_i - s_j & if\ s_i - s_j < N/2 \\ N - s_i + s_j & else \end{cases}$$

and using

$$\sum_{i=1}^{N}\sum_{j=1}^{N} d(i,j) I(a_i = a_j)$$

as a test statistic. A *P*-value was obtained by permutations.

2.6.5. CLUSTERING IN SEQUENCING TIME (WILLCOX)

In the second approach we tested if the presence of alleles was random based on the rank of the sequencing time. Here, a Mann-Whitney test was applied separately within cases and controls (threshold: $P < 0.01$).

2.6.6. CLUSTERING IN SEQUENCING TIME (KULLDORFF)

In the third approach we investigated whether the rare allele was observed more frequently in defined windows of sequencing time compared with the remaining parts of the data. This was done using a one-dimensional scan statistics [21] with a region size ranging from 20 to 500 base pairs. A *P*-value was obtained using 1,000 permutations (threshold: $P < 0.01$).

2.7. Association testing

As stated, exome sequencing was performed in 1,000 type 2 diabetes patients enriched for cardio-metabolic risk phenotypes (cases) and 1,000 glucose-tolerant, metabolically healthy individuals (controls) (ESM Table 2). In ESM Figure 3 the number of individuals has been plotted as a function of mean depth in controls and cases. It is obvious that the depth in controls and cases differed with an on average higher depth in controls compared to cases. In addition, it is noteworthy that few individuals had an average depth lower than 4. Before removing the low quality reads all individuals had a depth of at least 8. Thus, data from some individuals were of much lower quality than data from others. Therefore, we could not perform association studies on called genotypes since this would lead to different genotype accuracy in the cases and controls which would lead to spurious associations. Therefore, we chose to perform the association testing directly on the genotype likelihoods.

2.7.1. STATISTICAL METHOD FOR SEQUENCING-BASED ASSOCIATION ANALYSIS

For the 1,000 cases and 1,000 controls we decided to base the association on the reads and not on the genotypes. The testing was based on the frequency estimator (see section 2.5) where a likelihood was obtained for a given sample. Here we obtained a likelihood for the entire sample and a likelihood for the cases and controls, separately. These likelihoods were used to construct a likelihood ratio test that tested for differences in allele frequencies between the cases and control. The applied method has been reported by Kim *et al* [20].

2.7.2. RESULTS OF ASSOCIATION TESTING IN STAGE 1

The patterns of association showed a large bias that caused a considerable inflation of associated SNPs. The bias was markedly reduced when the low depth sites and when sites with adjacent markers were excluded. The explanation for the large bias is that for some of the sites the presence of alleles varies in sequencing time. Thus, for some sites the allele was only present in early stages of sequencing while largely absent in later sequencing. An example of such a site is shown in ESM Figure 6 where the presence of alleles was sorted according to sequencing time. For this site the rare allele was largely absent in the first 800 individuals while it was present in most other individuals. Because the individual samples were not completely randomized this procedure gave rise to false association signals.

For all SNPs we investigated the presence of this bias as a function of sequencing timeline applying three approaches (see section 2.6.4). All the tests were performed within cases and controls, respectively. After removing SNPs with alleles that clustered in sequencing time the majority of the bias was removed (ESM Figure 9). We also added an additional filtering based on genotypes such that at least 15 of the genotypes within cases and within controls had a probability of at least 95% and at least 15 of the minor alleles within cases and within controls were called.

3. Stage 2: Genotyping and association mapping in of 16,192 coding variants in 15,989 individuals

3.1. SNP selection from stage 1 for Illumina iSelect genotyping in 16,988 Danish individuals

Based on the results of exome sequencing in 2,000 individuals, we selected 20,005 SNPs for genotyping on a customized Illumina iSelect genotyping array. SNPs were selected based on four criteria:

1) 1,804 SNPs nominally associated ($P < 0.05$) with disease status in the stage 1 sequencing-based association study. SNPs showing clustering in sequencing time were excluded from this selection.
2) SNPs belonging to the annotation categories presumed to have the highest probability of a functional effect: Nonsense, nonsynonymous, splice site variants and variants in untranslated regions (UTR). 18,358 SNPs which could be designed and manufactured for Illumina iSelect were selected in this approach divided on 179 nonsense, 15,789 nonsynonymous variants, 219 SNPs located in splice sites and 2,171 SNPs in the UTR. Of SNPs selected from this approach, 756 SNPs were already selected based on nominal association in stage 1.
3) For 192 loci previously associated with metabolic traits (type 2 diabetes, fasting plasma glucose, plasma glucose 2 hrs post OGTT, obesity, BMI, waist circumference, blood pressure, hypertension and cardiovascular disease) we furthermore selected all synonymous variants found in stage 1 exome sequencing. The regions for which these SNPs were selected, were defined based on LD ($D' > 0.8$) with the reported lead SNP based on HapMap CEU release 27 data. 599 SNPs were selected by this approach.

3.2. Illumina iSelect genotyping

3.2.1. LABORATORY METHODS

We genotyped 16,988 samples passing sample handling quality-control metrics at the Beijing Genomics Institute using a custom-designed iSelect assay from Illumina. Two methods of quality-control were performed including Nanodrop 1000 and gel electrophoresis. Before genotyping, all samples were randomly selected and assigned to well positions to avoid possible bias among different batches. Genotyping calling was done with normalized intensities using the Illumina clustering algorithm (Gentrain 1.0) implemented in GenomeStudio

Genotyping Analysis Module (v1.6.3). To generate a custom cluster file of this project, we used 1,032 samples selected from different batches, including 172 samples with stage 1 exome sequencing results. After exclusion of 32 samples with call rate below 95% and six samples with low concordance between genotyping and sequencing, 994 samples were used to perform reclustering. Due to either manufacturing problems at Illumina or the SNPs exhibited poor performances in the laboratory, a total of 1,262 SNPs failed to provide reliable genotype results, leaving 18,744 SNPs with robust genotypes. We excluded a SNP with ambiguous cluster or if any of the following criteria was fulfilled: no genotype was called for any samples, SNP call rate < 0.95, concordance between genotyping and sequencing < 90%, cluster separation metric < 0.4, AB R mean (the mean normalized intensity of the heterozygote cluster) < 0.2, AB T mean (the mean of the normalized theta values of the heterozygote cluster) ranging from 0-0.2 and 1-0.8, heterozygosity excess less than -0.3 or greater than 0.3, for X chromosome SNPs male heterozygosity > 0.3%. All genotypes for those SNPs were not included in final report. This final cluster file was then used to call genotypes for all attempted project samples.

3.2.2. ILLUMINA ISELECT GENOTYPING DATA QUALITY CONTROL AND CLEANING

Genotyping data was produced for 18,744 SNPs in 16,988 individuals from five different Danish study populations (ESM Table 1). The quality control of the SNPs and individuals were performed to ensure that our samples were homogeneous, the individuals were independent and the genotypes were reliable. Analyses were performed using the PLINK software (version 1.07) [22].

Quality control of individuals and samples

The filtering of individuals was initiated by discarding all samples with a genotype call rate less than 95.0%. The average sample call rate was 99.9% including all SNPs. Also, the relatedness between individuals was estimated by using pair wise identity-by-descent (IBD) analysis. If pairs of individuals showed third-degree relationship or closer only one sample (the one with highest call rate) was kept for further analysis. The inbreeding coefficient was estimated based on the observed versus expected number of homozygous genotypes in the given sample. Genotypes for individuals, with an inbreeding coefficient greater than 0.1 or less than -0.1, were discarded. The inbreeding coefficient on the X-chromosome was estimated to determine the sex of the individual (women: inbreeding coefficient rate above 0.5, men: inbreeding coefficient below 0.8). We used these lenient thresholds due to the low number of X-chromosome SNPs. The sample was discarded for further analysis if the genetically inferred sex did not match the reported sex. For some of the individuals we already had genotyping results that overlapped with the present study. Using these SNPs we confirmed that the genotypes matched earlier records which insured that samples had not been mislabelled or switched. In total, 999 individuals failed one or more of the above quality criteria and were excluded. Relatedness was the major reason for the large failure proportion. The final dataset which was taken forward for association analysis included a total of 15,989 individuals.

Quality control and filtering of SNPs

The filtering of SNPs included discarding SNPs with a MAF below 0.5% and SNPs with a genotype call rate < 95.0%. Hardy-Weinberg equilibrium *P*-value was calculated including all samples (n = 15,989) and SNPs with a *P*-value < 10^{-7} were discarded. Some sites on the genome may be problematic to genotype due to cross hybridization of the primers. Especially, if the cross hybridization appears on the X-chromosome as this will cause spurious association signals for phenotypes associated with sex. Therefore we tested all autosomal SNPs for association with sex and SNPs showing $P < 10^{-6}$ were excluded from phenotype analysis. 2,552 SNPs were excluded according to the quality criteria and the final dataset for analysis included 16,192 SNPs.

3.3. Statistical analysis of stage 2 data

A total of 16,192 SNPs with a MAF > 0.5% were tested in up to 15,989 individuals for associations with metabolic phenotypes using linear regression for quantitative phenotypes (BMI, waist circumference, fasting levels of plasma glucose, serum insulin, serum cholesterol, serum HDL-cholesterol, and serum triacylglycerol and systolic and diastolic blood pressure) and logistic regression for binary variables (type 2 diabetes, obesity and hypertension) assuming an additive (or log-additive) genetic model. To adjust for any stratification bias, principal component analysis was performed using the covariance matrix of all 16,192 SNPs passing quality control [23, 24]. Using logistic and linear regression we tested whether the principal components were associated with the phenotypes. *P*-values for association of the first principal component with type 2 diabetes, obesity and hypertension were 0.37, 0.20 and 0.14, respectively. For the quantitative phenotypes none were significant after Bonferroni correction for the number of phenotypes. Similarly, when applying the tests to the first nine principal components none of the associations with phenotypes were close to the Bonferroni corrected *P*-value threshold.

The first principal component as well as sex was included in the model as covariates. All quantitative traits were rank normalized to a normal distribution before analysis. Inflation factors (λ) which were estimated for all association analyses were acceptable (range 1.002-1.086, ESM Figure 7). *P*-values were subsequently corrected by genomic control and these *P*-values are presented in all stage 2 data (Figure 2 and ESM Figure 7).

3.4. Results of stage 2 genotyping and association studies

3.4.1. SELECTION OF KNOWN ASSOCIATED SNPS

Based on the known lead SNPs we defined known associated variants for a trait as variations that are either lead SNPs for that phenotype or in LD ($r^2 > 0.2$) with a lead SNP for that phenotype. We used phased data from the 1000 Genomes (release 20110521) to estimate the LD. Approximately 600 SNPs were not present in the 1000 Genomes data. We denoted those SNPs as known if they were within 1 Mb of a lead SNP for that phenotype.

3.4.2. RESULTS OF THE SNPS ASSOCIATED IN STAGE 1

1,804 SNPs were selected based on their association in stage 1 including 756 SNPs that were also selected based on their functional annotation. Of these SNPs about 1,600 also passed quality control of stage 2 data. For three of the major metabolic phenotypes, that defined the case-control status of the stage 1 individuals, we analyzed the association with the 1,600 SNPs in stage 2. The distributions of the association signals for these SNPs are shown in ESM Figure 10. We also show the results after removing SNPs known to be associated with the trait under investigation. These SNPs were selected based on all genome-wide significant associations found in the literature and the SNPs in LD with them. To estimate LD we used the 1000 Genomes imputed data for the central Europeans (CEU) and selected SNPs if they were in moderate or high LD ($r^2 > 0.2$). SNPs not found in the 1000 Genomes were denoted as known if they were closer than 1 MB of a known associated variation. For type 2 diabetes we found a clear excess of low *P*-values meaning that this set of SNPs was enriched for type 2 diabetes associated variations. To further analyze these results we estimated the false discovery rates and fitted a mixture model with two components using fdrtool [25]. One component represents the sites that are not associated with the trait and the other component represents the interesting SNPs that are associated with the trait. The mixture proportion for the SNPs that are not associated, eta0, can be interpreted as an estimate of the fraction of non-associated SNPs. Thus, an estimated eta0 of 1 means that we did not find evidence of presents of associated SNPs. ESM Figure 11, ESM Figure 12 and ESM Figure 13 show the top associations of these SNPs and the distribution of the interesting SNPs. Especially, type 2 diabetes has a high estimated proportion of associated SNPs.

3.4.3. RESULTS OF OVERALL ASSOCIATION ANALYSIS IN STAGE 2

After the stage 2 analyses of SNPs associated with the combined phenotype in stage 1, the remaining analyses of stage 2 genotyping data were performed in all 15,989 individuals including the 2,000 individuals who were part of the exome sequencing in stage 1. Overall results of these analyses for 12 metabolic phenotypes are shown in ESM Figure 7 and Figure 2, while more detailed information on highest ranking associations is shown in ESM Table 7.

4. Stage 3: Validation of selected tentative associations in up to 63,896 European individuals

4.1. Selection of SNPs for validation in stage 3 genotyping

To follow up on the most promising associations from stage 2 association analysis we selected top hits for 12 different metabolic traits. Three binary (type 2 diabetes, obesity [assessed as BMI > 30 kg/m^2 vs. BMI < 25 kg/m^{-2}] and hypertension) and nine quantitative traits (BMI, waist circumference, fasting levels of plasma glucose, serum insulin, serum cholesterol, serum HDL-cholesterol and serum triacylglycerol and systolic and diastolic blood pressure) were considered. SNPs in LD ($r^2 > 0.2$) with a known genome-wide significant ($P < 5 \times 10^{-8}$) associated lead SNP for the given metabolic trait were also excluded. The LD measurements were determined using the genotype data from the present study as well as 1000 Genomes data (release 2011021) (section 3.4.1). For the lipid traits we additionally defined a known associated locus as the region spanning 250 Kb up- and downstream of the known associated genome-wide significant lead SNP. All SNPs within these regions were excluded for follow-up.

The selection of SNPs was based on the most associated SNPs in terms of lowest *P*-value. All SNPs showing an association below Bonferroni corrected significance were selected for replication. For all other SNPs we attempted to exclude SNPs that were part of previous large scale association studies as well as their proxies ($r^2 > 0.8$). This was performed by excluding SNPs that were on commonly used GWAS arrays as well as SNPs in LD ($r^2 > 0.8$) with SNPs on the arrays. The arrays considered in this aspect were: Illumina Human Hap 300, 550 and 660 bead arrays and Affymetrix GeneChip Human Mapping 500K array. Application of these exclusion criteria yielded a final list consisting of 45 non-redundant SNPs tentatively associated with one or more of the 12 metabolic traits.

4.2. Stage 3 statistical analysis

45 SNPs covering 51 associations were selected for stage 3 replication. For each SNP we estimated the combined effect size on the metabolic traits by inclusion in a fixed-effects meta-analysis using the METAL software [26] (http://www.sph.umich.edu/csg/abecasis/Metal/). For SNPs associated with a quantitative trait (including BMI (kg/m^2), waist circumference (cm), fasting plasma glucose, fasting serum insulin, serum total cholesterol, serum HDL-cholesterol, fasting serum triacylglycerol, systolic and diastolic blood pressure) an overall z-statistics relative to each reference allele was estimated based on *P*-value and direction of effect adjusted for the number of individuals in each sample. As for dichotomized traits (type 2 diabetes, obesity an hypertension) the odds ratio (OR) and standard error (SE) were used. Here, each effect size was weighted according to the estimated standard errors by using the inverse corresponding standard error [26]. An overview of replication efforts for individual SNPs in stage 3 is shown in ESM Table 6.

5. Gene expression profiling

5.1. Tissue RNA samples

Human tissue mRNA samples pooled from donors or suddenly deceased individuals of Caucasian or Asian ancestry were obtained commercially (ClonTech Laboratories Inc., Mountain View, CA): Human Aorta Poly A+ RNA (cat. no. 636153), Human Blood, Peripheral Leukocytes Poly A+ RNA (cat. no. 636170), Human Brain Poly A+ RNA (cat. no. 636102), Human Colon Poly A+ RNA (cat. no. 636146), Human Small Intestine Poly A+ RNA (cat. no. 636125), Human Adipose Tissue Poly A+ RNA (cat. no. 636162), Human Kidney Poly A+ RNA (cat. no. 636118), Human Liver Poly A+ RNA (cat. no. 636101), Human Pancreas Poly A+ RNA (cat. no. 636119), Human Skeletal Muscle Poly A+ RNA (cat. no. 636120), Human Placenta Poly A+ RNA (cat. no. 636103).

5.2. cDNA synthesis

Complementary DNA (cDNA) was synthesized from 100 ng mRNA or 1 µg total RNA template using the QuantiTect Reverse Transcription Kit (Qiagen, Hilden, Germany) according to the manufacturers recommendation. The cDNA was diluted in 50 mL H_2O.

5.3. Real-time PCR

CD300LG, *COBLL1*, *MACF1*, *ACP1*, *ZFAND2B*, *GPSM1*, *PRRC2A* and *GRB14* mRNA was quantified in duplicate samples by real-time PCR on an ABI Prism 7900 HT system (Applied Biosystems, Foster City, CA) using TaqMan gene expression assays for *CD300LG* (Hs00926077_m1), *COBLL1* (Hs00383292_m1), *MACF1* (Hs00201478_m1), *ACP1* (Hs00962877_m1), *ZFAND2B* (Hs00373414_m1), *GPSM1* (Hs00404578_m1), *PRRC2A* (Hs00190347_m1) and *GRB14* (Hs00182949_m1). The mRNA quantities of target genes were normalized to the mRNA level of *PPIA* (cyclophilin A, 4326316E) and expressed in arbitrary units (AU). The reactions were performed with 10 ng of cDNA in 4.5 µL of water, 0.5 µL of gene expression assay, and 5 µL of Universal PCR Master Mix (Applied Biosystems).

6. URLs

Plink http://pngu.mgh.harvard.edu/~purcell/plink/
Soap http://soap.genomics.org.cn
Metal http://www.sph.umich.edu/csg/abecasis/Metal/
R http://www.r-project.org/
Angsd http://www.popgen.dk/albrecht/software/dirty/
Seattle SNP annotation http://gvs.gs.washington.edu/SeattleSeqAnnotation/

7. References

1. Jørgensen T, Borch-Johnsen K, Thomsen TF, Ibsen H, Glumer C, Pisinger C (2003) A randomized non-pharmacological intervention study for prevention of ischaemic heart disease: Baseline results Inter99 (1). Eur J Cardiovasc Prev Rehab 10:377-386
2. Boesgaard TW, Grarup N, Jørgensen T et al (2010) Variants at DGKB/TMEM195, ADRA2A, GLIS3 and C2CD4B loci are associated with reduced glucose-stimulated beta cell function in middle-aged Danish people. Diabetologia 53:1647-1655
3. Glümer C, Jørgensen T, Borch-Johnsen K (2003) Prevalences of diabetes and impaired glucose regulation in a Danish population: the Inter99 study. Diabetes Care 26:2335-2340
4. Thyssen JP, Linneberg A, Menne T, Nielsen NH, Johansen JD (2009) The prevalence and morbidity of sensitization to fragrance mix I in the general population. Br J Dermatol 161:95-101

5. World Health Organization Study Group (1999) Definition, Diagnosis and Classification of Diabetes Mellitus and its Complications. Part 1: Diagnosis and Classification of Diabetes Mellitus. Tech. Rep. Ser. WHO/NCD/NCS/99.2 edn. World Health Organization, Geneva,
6. Lauritzen T, Griffin S, Borch-Johnsen K et al (2000) The ADDITION study: proposed trial of the cost-effectiveness of an intensive multifactorial intervention on morbidity and mortality among people with Type 2 diabetes detected by screening. Int J Obes Relat Metab Disord 24 (Suppl 3):S6-11
7. American Diabetes Association (2004) Diagnosis and Classification of Diabetes Mellitus. Diabetes Care 27:s5-s10
8. Balkau B, Eschwege E, Tichet J, Marre M (1997) Proposed criteria for the diagnosis of diabetes: evidence from a French epidemiological study (D.E.S.I.R.). Diabetes Metab 23:428-434
9. Meyre D, Delplanque J, Chèvre JC et al (2009) Genome-wide association study for early-onset and morbid adult obesity identifies three new risk loci in European populations. Nat Genet 41:157-159
10. Sladek R, Rocheleau G, Rung J et al (2007) A genome-wide association study identifies novel risk loci for type 2 diabetes. Nature 445:881-885
11. Berglund G, Elmstahl S, Janzon L, Larsson SA (1993) The Malmo Diet and Cancer Study. Design and feasibility. J Intern Med 233:45-51
12. Enhörning S, Wang TJ, Nilsson PM et al (2010) Plasma copeptin and the risk of diabetes mellitus. Circulation 121:2102-2108
13. Isomaa B, Forsen B, Lahti K et al (2010) A family history of diabetes is associated with reduced physical fitness in the Prevalence, Prediction and Prevention of Diabetes (PPP)-Botnia study. Diabetologia 53:1709-1713
14. van 't Riet E, Alssema M, Rijkelijkhuizen JM, Kostense PJ, Nijpels G, Dekker JM (2010) Relationship between A1C and glucose levels in the general Dutch population: the new Hoorn study. Diabetes Care 33:61-66
15. Reiling E, van 't Riet E, Groenewoud MJ et al (2009) Combined effects of single-nucleotide polymorphisms in GCK, GCKR, G6PC2 and MTNR1B on fasting plasma glucose and type 2 diabetes risk. Diabetologia 52:1866-1870
16. Zavrelova H, Hoekstra T, Alssema M et al (2011) Progression and regression: distinct developmental patterns of diabetic retinopathy in patients with type 2 diabetes treated in the diabetes care system west-friesland, the Netherlands. Diabetes Care 34:867-872
17. Welschen LM, van OP, Dekker JM, Bouter LM, Stalman WA, Nijpels G (2007) The effectiveness of adding cognitive behavioural therapy aimed at changing lifestyle to managed diabetes care for patients with type 2 diabetes: design of a randomised controlled trial. BMC Public Health 7:74
18. Li Y, Vinckenbosch N, Tian G et al (2010) Resequencing of 200 human exomes identifies an excess of low-frequency non-synonymous coding variants. Nat Genet 42:969-972
19. Li R, Li Y, Fang X et al (2009) SNP detection for massively parallel whole-genome resequencing. Genome Res 19:1124-1132
20. Kim SY, Lohmueller KE, Albrechtsen A et al (2011) Estimation of allele frequency and association mapping using next-generation sequencing data. BMC Bioinformatics 12:231
21. Kulldorff M, Nagarwalla N (1995) Spatial disease clusters: detection and inference. Stat Med 14:799-810
22. Purcell S, Neale B, Todd-Brown K et al (2007) PLINK: a tool set for whole-genome association and population-based linkage analyses. Am J Hum Genet 81:559-575
23. Patterson N, Price AL, Reich D (2006) Population structure and eigenanalysis. PLoS Genet 2:e190
24. Price AL, Patterson NJ, Plenge RM, Weinblatt ME, Shadick NA, Reich D (2006) Principal components analysis corrects for stratification in genome-wide association studies. Nat Genet 38:904-909
25. Strimmer K (2008) fdrtool: a versatile R package for estimating local and tail area-based false discovery rates. Bioinformatics 24:1461-1462
26. Willer CJ, Li Y, Abecasis GR (2010) METAL: fast and efficient meta-analysis of genomewide association scans. Bioinformatics 26:2190-2191
27. Voight BF, Scott LJ, Steinthorsdottir V et al (2010) Twelve type 2 diabetes susceptibility loci identified through large-scale association analysis. Nat Genet 42:579-589
28. Speliotes EK, Willer CJ, Berndt SI et al (2010) Association analyses of 249,796 individuals reveal 18 new loci associated with body mass index. Nat Genet 42:937-948
29. Dupuis J, Langenberg C, Prokopenko I et al (2010) New genetic loci implicated in fasting glucose homeostasis and their impact on type 2 diabetes risk. Nat Genet 42:105-116
30. Hansen T, Drivsholm T, Urhammer SA et al (2007) The BIGTT test: a novel test for simultaneous measurement of pancreatic β-cell function, insulin sensitivity, and glucose tolerance. Diabetes Care 30:257-262
31. Matthews DR, Hosker JP, Rudenski AS, Naylor BA, Treacher DF, Turner RC (1985) Homeostasis model assessment: insulin resistance and beta-cell function from fasting plasma glucose and insulin concentrations in man. Diabetologia 28:412-419
32. Matsuda M, DeFronzo RA (1999) Insulin sensitivity indices obtained from oral glucose tolerance testing: comparison with the euglycemic insulin clamp. Diabetes Care 22:1462-1470
