# Supplementary material for: Exome sequencing-driven discovery of coding polymorphisms associated with common metabolic phenotypes
Source: Diabetologia. 2012 Nov 19;56(2):298–310. doi: 10.1007/s00125-012-2756-1 (PMC3536959; doi:10.1007/s00125-012-2756-1)
Supplement: Supplementary file 3 — (PDF 200 kb) [file 125_2012_2756_MOESM3_ESM.pdf]

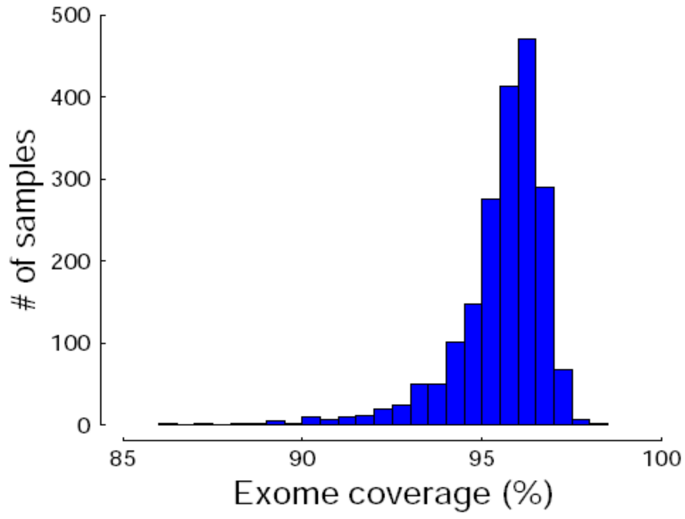

ESM Figure 1 Distribution of exome target region coverage in the 2,000 Danish samples.

The distribution of exome coverage was plotted using the effective reads before filtering poor quality reads. The exome coverage was defined as the number of bases (%) covered by at least one uniquely mapped read for each base and the median coverage per individual was calculated to 96%.
