# Supplementary material for: Exome sequencing-driven discovery of coding polymorphisms associated with common metabolic phenotypes
Source: Diabetologia. 2012 Nov 19;56(2):298–310. doi: 10.1007/s00125-012-2756-1 (PMC3536959; doi:10.1007/s00125-012-2756-1)
Supplement: Supplementary file 4 — (PDF 263 kb) [file 125_2012_2756_MOESM4_ESM.pdf]

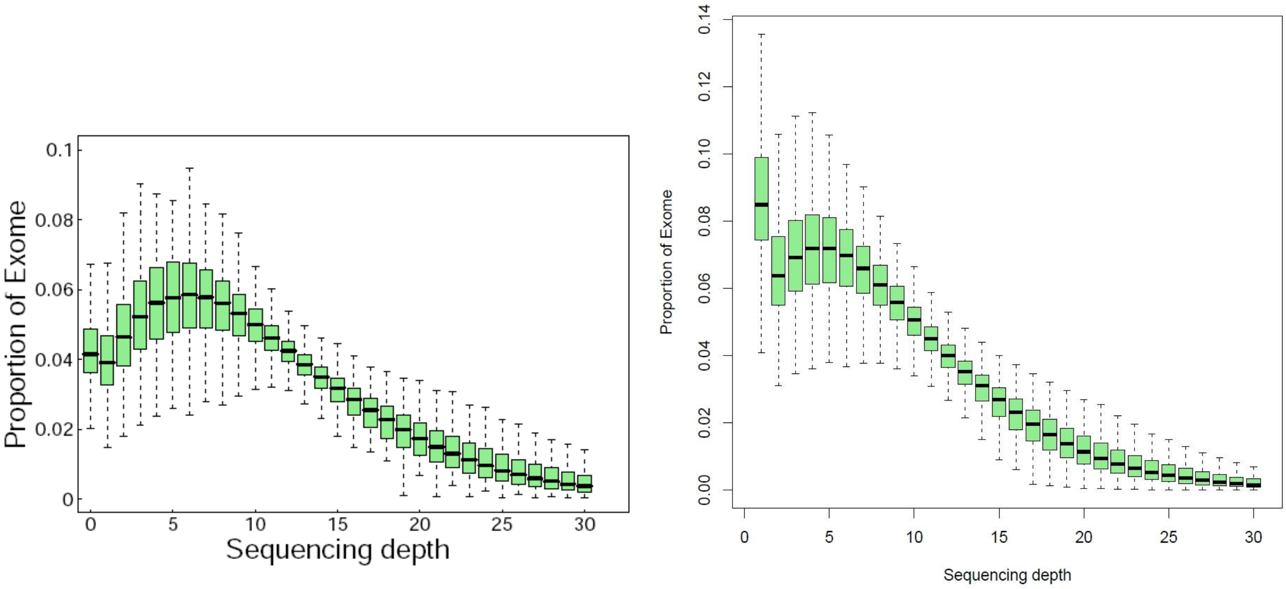

A

B

ESM Figure 2 The distribution of per-base sequencing depth in 2,000 samples.

The proportion of the exome target region that was covered by 0 – 30 read bases was calculated for each sample. For each per-base sequencing depth, the median proportion (shown as the black bar) in the 2,000 samples was calculated. In the left plot all sites were applied whereas in the right plot we excluded all low quality (<Q20) sites as well as sites where reads were not unique (i.e. the reads had been mapped to more than one site during the alignment). Each box plot shows the lower and upper quartile boundary. The whiskers are placed 1.5 times the interquartile.
