# Supplementary material for: Exome sequencing-driven discovery of coding polymorphisms associated with common metabolic phenotypes
Source: Diabetologia. 2012 Nov 19;56(2):298–310. doi: 10.1007/s00125-012-2756-1 (PMC3536959; doi:10.1007/s00125-012-2756-1)
Supplement: Supplementary file 5 — (PDF 223 kb) [file 125_2012_2756_MOESM5_ESM.pdf]

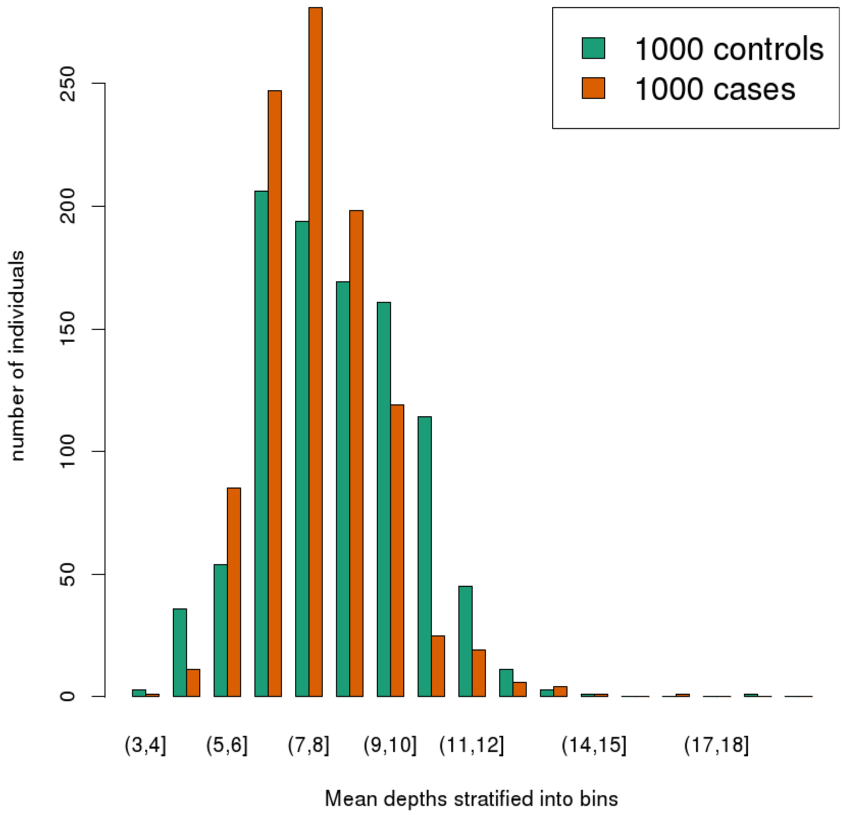

ESM Figure 3 **The average depths distribution in 1,000 cases and 1,000 controls after removal of low quality bases.**
