# Supplementary material for: Exome sequencing-driven discovery of coding polymorphisms associated with common metabolic phenotypes
Source: Diabetologia. 2012 Nov 19;56(2):298–310. doi: 10.1007/s00125-012-2756-1 (PMC3536959; doi:10.1007/s00125-012-2756-1)
Supplement: Supplementary file 6 — (PDF 228 kb) [file 125_2012_2756_MOESM6_ESM.pdf]

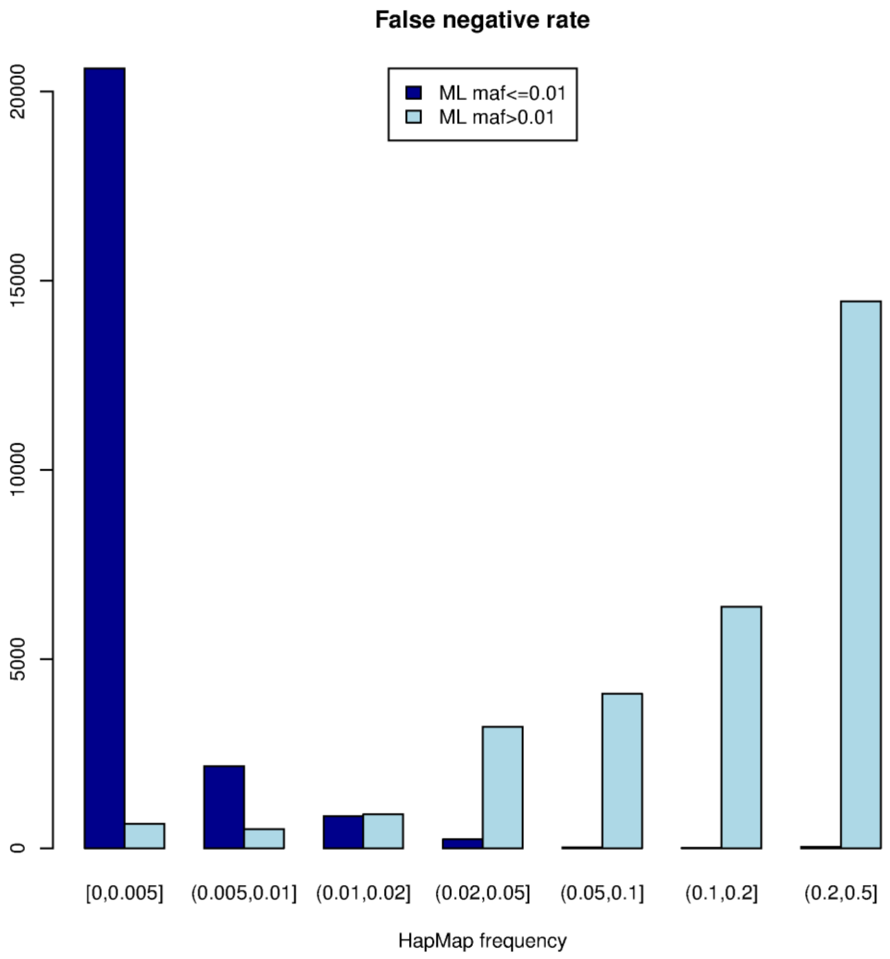

ESM Figure 4 Number of SNPs identified in exome sequencing of 2,000 individuals binned according to their frequencies in HapMap.

In the graph the light blue bars are the SNPs that were inferred in LuCamp while the dark blue are the sites that were not identified in exome sequencing of 2,000 samples. In this figure we called SNPs by calling all sites with an allele frequency above 0.5%. The first bin contains the sites that were genotyped but not polymorphic in HapMap.
