# Supplementary material for: Exome sequencing-driven discovery of coding polymorphisms associated with common metabolic phenotypes
Source: Diabetologia. 2012 Nov 19;56(2):298–310. doi: 10.1007/s00125-012-2756-1 (PMC3536959; doi:10.1007/s00125-012-2756-1)
Supplement: Supplementary file 7 — (PDF 339 kb) [file 125_2012_2756_MOESM7_ESM.pdf]

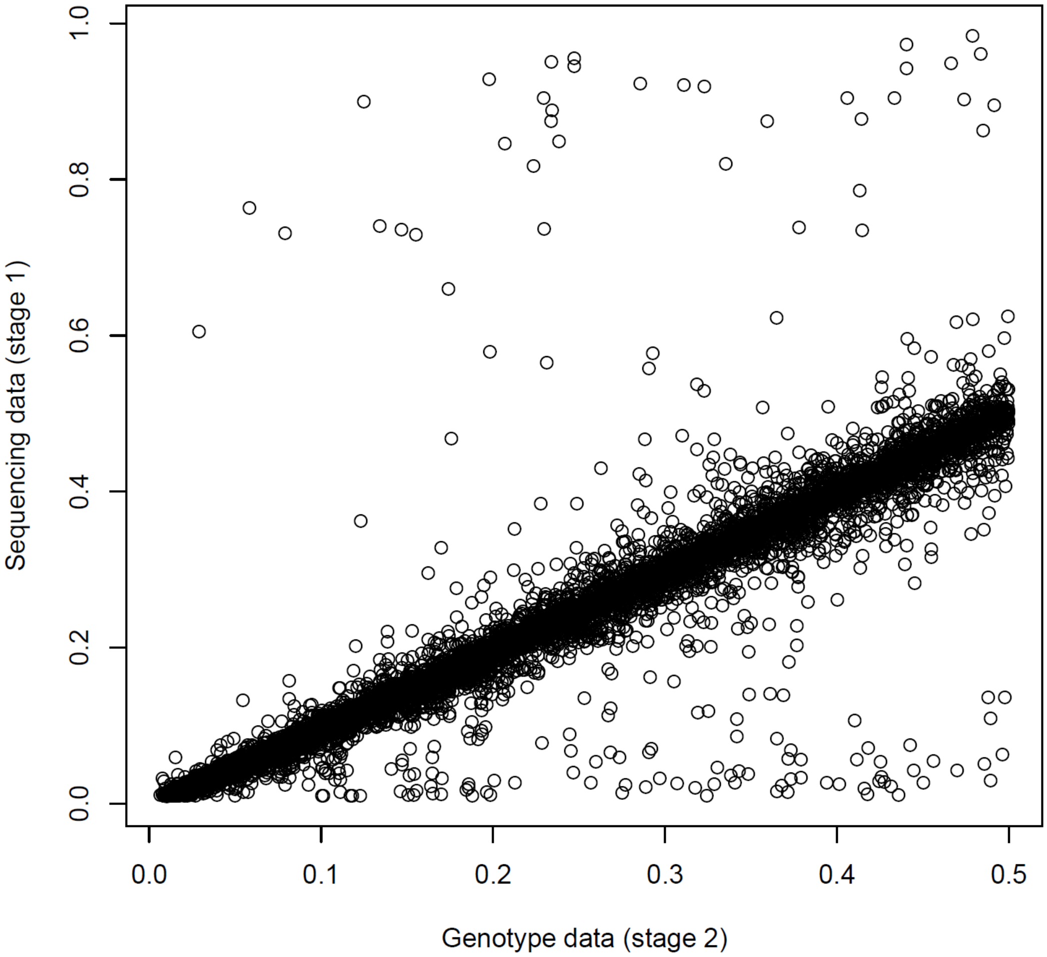

ESM Figure 5 Comparison of allele frequencies from exome sequencing with chip genotyping

Allele frequencies from exome sequencing (stage 1) compared with allele frequencies for the same SNPs and same individuals in genotyping data (stage 2). Only few outliers are observed suggesting accurate allele frequency estimation in exome sequencing data.
