# Supplementary material for: Exome sequencing-driven discovery of coding polymorphisms associated with common metabolic phenotypes
Source: Diabetologia. 2012 Nov 19;56(2):298–310. doi: 10.1007/s00125-012-2756-1 (PMC3536959; doi:10.1007/s00125-012-2756-1)
Supplement: Supplementary file 8 — (PDF 224 kb) [file 125_2012_2756_MOESM8_ESM.pdf]

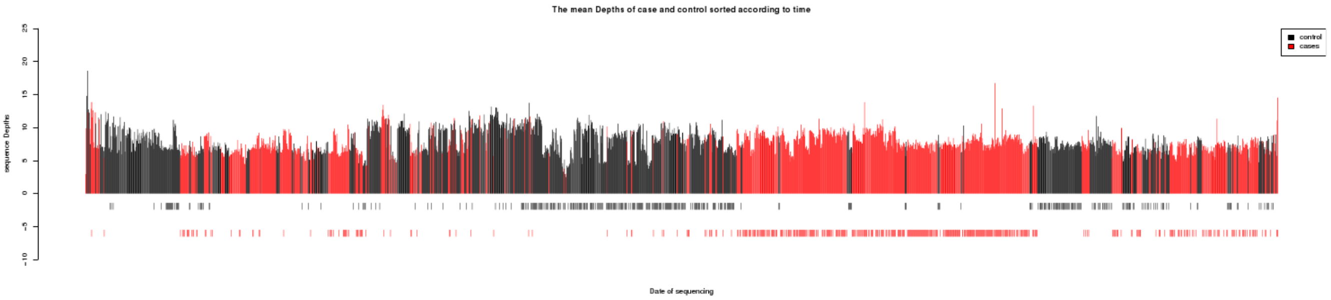

ESM Figure 6 Mean sequencing depth of cases and controls sorted according to chronological time of sequencing.

The horizontal axis is the time of sequencing and the vertical axis is the sequencing depth. Each bar is one individual (Red: cases, Black: controls). If the rare allele is detected in an individual a mark has been added under the plot (red: rare allele in cases, black: rare allele in controls). It is seen that the time of sequencing is correlated with the identification of the alleles. In the beginning (from left) the rare allele is not present as much as in the middle of the plot.
