# Supplementary material for: Exome sequencing-driven discovery of coding polymorphisms associated with common metabolic phenotypes
Source: Diabetologia. 2012 Nov 19;56(2):298–310. doi: 10.1007/s00125-012-2756-1 (PMC3536959; doi:10.1007/s00125-012-2756-1)
Supplement: Supplementary file 9 — (PDF 474 kb) [file 125_2012_2756_MOESM9_ESM.pdf]

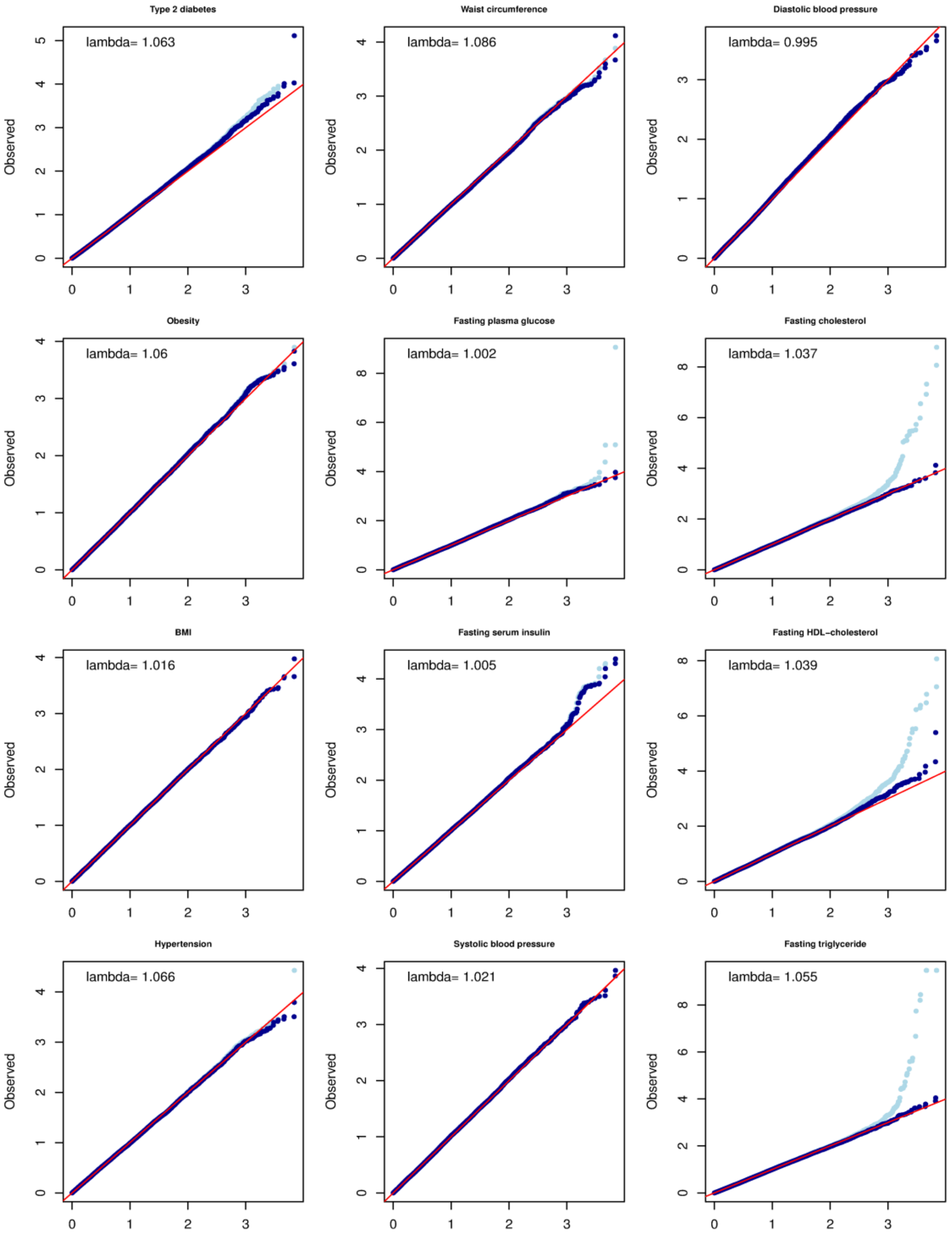

ESM Figure 7 Quantile-quantile plots of 16,192 SNPs for 12 metabolic traits in up to 15,989 Danish individuals (stage 2).

For each of the traits the observed T statistics were plotted against the expected T statistics under the null hypothesis. In light blue colours are all SNPs passing quality control. Dark blue dots show results where SNPs that have been established as known genome-wide associated signals for each trait have been removed. All *P*-values were corrected by genomic control. The inflation factors (λ) are shown for each analysis and were estimated by dividing observed median statistics by the expected median statistics.
