# Supplementary material for: Exome sequencing-driven discovery of coding polymorphisms associated with common metabolic phenotypes
Source: Diabetologia. 2012 Nov 19;56(2):298–310. doi: 10.1007/s00125-012-2756-1 (PMC3536959; doi:10.1007/s00125-012-2756-1)
Supplement: Supplementary file 10 — (PDF 241 kb) [file 125_2012_2756_MOESM10_ESM.pdf]

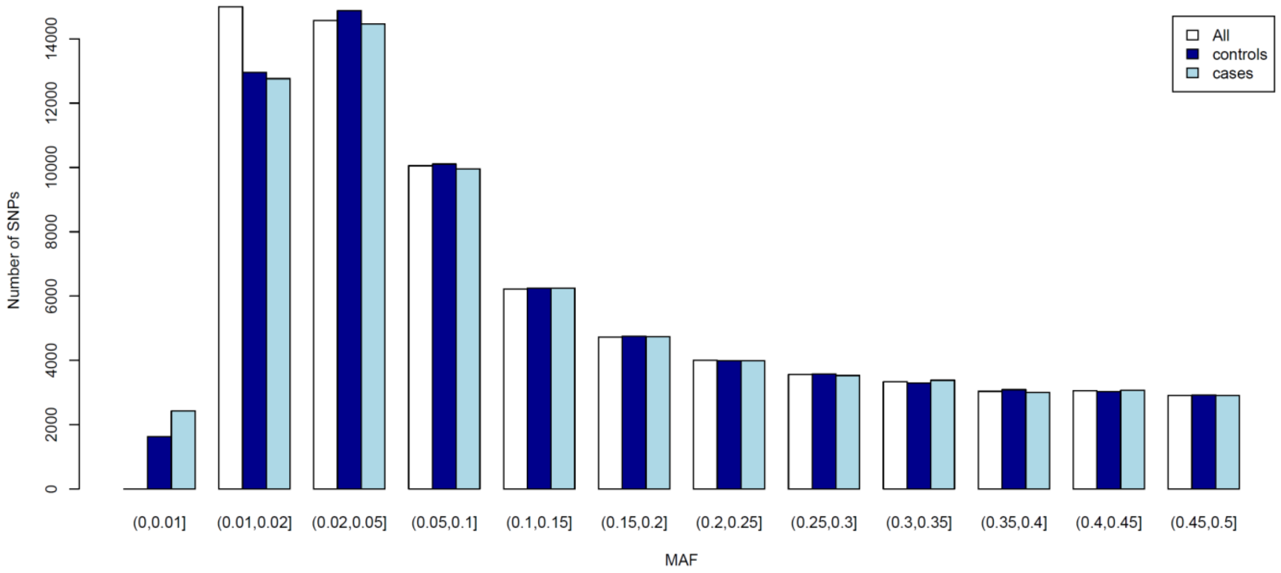

ESM Figure 8   Allele distribution for the inferred polymorphic sites.

The frequency distribution is also shown for cases and controls separately.
