# Supplementary material for: Exome sequencing-driven discovery of coding polymorphisms associated with common metabolic phenotypes
Source: Diabetologia. 2012 Nov 19;56(2):298–310. doi: 10.1007/s00125-012-2756-1 (PMC3536959; doi:10.1007/s00125-012-2756-1)
Supplement: Supplementary file 11 — (PDF 330 kb) [file 125_2012_2756_MOESM11_ESM.pdf]

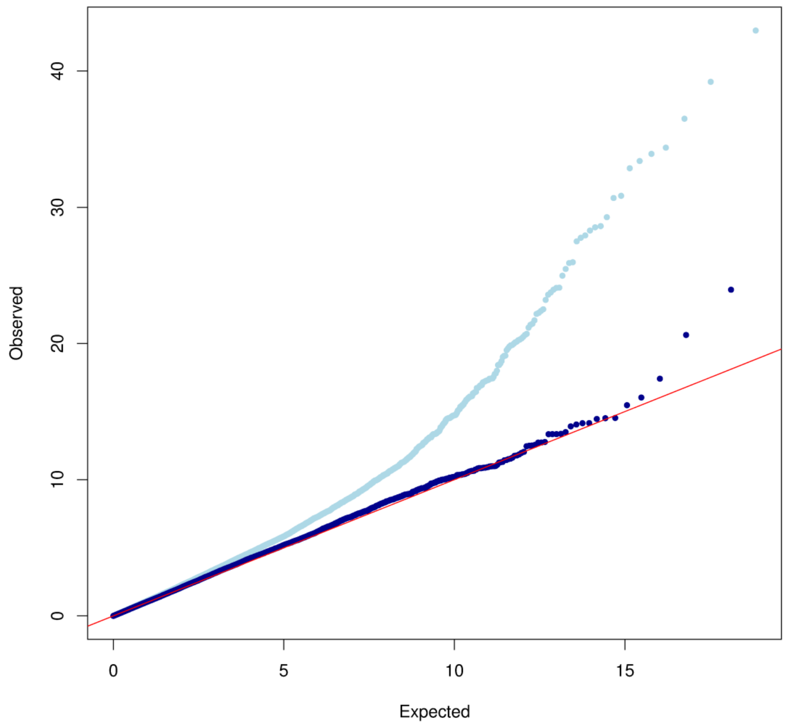

ESM Figure 9 Quantile-quantile plot of test statistics from sequenced-derived SNPs in 1,000 metabolic cases and 1,000 healthy controls (stage 1).

The plot shows the test statistics of all sequenced derived SNPs (*n* = 70,182, light blue dots) and the test statistics after the exclusion of SNPs that correlate with a time-dependent bias (*n* = 48,035, dark blue dots) (ESM Methods section 2.6). After the filtering the inflation rate (λ_{GC}) was 1.05.
