# Supplementary material for: Exome sequencing-driven discovery of coding polymorphisms associated with common metabolic phenotypes
Source: Diabetologia. 2012 Nov 19;56(2):298–310. doi: 10.1007/s00125-012-2756-1 (PMC3536959; doi:10.1007/s00125-012-2756-1)
Supplement: Supplementary file 12 — (PDF 334 kb) [file 125_2012_2756_MOESM12_ESM.pdf]

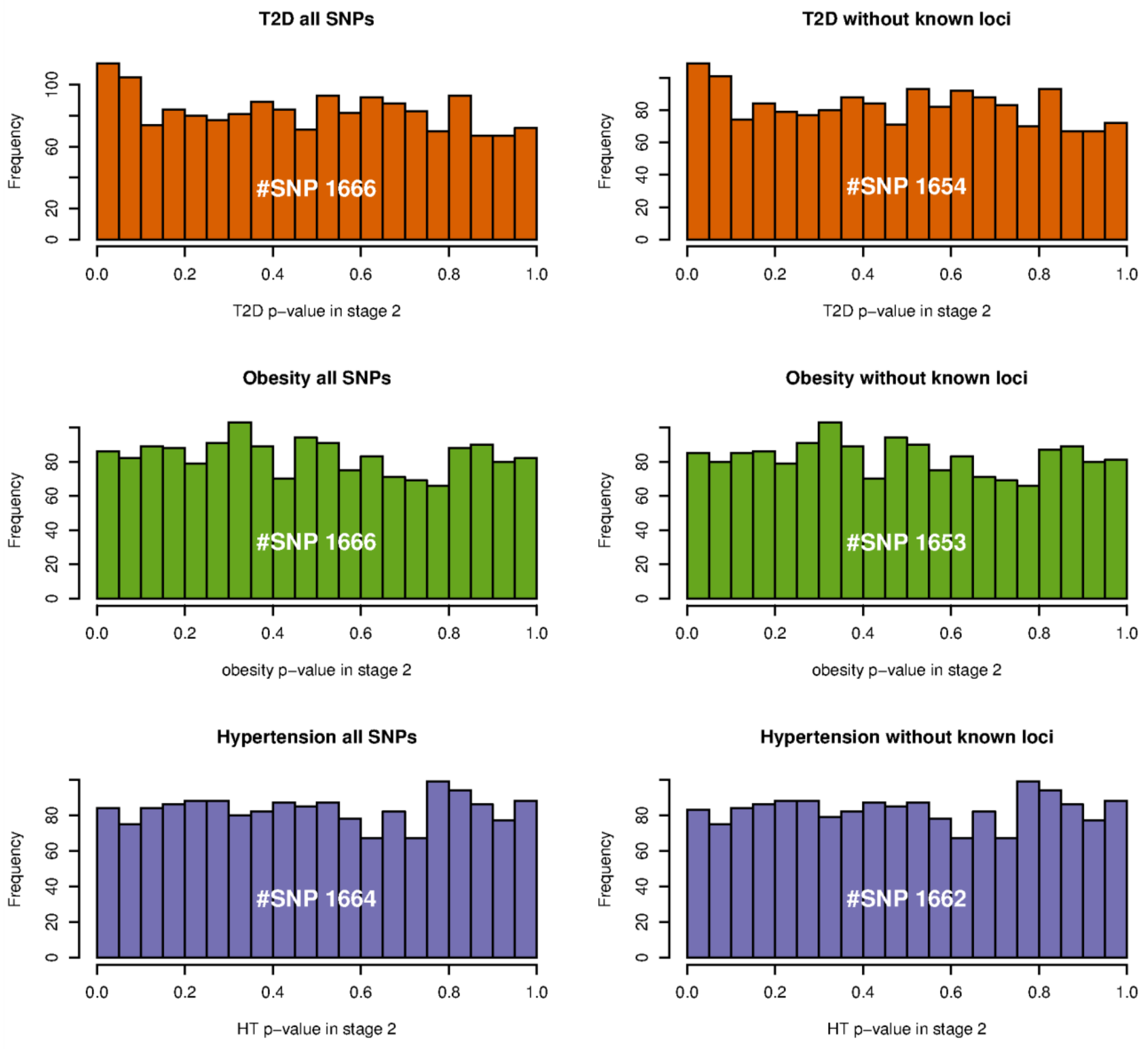

ESM Figure 10 Distribution of genomic control-corrected P-values in stage 2 for the SNPs selected based on their association in stage 1.

The stage 2 individuals present in stage 1 were removed prior to analysis. The left column shows the distribution for all SNPs while the right column shows the distribution after removal of SNPs in loci known to have SNPs associated with metabolic phenotypes. The loci include the nearest genes on both sides of the known associated variant.
