# Supplementary material for: Exome sequencing-driven discovery of coding polymorphisms associated with common metabolic phenotypes
Source: Diabetologia. 2012 Nov 19;56(2):298–310. doi: 10.1007/s00125-012-2756-1 (PMC3536959; doi:10.1007/s00125-012-2756-1)
Supplement: Supplementary file 13 — (PDF 473 kb) [file 125_2012_2756_MOESM13_ESM.pdf]

Top 10

chr	pos	rs	gene	st1.p.value	q.value	p.value
2	165259447	7607980	COBLL1	0.015	0.0081	4.8e−06
4 *	6354988	1046316	WFS1,LOC10	0.011	0.2	0.00028
1	203395942	1062715	RIPK5	0.032	0.26	0.00059
2	65152343	17849707	CEP68	0.037	0.28	0.00086
19	59938624	16985907	KIR3DL3	0.0017	0.29	0.0011
2 *	27584444	1260326	GCKR	0.0042	0.3	0.0011
1	1837839	28581776	CALML6	0.034	0.31	0.0014
6	117351067	7770158	RFXDC1	0.027	0.31	0.0017
22	44102562	62001863	FAM118A	0.017	0.31	0.0017
11	60935861	3019198	FLJ12529	0.00073	0.32	0.0019

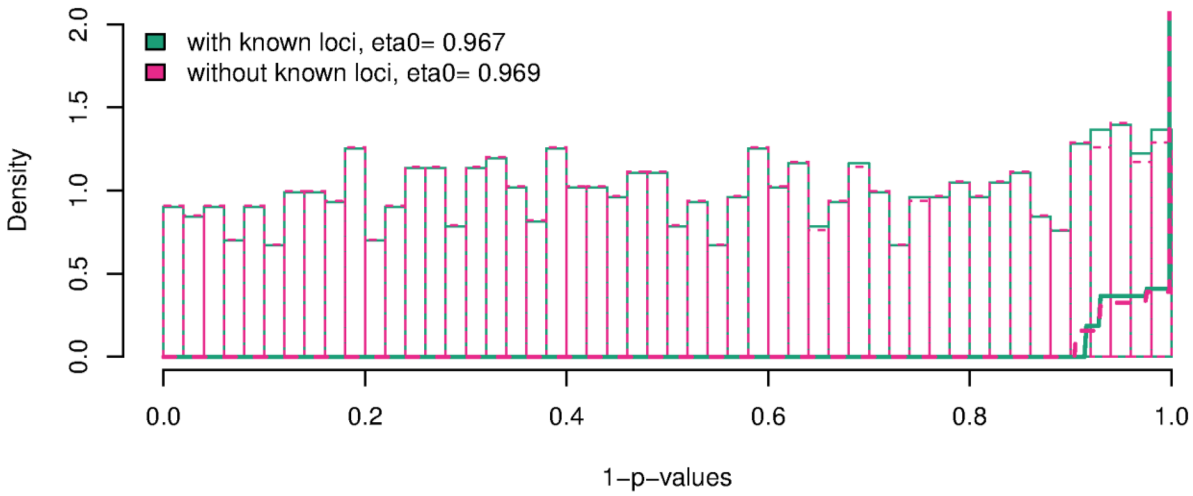

ESM Figure 11 The top associations with type 2 diabetes in stage 2 of the SNPs selected from stage 1 based on association (P < 0.05) in stage 1.

The *P*-value from stage 1 (st.1.p.value) is shown alongside the *P*-value for stage 2 (p.value) estimated without the 2,000 individuals from stage 1. The local false positive rate (q-value) was estimated from the genomic control-corrected *P*-values. *P*-values have not been corrected for multiple testing. The bottom figure shows the histogram of the *P*-values with or without the known associated loci. A mixture model with two components was fitted to the data and the distribution of the associated component is shown in the histogram. *known locus associated with metabolic phenotypes.
