# Supplementary material for: Exome sequencing-driven discovery of coding polymorphisms associated with common metabolic phenotypes
Source: Diabetologia. 2012 Nov 19;56(2):298–310. doi: 10.1007/s00125-012-2756-1 (PMC3536959; doi:10.1007/s00125-012-2756-1)
Supplement: Supplementary file 14 — (PDF 481 kb) [file 125_2012_2756_MOESM14_ESM.pdf]

Top 10

chr	pos	rs	gene	st1.p.value	q.value	p.value
6	117351067	7770158	RFXDC1	0.027	0.69	0.00086
4	108860749	973126	PAPSS1	0.0096	0.69	0.00097
9	89690225	7850542	C9orf79	0.048	0.69	0.0017
16	24485959	7195386	RBBP6	0.019	0.69	0.0021
1	154959759	1133856	ISG20L2	0.018	0.69	0.0024
1	15313616	34135602	RP1−21O18.	0.047	0.69	0.0037
19	63202962	10451433	ZNF606	0.019	0.69	0.0038
19	50087554	157581	TOMM40	0.04	0.69	0.0039
12	51224562	61708395	KRT71	0.032	0.69	0.0043
9	89692827	11789780	C9orf79	0.032	0.69	0.0049

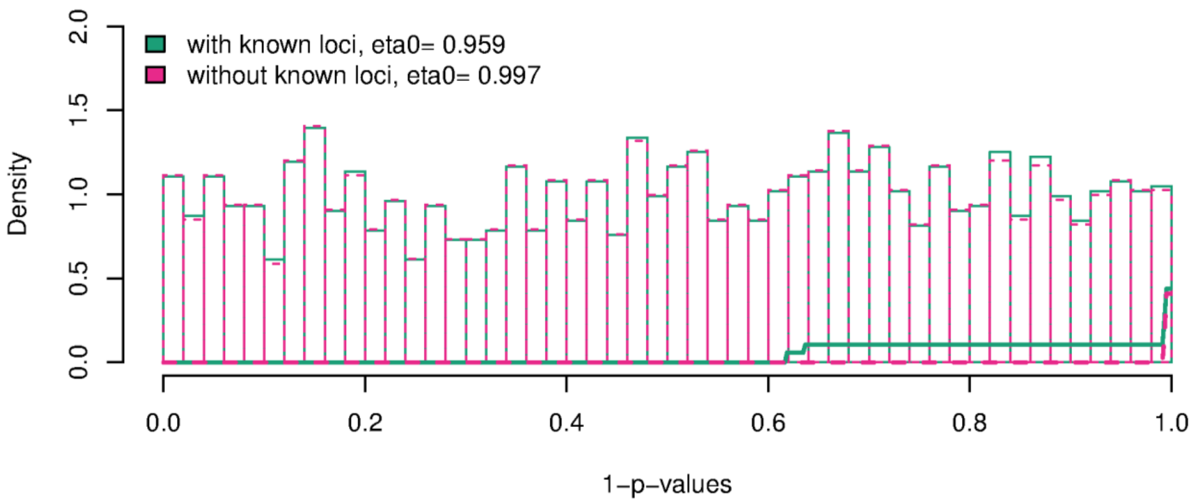

ESM Figure 12 The top associations with obesity in stage 2 of the SNPs selected based on their association (P < 0.05) in stage 1.

The *P*-value from stage 1 (st.1.p.value) is shown alongside the *P*-value for stage 2 (p.value) estimated without the 2,000 individuals from stage 1. The local false positive rate (q-value) was estimated from the genomic control-corrected *P*-values. *P*-values have not been corrected for multiple testing. The bottom figure shows the histogram of the *P*-values with or without the known associated loci. A mixture model with two components was fitted to the data and the distribution of the associated component is shown in the histogram. *known locus associated with metabolic phenotypes.
