# Supplementary material for: Exome sequencing-driven discovery of coding polymorphisms associated with common metabolic phenotypes
Source: Diabetologia. 2012 Nov 19;56(2):298–310. doi: 10.1007/s00125-012-2756-1 (PMC3536959; doi:10.1007/s00125-012-2756-1)
Supplement: Supplementary file 15 — (PDF 473 kb) [file 125_2012_2756_MOESM15_ESM.pdf]

Top 10						
chr	pos	rs	gene	st1.p.value	q.value	p.value
1	28436839	8559	ATPIF1	0.0059	0.7	0.00059
16	68525398	2270841	WWP2	0.042	0.7	0.0013
17	5279005	56014026	C1QBP	0.034	0.7	0.0013
21	30724639	2226548	KRTAP13−4	0.026	0.7	0.0028
8	20073257	17092129	SLC18A1	0.003	0.7	0.0031
14	51578813	17831525	NID2	0.031	0.7	0.0033
22	17280868	16983347	PRODH	0.031	0.7	0.0035
17	11724196		DNAH9	0.012	0.7	0.0042
3	16243990	14576	GALNTL2	0.027	0.7	0.0043
17	19753133	203462	AKAP10	0.027	0.7	0.006

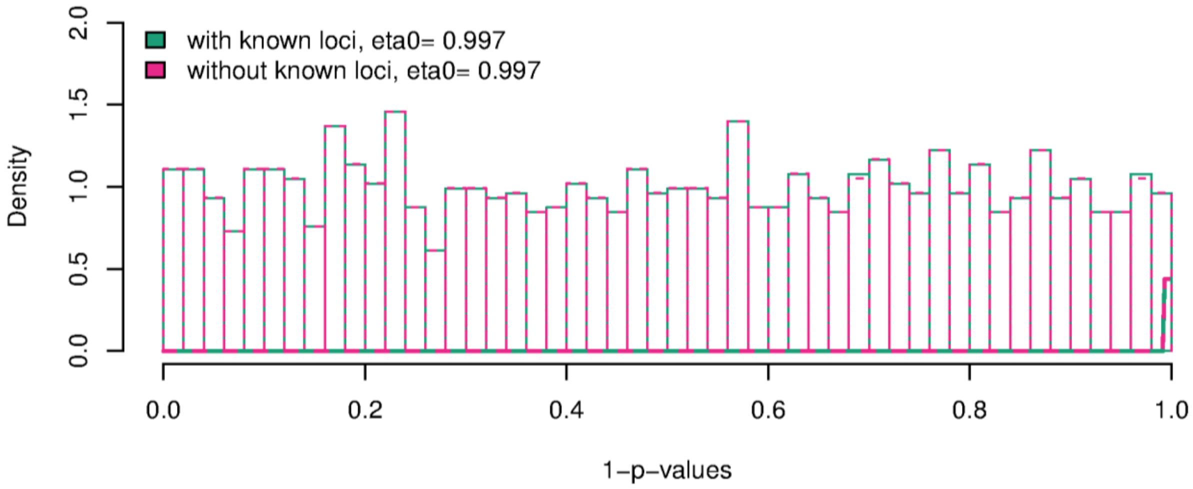

ESM Figure 13 The top associations with hypertension in stage 2 of the SNPs selected based on their association (P < 0.05) in stage 1.

The *P*-value from stage 1 (st.1.p.value) is shown alongside the *P*-value for stage 2 (p.value) estimated without the 2,000 individuals from stage 1. The local false positive rate (q-value) was estimated from the genomic control-corrected *P*-values. *P*-values have not been corrected for multiple testing. The bottom figure shows the histogram of the *P*-values with or without the known associated loci. A mixture model with two components was fitted to the data and the distribution of the associated component is shown in the histogram. *known locus associated with metabolic phenotypes.
