# Supplementary material for: Exome sequencing-driven discovery of coding polymorphisms associated with common metabolic phenotypes
Source: Diabetologia. 2012 Nov 19;56(2):298–310. doi: 10.1007/s00125-012-2756-1 (PMC3536959; doi:10.1007/s00125-012-2756-1)
Supplement: Supplementary file 16 — (PDF 341 kb) [file 125_2012_2756_MOESM16_ESM.pdf]

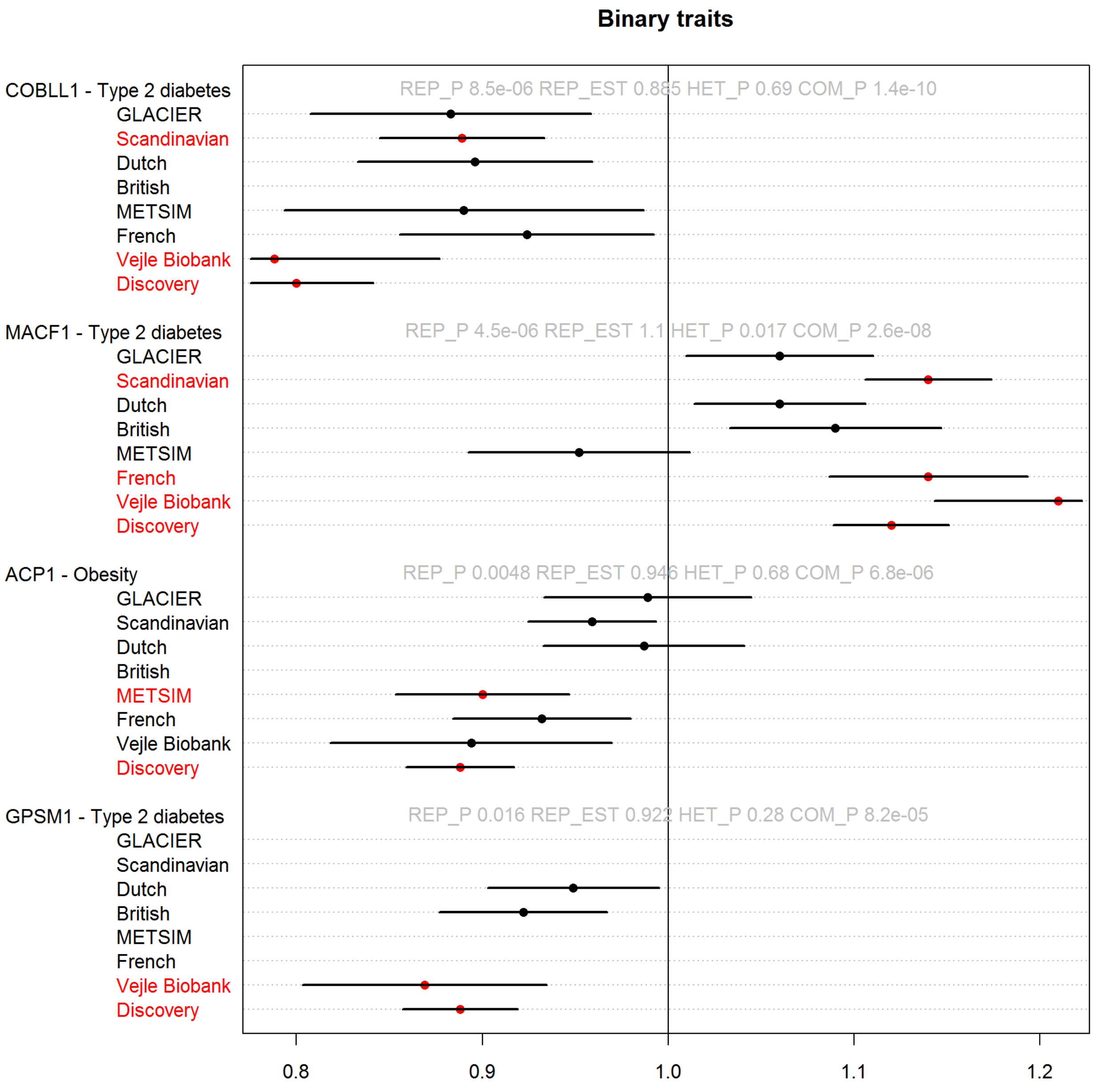

ESM Figure 14 Replication of selected coding SNPs with binary metabolic phenotypes in stage 3 European samples.

For each association is shown the effect in discovery (stage 2) samples and the cohort-specific replication effect. In red are studies with a nominally significant association ($P < 0.05$). Data are OR and SE. REP_P: *P*-value in replication samples, REP_EST: Effect size (OR) in replication samples, HET_P: *P*-value for heterogeneity in replication samples, COM_P: Combined *P*-value.
