# Supplementary material for: Exome sequencing-driven discovery of coding polymorphisms associated with common metabolic phenotypes
Source: Diabetologia. 2012 Nov 19;56(2):298–310. doi: 10.1007/s00125-012-2756-1 (PMC3536959; doi:10.1007/s00125-012-2756-1)
Supplement: Supplementary file 18 — (PDF 391 kb) [file 125_2012_2756_MOESM18_ESM.pdf]

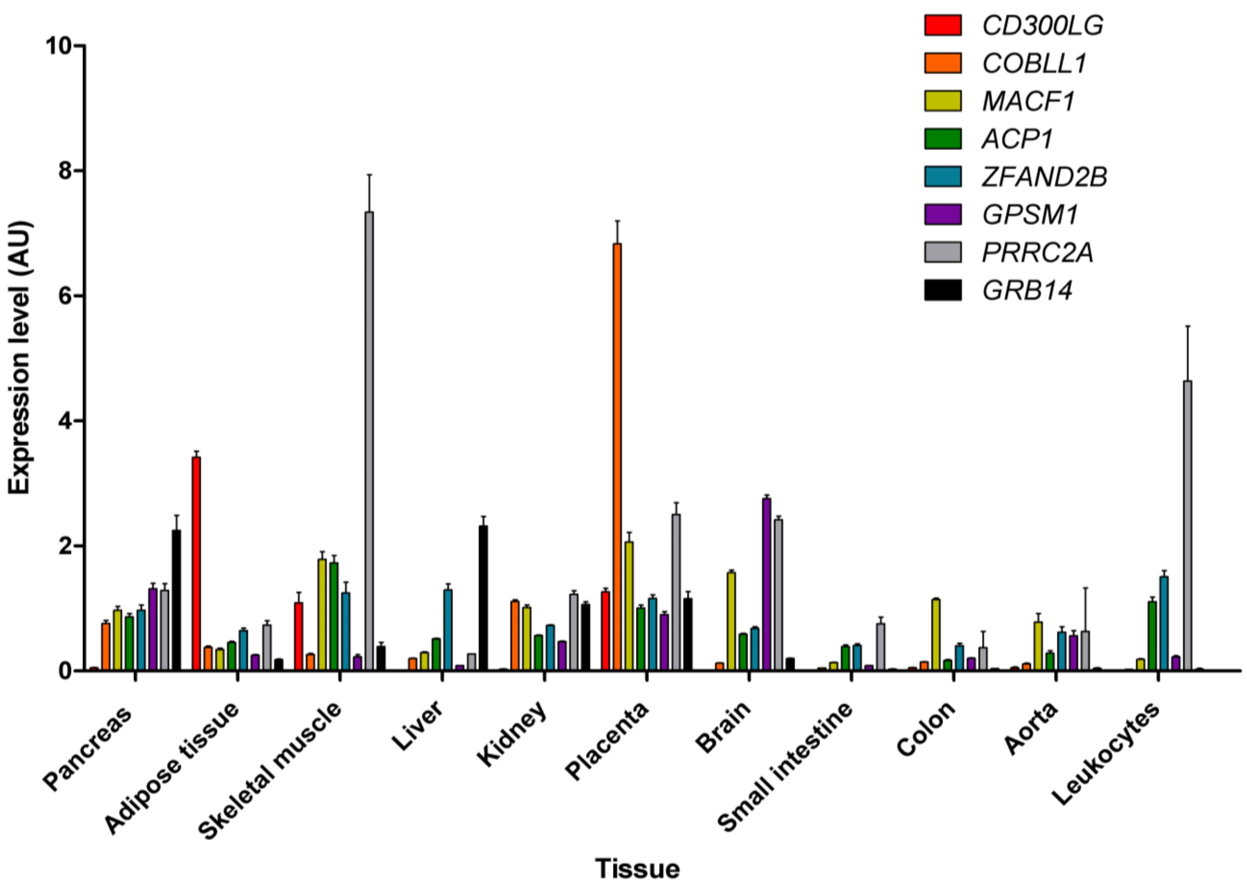

ESM Figure 16 Tissue expression of genes containing coding variants associated with metabolic traits.

Expression levels of *CD300LG*, *COBLL1*, *MACF1*, *ACP1*, *ZFAND2B*, *GPSM1*, *PRRC2A* and *GRB14* were quantified in duplicates by TaqMan real-time PCR in a human tissue mRNA panel including pancreas, adipose tissue, skeletal muscle, liver, kidney, placenta, total brain, total small intestine, colon, aorta and leukocytes. Results are presented as mean ± SD.
