# Supplementary material for: Exome sequencing-driven discovery of coding polymorphisms associated with common metabolic phenotypes
Source: Diabetologia. 2012 Nov 19;56(2):298–310. doi: 10.1007/s00125-012-2756-1 (PMC3536959; doi:10.1007/s00125-012-2756-1)
Supplement: Supplementary file 19 — (PDF 420 kb) [file 125_2012_2756_MOESM19_ESM.pdf]

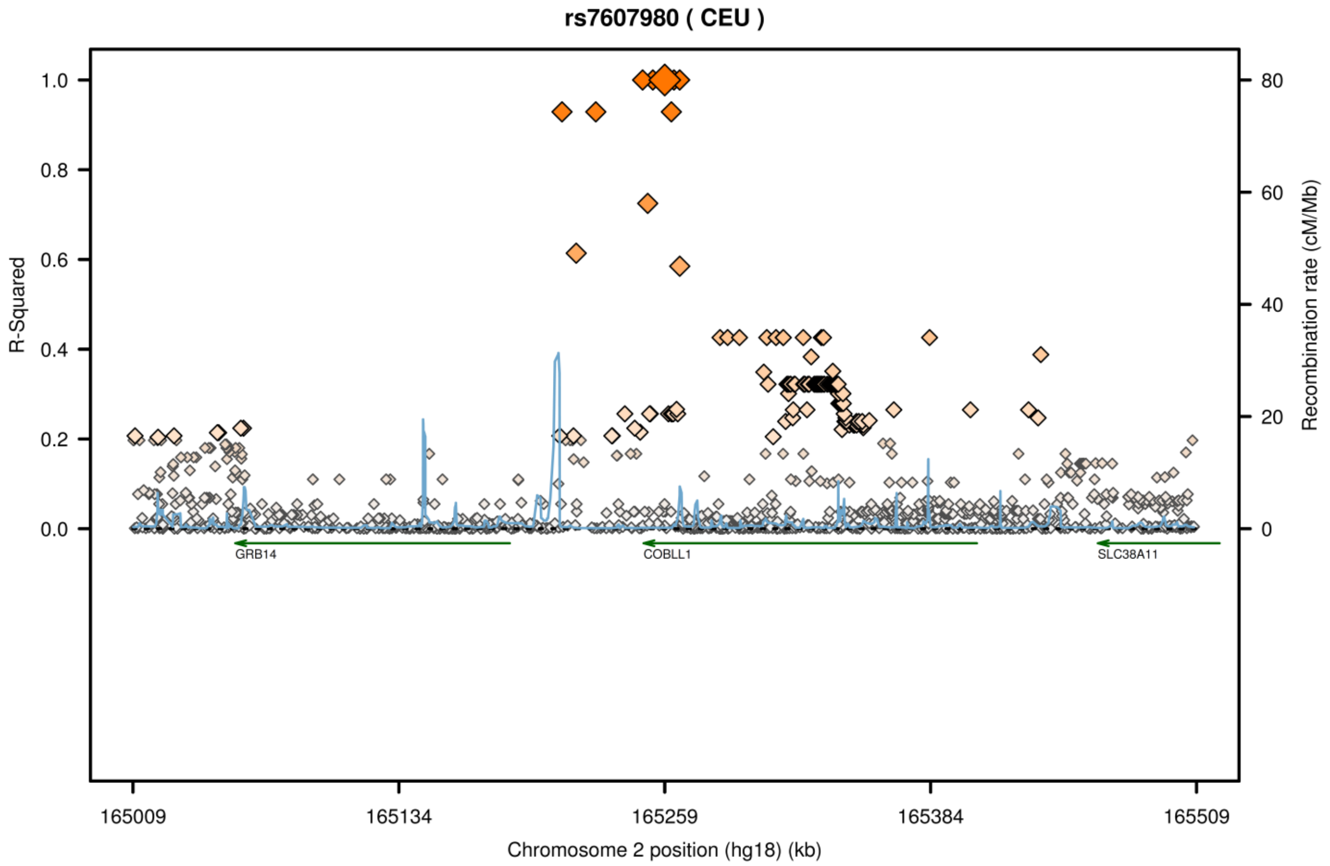

ESM Figure 17 Linkage disequilibrium between sequence variants in the COBLL1/GRB14 region

Plot of linkage disequilibrium (r^2) with rs7607980 for SNPs located +/- 250 kb from rs7608980 based on data from 1000 Genomes Project. Size and colour of the diamonds indicate the level of LD with rs7608980 as high (r^2>0.5) in orange, medium (0.5>r^2>0.2) in light orange and low (r^2<0.2) in pale. The blue line displays the recombination rate. The plot was made using SNAP (http://www.broadinstitute.org/mpg/snap/; Johnson, A. D., Handsaker, R. E., Pulit, S., Nizzari, M. M., O'Donnell, C. J., de Bakker, P. I. W.
SNAP: A web-based tool for identification and annotation of proxy SNPs using HapMap. *Bioinformatics, 2008 24(24):2938-2939*)
