# Supplementary material for: Exome sequencing-driven discovery of coding polymorphisms associated with common metabolic phenotypes
Source: Diabetologia. 2012 Nov 19;56(2):298–310. doi: 10.1007/s00125-012-2756-1 (PMC3536959; doi:10.1007/s00125-012-2756-1)
Supplement: Supplementary file 20 — (PDF 200 kb) [file 125_2012_2756_MOESM20_ESM.pdf]

ESM Table 1 Clinical and biochemical characteristics of five study groups involved in discovery studies of stage 1 and/or stage 2

	Inter99	Steno Diabetes Center	ADDITION	Health2006	Vejle Biobank
N	5887	2154	3711	2902	1335
Sex (M/W)	2892/2995	1303/851	1960/1751	1311/1591	676/659
Age (years)	46.1 ± 7.9	61.8 ± 11	59.5 ± 7	49.2 ± 13	61.1 ± 10
HbA_{1C} (%)	5.8 (5.5-6.1)	5.8 (5.5-6.2)	5.9 (5.6-6.3)	5.4 (5.1-5.6)	6.3 (5.5-7.2)
BMI (kg/m2)	26.3 ± 4.6	28.3 ± 5.4	32.2 ± 4.7	26 ± 4.7	26.5 ± 5.7
Waist circumference (cm)	86.6 ± 13	96.7 ± 16	106 ± 12	88.8 ± 14	93.7 ± 16
Fasting plasma glucose (mmol/l)	5.53 ± 0.79	5.24 ± 1	NA	5.44 ± 0.62	5.11 ± 0.32
Fasting serum cholesterol (mmol/l)	5.52 ± 1.1	5.63 ± 1.2	5.8 ± 1.1	5.35 ± 1.1	4.6 ± 0.99
Fasting serum HDL-cholesterol (mmol/l)	1.44 ± 0.4	1.25 ± 0.43	1.44 ± 0.38	1.54 ± 0.43	1.45 ± 0.44
Fasting serum triacylglycerol (mmol/l)	1.1 (0.8-1.5)	1.5 (1-2.3)	1.5 (1.1-2.0)	1.1 (0.8-1.5)	1.2 (0.9-1.8)
Systolic blood pressure (mmHg)	129 (120-140)	137 (123-153)	140 (130-151)	126 (117-138)	144 (130-159)
Diastolic blood pressure (mmHg)	80 (75-90)	79 (72-86)	85 (80-90)	80 (74-88)	84 (77-91)

Data are mean ± SD for normally distributed traits or median (interquartile range). The different Danish study samples are described in ESM Methods section 1.1.
