# Supplementary material for: Exome sequencing-driven discovery of coding polymorphisms associated with common metabolic phenotypes
Source: Diabetologia. 2012 Nov 19;56(2):298–310. doi: 10.1007/s00125-012-2756-1 (PMC3536959; doi:10.1007/s00125-012-2756-1)
Supplement: Supplementary file 21 — (PDF 199 kb) [file 125_2012_2756_MOESM21_ESM.pdf]

ESM Table 2 Clinical and biochemical characteristics of 2,000 individuals involved in exome sequencing

	Metabolic cases	Controls
N	1000	1000
N (Inter99)	36	851
N (Steno Diabetes Center)	610	149
N (ADDITION)	354	0
N (Health2006)	0	0
N (Vejle Biobank)	0	0
Sex (M/W)	499/501	501/499
Age (years)	58.3 ± 8.5	50.7 ± 6.7
Age at diagnosis of type 2 diabetes (years)*	49.4 (43.7-54.1)	-
HbA_{1C} (%)	6.7 (6.1-8.1)	5.8 (5.5-6.1)
BMI (kg/m2)	33.8 ± 4.9	23.5 ± 2.1
Waist circumference (cm)	111 ± 12	80.4 ± 9.1
Fasting plasma glucose (mmol/l)	9.9 ± 3.6	5.1 ± 0.3
Fasting serum cholesterol (mmol/l)	5.9 ± 1.1	5.5 ± 1.0
Fasting serum HDL-cholesterol (mmol/l)	1.3 ± 0.35	1.6 ± 0.42
Fasting serum triacylglycerol (mmol/l)	1.8 (1.4-2.5)	0.90 (0.7-1.2)
Systolic blood pressure (mmHg)	150 (140-160)	125 (115-133)
Diastolic blood pressure (mmHg)	90 (85-95)	80 (71-85)

Description of 1,000 cases and 1,000 control individual participating in the exome sequencing. Data are mean ± SD for normally distributed traits and median (interquartile range).

* Age at diagnosis is only given for patients with clinical-onset type 2 diabetes
