# Supplementary material for: Exome sequencing-driven discovery of coding polymorphisms associated with common metabolic phenotypes
Source: Diabetologia. 2012 Nov 19;56(2):298–310. doi: 10.1007/s00125-012-2756-1 (PMC3536959; doi:10.1007/s00125-012-2756-1)
Supplement: Supplementary file 23 — (PDF 197 kb) [file 125_2012_2756_MOESM23_ESM.pdf]

ESM Table 4   Summary of data production of exome sequencing of 2,000 DNA samples

	Mean	Standard deviation
Number of raw reads (million)	22.3	3.9
Raw data yield (Mb)	1637.9	295.6
Number of effective reads (million)	15.5	2.5
Average read length* (bp)	65.2	5.9
Effective data yield (Mb)	1015.2	198.9
Effective reads mapped to exome** regions (%)	45.5***	5.4
Average depth for target regions	10.9	2.2
Coverage of target region (%)	95.5	1.3
Rate of nucleotide mismatches (%)	1.04	0.23

Raw reads denote reads after removal of linker and adapter sequences. Effective reads are aligned reads after removal of linker and adapter sequence.* The read length ranged from 30 bp to 80 bp. **.

*** This is the percentage of reads that were mapped into or overlapped with target regions. It is not equal to the percentage of data that are mapped to the target regions.

The sequencing process was stopped if the real-time QC feedback gave negative information.

The standard deviations are derived from statistics of the 2,000 DNA samples.
