# Supplementary material for: Exome sequencing-driven discovery of coding polymorphisms associated with common metabolic phenotypes
Source: Diabetologia. 2012 Nov 19;56(2):298–310. doi: 10.1007/s00125-012-2756-1 (PMC3536959; doi:10.1007/s00125-012-2756-1)
Supplement: Supplementary file 24 — (PDF 198 kb) [file 125_2012_2756_MOESM24_ESM.pdf]

ESM Table 5 Non-redundant functional category annotations

Type	1000 Genomes	dbSNP	dbSNP & 1000 Genomes	Novel	All
Nonsense	28	36	78	101	243
Nonsynonymous	1130	3594	11061	4417	20202
Splice site	52	25	31	193	301
UTR	230	390	1579	557	2756
Synonymous	805	3377	13968	2101	20251
Near gene	24	26	135	54	239
Intron	3278	2626	13920	5968	25801
Intergenic	46	59	187	97	389
Total	5602	10133	40959	13488	70182

Non-redundant meaning that a SNP can enter the table only once. Annotations were obtained using the SeattleSeq Annotation Build 5.0. The 1,000 genomes SNPs are from the April 2009 release and dbSNPs from build 129. Note that 37 SNPs could not be annotated because the number of coding bases was not a multiple of 3.
