# Supplementary material for: Exome sequencing-driven discovery of coding polymorphisms associated with common metabolic phenotypes
Source: Diabetologia. 2012 Nov 19;56(2):298–310. doi: 10.1007/s00125-012-2756-1 (PMC3536959; doi:10.1007/s00125-012-2756-1)
Supplement: Supplementary file 25 — (PDF 317 kb) [file 125_2012_2756_MOESM25_ESM.pdf]

ESM Table 6 Overview of replication efforts for 51 selected associations covered by 45 unique SNPs

Trait	Gene	SNP	CHR	POS	Vejle Biobank	French	METSIM	British	Dutch	Scandinavian	GLACIER	Total
Type 2 diabetes	*COBLL1*	rs7607980	2	165259447	2448	6750	4706		5324	12292	4887	36407
Type 2 diabetes	*MACF1*	rs2296172	1	39608404	2462	6662	5790	26080	5350	12686	4866	63896
Type 2 diabetes	*GPSM1*	rs60980157	9	138355236	2455			26080	5280			33815
Type 2 diabetes	*C9*	rs34882957	5	39367651	2468	6768	4712			12718	4926	31592
Type 2 diabetes	*LMTK2*	rs56204700	7	97659791	2449	6766	5816			12731	4927	32689
Type 2 diabetes	*GPNMB*	chr7_23272666	7	23272666	2454	6800	5818			12725	4936	32733
Type 2 diabetes	*PHF15*	chr5_133929838	5	133929838	2450	6801	4697	13663		12725	4935	45271
Type 2 diabetes	*C1orf222*	rs28633659	1	1844406	2459	6785	4704	26080		12254	4898	57180
Type 2 diabetes	*ELOVL3*	rs36103207	10	103978255	2462	6841	5800	26080		12675	4931	58789
Type 2 diabetes	*KIR3DL3*	rs16985907	19	59938624	2448	6807	5767			12342	4826	32190
Type 2 diabetes	*AQP8*	chr16_25147310	16	25147310	2436	6772	4713			12741	4935	31597
Type 2 diabetes	*NISCH*	rs9856575	3	52485632	2455	6768	5787			12739	4926	32675
Obesity	*ACP1*	rs11553746	2	262203	1494	4600	4170		3065	7703	2712	23744
Obesity	*PML*	rs743581	15	72115194	1503	4655				7751	2719	16628
Obesity	*MED20*	chr6_41982766	6	41982766	1503	4682	3127			7763	2719	19794
Obesity	*C10orf47*	rs2147439	10	11951710	1507		4180	9049		7771	2680	25187
Obesity	*TCERG1L*	chr10_132851444	10	132851444	1494	4665	4125			7758	2724	20766
Obesity	*GDAP1L1*	rs2425632	20	42325348	1504	4693	4188			7776	2720	20881
Obesity	*OR2Y1*	rs11960429	5	180099283	1512	4618	3397			7775	2725	20027
Obesity	*INHA*	rs12720062	2	220148160	1512	4681				7741	2609	16543
Obesity	*TOMM40*	rs157581	19	50087554	1498	4686				7727	2724	16635
Obesity	*SLC27A4*	rs2240953	9	130150713	1512	4621	3397	9049			2676	21255
Obesity	*ANKH*	chr5_14924560	5	14924560	1496		4185	3906		7735	2634	19956
BMI	*ACP1*	rs11553746	2	262203	2514	6569	8108		5304	12534	4902	39931
BMI	*TGIF1*	rs4468717	18	3447606	2526	6691						9217
BMI	*SIM2*	chr21_37003350	21	37003350	2537		8131			12527		23195
BMI	*CCDC165*	rs35739383	18	8773835	2539	6616				12441		21596
BMI	*SLC27A4*	rs2240953	9	130150713	2547	6599	6635	15995			4834	36610
Waist circumference	*ACP1*	rs11553746	2	262203	2528	5705	8121		5103	6331	1256	29044
Waist circumference	*OR10Z1*	rs41273513	1	156843597	2548	5814	8149			6360	1263	24134

Trait	Gene	SNP	CHR	POS	Vejle Biobank	French	METSIM	British	Dutch	Scandinavian	GLACIER	Total
Waist circumference	*ELOVL3*	rs36103207	10	103978255	2555	5858	8127	13019		6372	1255	37186
Waist circumference	*SLC27A4*	rs2240953	9	130150713	2560	5732	6644	13019			1247	29202
Fasting plasma glucose	*ZFAND2B*	rs61750009	2	219780675	1248	5254	7001		2279	6436	3342	25560
Fasting plasma glucose	*CSAD*	chr12_51840193	12	51840193	1260	5287	5731		2289	6395	3308	24270
Fasting plasma glucose	*PTPRH*	rs16986309	19	60401886	1257	5201	7019			6417	3337	23231
Fasting plasma glucose	*ELOVL3*	rs36103207	10	103978255	1257	5269	6999			6379	3350	23254
Fasting plasma glucose	*EIF2B4*	rs1058065	2	27441228	1253	5233	5689			6435	3340	21950
Fasting plasma glucose	*ABCC2*	rs41318029	10	101580476	1252	5268	7024			6434	3341	23319
Fasting plasma glucose	*BRD3*	rs34609592	9	135905516	1259	5269	5727			6442	3352	22049
Fasting plasma glucose	*CASP4*	rs1801319	11	104330859	1242		7023			6427	3342	18034
Fasting serum insulin	*GRK4*	rs35605687	4	2985351		5214	5711			6331		17256
Fasting serum insulin	*DST*	chr6_56588459	6	56588459		5202	7018					12220
Fasting serum insulin	*ENTPD5*	rs12434581	14	73524572		5135						5135
Fasting serum insulin	*PHC2*	rs41265897	1	33608751		5232	6998			6247		18477
Fasting serum insulin	*FBN3*	chr19_8082890	19	8082890		5111	7015			6284		18410
Fasting serum insulin	*C13orf35*	chr13_112385690	13	112385690		5203	7004			6349		18556
Fasting serum insulin	*DHX58*	rs35118457	17	37516926		5166	5706			6323		17195
Fasting serum insulin	*AKAP12*	rs41289373	6	151714763		5179	7017			6351		18547
Fasting plasma HDL-cholesterol	*CD300LG*	chr17_39281652	17	39281652	1057	5006	5291		2095	6155	1218	20822
Fasting plasma HDL-cholesterol	*SLC26A2*	chr5_149340823	5	149340823	1057	5018	5331			6184	1214	18804
Fasting plasma triacylglycerol	*PRRC2A*	rs41273264	6	31708085	1058	5048	5208		2103		2718	16135

Data are number of genotyped individuals in each replication study sample and in total for each of 51 associations. More information on the replication cohorts is given in ESM Methods section 1.3. SNP information is based on dbSNP 129 and genome build 36. The very variable numbers of samples for each study sample and SNP is due to 1) not all SNPs were genotyped in all cohorts and 2) not all cohorts have information on all traits.
