# Supplementary material for: Exome sequencing-driven discovery of coding polymorphisms associated with common metabolic phenotypes
Source: Diabetologia. 2012 Nov 19;56(2):298–310. doi: 10.1007/s00125-012-2756-1 (PMC3536959; doi:10.1007/s00125-012-2756-1)
Supplement: Supplementary file 26 — (PDF 321 kb) [file 125_2012_2756_MOESM26_ESM.pdf]

ESM Table 7   51 associations for eight metabolic traits selected for replication in stage 3

Trait	Gene	rs-number	Chr-Pos	Effect allele	Non effect allele	MAF	MAF-HapMap	Function	Mutation	*n*	Effect (95% CI)	*P*-value
Type 2 diabetes	*COBLL1*	rs7607980	2-165259447	C	T	13	16	Nonsyn	N939D	12177	0.80 (0.74 to 0.87)	3×10^{-7}
Type 2 diabetes	*KIR3DL3*	rs16985907	19-59938624	C	G	4.1	4.2	Nonsyn	A348P	12179	1.31 (1.16 to 1.49)	7.5×10^{-5}
Type 2 diabetes	*C1orf222*	rs28633659	1-1844406	T	C	3.9		Nonsyn	E187K	12179	0.76 (0.66 to 0.87)	2×10^{-4}
Type 2 diabetes	*GPSM1*	rs60980157	9-138355236	T	C	25		Nonsyn	S368L	12175	0.89 (0.84 to 0.94)	0.00028
Type 2 diabetes	*AQP8*		16-25147310	A	G	2.4		Nonsyn	R261Q	12175	0.71 (0.59 to 0.85)	0.00038
Type 2 diabetes	*C9*	rs34882957	5-39367651	A	G	0.97		Nonsyn	P167S	12177	0.58 (0.44 to 0.77)	0.00043
Type 2 diabetes	*NISCH*	rs9856575	3-52485632	G	A	2.1	0.4	Nonsyn	I299V	12177	1.40 (1.17 to 1.68)	0.00055
Type 2 diabetes	*GPNMB*		7-23272666	A	G	2.2		Nonsyn	A354T	12179	1.39 (1.16 to 1.65)	0.00055
Type 2 diabetes	*MACF1*	rs2296172	1-39608404	G	A	23	18	Nonsyn	M2290V	12175	1.12 (1.05 to 1.19)	0.00065
Type 2 diabetes	*LMTK2*	rs56204700	7-97659791	C	T	2.2		Nonsyn	I693T	12179	1.38 (1.16 to 1.65)	0.00076
Type 2 diabetes	*ELOVL3*	rs36103207	10-103978255	A	G	3.7		Nonsyn	D109N	12178	1.28 (1.12 to 1.47)	0.00083
Type 2 diabetes	*PHF15*		5-133929838	T	G	1.4		Nonsyn	G368V	12151	0.66 (0.52 to 0.83)	0.00089
Obesity	*ACP1*	rs11553746	2-262203	T	C	38	30	Nonsyn	T95I	10324	0.89 (0.84 to 0.94)	9.4×10^{-5}
Obesity	*TCERG1L*		10-132851444	T	C	3.7		Nonsyn	G322E	10346	1.37 (1.18 to 1.59)	0.00013
Obesity	*INHA*	rs12720062	2-220148160	A	G	2.1		Nonsyn	A257T	10306	0.68 (0.56 to 0.83)	0.00038
Obesity	*GDAP1L1*	rs2425632	20-42325348	T	C	4.2		Syn		10346	0.78 (0.68 to 0.89)	0.00049
Obesity	*C10orf47*	rs2147439	10-11951710	A	G	1.5		Nonsyn	E203K	10346	1.53 (1.22 to 1.93)	0.00057
Obesity	*ANKH*		5-14924560	T	C	7.7		5'UTR		10346	0.83 (0.75 to 0.92)	0.00073
Obesity	*SLC27A4*	rs2240953	9-130150713	A	G	4.2		Nonsyn	G209S	10308	1.29 (1.12 to 1.48)	0.00073
Obesity	*TOMM40*	rs157581	19-50087554	C	T	24		Syn		10344	0.89 (0.83 to 0.95)	0.00076
Obesity	*PML*	rs743581	15-72115194	T	G	37		Nonsyn	G780V	10346	1.11 (1.05 to 1.17)	0.00079
Obesity	*MED20*		6-41982766	A	C	1.6		3'UTR		10345	0.67 (0.54 to 0.84)	0.00085
Obesity	*OR2Y1*	rs11960429	5-180099283	A	G	1.2	2.1	Nonsyn	R128C	10346	0.64 (0.5 to 0.82)	0.00098
BMI	*ACP1*	rs11553746	2-262203	T	C	38	30	Nonsyn	T95I	14793	-0.06 (0.01)	1.1×10^{-5}
BMI	*SLC27A4*	rs2240953	9-130150713	A	G	4.2		Nonsyn	G209S	14776	0.12 (0.03)	0.00017
BMI	*CCDC165*	rs35739383	18-8773835	T	C	40		Nonsyn	M242T	14820	-0.04 (0.01)	0.00051
BMI	*TGIF1*	rs4468717	18-3447606	T	C	7.2		Nonsyn	P163S	14759	0.08 (0.02)	0.00064
BMI	*SIM2*		21-37003350	T	C	5.1		Nonsyn	A63V	14827	-0.09 (0.03)	0.00076
Waist circumference	*SLC27A4*	rs2240953	9-130150713	A	G	4.2		Nonsyn	G209S	14496	0.12 (0.03)	4.1×10^{-5}

Trait	Gene	rs-number	Chr-Pos	Effect allele	Non effect allele	MAF	MAF-HapMap	Function	Mutation	*n*	Effect (95% CI)	*P*-value
Waist circumference	*ACP1*	rs11553746	2-262203	T	C	38	30	Nonsyn	T95I	14513	-0.05 (0.01)	0.00011
Waist circumference	*OR10Z1*	rs41273513	1-156843597	G	A	1.5		Nonsyn	I249V	14544	-0.17 (0.05)	0.00045
Waist circumference	*ELOVL3*	rs36103207	10-103978255	A	G	3.7		Nonsyn	D109N	14545	0.10 (0.03)	0.00083
Fasting plasma glucose	*CASP4*	rs1801319	11-104330859	T	C	4.3		5'UTR		9087	0.13 (0.03)	0.00019
Fasting plasma glucose	*EIF2B4*	rs1058065	2-27441228	A	G	2.1	4.2	Syn		9087	0.18 (0.05)	3×10^{-4}
Fasting plasma glucose	*ELOVL3*	rs36103207	10-103978255	A	G	3.7		Nonsyn	D109N	9086	0.13 (0.04)	0.00055
Fasting plasma glucose	*BRD3*	rs34609592	9-135905516	A	G	1.7		Nonsyn	A172V	9087	0.19 (0.05)	0.00056
Fasting plasma glucose	*CSAD*		12-51840193	T	C	1		Nonsyn	D382N	9087	-0.24 (0.07)	0.00056
Fasting plasma glucose	*ZFAND2B*	rs61750009	2-219780675	A	C	4.2		Nonsyn	P71H	9080	-0.12 (0.03)	0.00065
Fasting plasma glucose	*ABCC2*	rs41318029	10-101580476	A	G	1.6		Nonsyn	G921S	9087	0.19 (0.06)	0.00085
Fasting plasma glucose	*PTPRH*	rs16986309	19-60401886	A	G	11	17	Nonsyn	L543F	9087	-0.08 (0.02)	0.00087
Fasting serum insulin	*GRK4*	rs35605687	4-2985351	A	G	15	16	Nonsyn	V215I	8417	-0.10 (0.02)	1.1×10^{-5}
Fasting serum insulin	*DST*		6-56588459	C	T	3.6		Nonsyn	I2589V	8418	-0.18 (0.04)	1.7×10^{-5}
Fasting serum insulin	*ENTPD5*	rs12434581	14-73524572	A	T	41		5'UTR		8413	-0.06 (0.02)	5.2×10^{-5}
Fasting serum insulin	*AKAP12*	rs41289373	6-151714763	A	G	1.3		Nonsyn	A1182T	8381	-0.25 (0.06)	9×10^{-5}
Fasting serum insulin	*C13orf35*		13-112385690	T	C	1.7		Nonsyn	R121W	8419	-0.21 (0.06)	0.00042
Fasting serum insulin	*PHC2*	rs41265897	1-33608751	A	G	4		Nonsyn	A145V	8417	-0.14 (0.04)	0.00042
Fasting serum insulin	*DHX58*	rs35118457	17-37516926	T	C	5.8		Nonsyn	R95Q	8418	0.11 (0.03)	9×10^{-4}
Fasting serum insulin	*FBN3*		19-8082890	G	C	2.6		Nonsyn	G1311A	8418	-0.16 (0.05)	0.001
Fasting serum HDL-cholesterol	*CD300LG*		17-39281652	T	C	3.5		Nonsyn	R82C	13063	-0.18 (0.03)	7.1×10^{-8}
Fasting serum HDL-cholesterol	*SLC26A2*		5-149340823	T	C	2.1		Nonsyn	R492W	13060	-0.18 (0.04)	1.5×10^{-5}
Fasting serum triacylglycerol	*PRRC2A*	rs41273264	6-31708085	A	C	4.2		Nonsyn	S1219Y	13326	0.13 (0.03)	5.2×10^{-5}

51 associations with metabolic phenotypes for 45 unique SNPs selected for replication in stage 3. More details on the selection process are given in ESM Methods section 4.1. Rs numbers are based on dbSNP 129. Chromosome and position based on genome build 36. All alleles are shown on the positive strand. HapMap allele frequencies are based on the HapMap CEU population release 27. All effects are shown for the minor allele. *P*-values have been corrected by genomic control.
