# Supplementary material for: Exome sequencing-driven discovery of coding polymorphisms associated with common metabolic phenotypes
Source: Diabetologia. 2012 Nov 19;56(2):298–310. doi: 10.1007/s00125-012-2756-1 (PMC3536959; doi:10.1007/s00125-012-2756-1)
Supplement: Supplementary file 27 — (PDF 233 kb) [file 125_2012_2756_MOESM27_ESM.pdf]

ESM Table 8 Association between 45 SNPs selected for replication and all 12 traits in Danish stage 2 data

SNP	Main association	Type 2 diabetes	Obesity	Hypertension	BMI	Waist circumference	Fasting plasma glucose	Fasting serum insulin	Fasting total cholesterol	Fasting serum HDL-cholesterol	Fasting serum triacylglycerol	Systolic BP	Diastolic BP
N		12179	10346	10590	14828	14547	9087	8419	13183	13063	13326	12651	12650
		OR / p	OR / p	OR / p	ß / p	ß / p	ß / p	ß / p	ß / p	ß / p	ß / p	ß / p	ß / p
rs4468717	BMI	1 / 0.53	1.2 / 0.0036	1.1 / 0.38	0.077 / 0.00064	0.06 / 0.0051	0.019 / 0.47	0.011 / 0.7	-0.0078 / 0.74	-0.08 / 3×10-4	0.069 / 0.0031	0.0074 / 0.75	0.019 / 0.39
rs35739383	BMI	0.96 / 0.17	0.93 / 0.012	0.99 / 0.83	-0.044 / 5×10-4	-0.038 / 0.0016	0.023 / 0.12	0.0048 / 0.76	-0.0074 / 0.57	0.025 / 0.043	-0.035 / 0.0074	-0.0035 / 0.78	-0.011 / 0.4
chr21_37003350	BMI	0.91 / 0.14	0.85 / 0.02	1.1 / 0.43	-0.095 / 0.00076	-0.076 / 0.0044	-0.023 / 0.48	-0.047 / 0.17	0.00058 / 0.98	0.048 / 0.079	-0.07 / 0.016	-0.0011 / 0.97	0.015 / 0.58
rs2147439	Obesity	1.1 / 0.3	1.5 / 0.00057	1.3 / 0.046	0.099 / 0.052	0.095 / 0.05	0.024 / 0.69	0.12 / 0.062	0.043 / 0.41	-0.061 / 0.22	0.04 / 0.44	0.098 / 0.059	0.095 / 0.061
chr10_132851444	Obesity	1.2 / 0.0084	1.4 / 0.00013	1.1 / 0.28	0.083 / 0.013	0.049 / 0.12	0.009 / 0.82	0.025 / 0.57	0.042 / 0.22	0.017 / 0.59	0.016 / 0.64	0.072 / 0.032	0.04 / 0.22
rs743581	Obesity	1.1 / 0.022	1.1 / 0.00079	1.1 / 0.022	0.028 / 0.027	0.02 / 0.097	0.0032 / 0.83	-0.0044 / 0.79	0.0057 / 0.67	-0.012 / 0.34	0.033 / 0.012	0.029 / 0.025	0.016 / 0.21
rs157581	Obesity	0.91 / 0.0046	0.89 / 0.00076	0.97 / 0.39	-0.039 / 0.0067	-0.037 / 0.0071	-0.0088 / 0.6	-0.037 / 0.039	0.052 / 0.00052	-0.029 / 0.04	0.075 / 3.5×10-7	-0.0056 / 0.7	-0.011 / 0.44
rs12720062	Obesity	0.72 / 0.0015	0.68 / 0.00038	0.84 / 0.094	-0.13 / 0.0039	-0.13 / 0.0013	0.045 / 0.35	-0.0022 / 0.97	-0.0093 / 0.84	0.021 / 0.62	-0.07 / 0.12	-0.043 / 0.33	-0.034 / 0.43
rs2425632	Obesity	0.91 / 0.15	0.78 / 0.00049	0.94 / 0.43	-0.096 / 0.0017	-0.069 / 0.017	-0.098 / 0.005	-0.094 / 0.013	-0.031 / 0.33	0.087 / 0.0037	-0.1 / 0.0013	-0.027 / 0.39	-0.052 / 0.089
chr5_14924560	Obesity	0.92 / 0.13	0.83 / 0.00073	0.95 / 0.38	-0.069 / 0.0026	-0.034 / 0.12	-0.023 / 0.38	-0.019 / 0.51	-0.016 / 0.51	0.017 / 0.46	-0.028 / 0.23	-0.0079 / 0.74	-0.013 / 0.57
rs11960429	Obesity	0.81 / 0.11	0.64 / 0.00098	0.87 / 0.33	-0.18 / 0.0014	-0.15 / 0.0062	0.027 / 0.66	-0.08 / 0.24	0.034 / 0.57	0.067 / 0.24	-0.037 / 0.53	-0.05 / 0.4	-0.03 / 0.6
chr6_41982766	Obesity	0.92 / 0.43	0.67 / 0.00085	0.86 / 0.2	-0.13 / 0.0062	-0.087 / 0.065	-0.013 / 0.82	-0.06 / 0.33	0.018 / 0.73	0.024 / 0.61	-0.023 / 0.65	-0.012 / 0.82	-0.0081 / 0.87
rs11553746	Obesity, BMI, WC	0.92 / 0.0056	0.89 / 9.4×10-5	0.93 / 0.031	-0.056 / 1.1×10-5	-0.047 / 0.00011	0.0012 / 0.93	0.015 / 0.36	-0.0027 / 0.84	0.026 / 0.034	-0.044 / 0.00076	-0.031 / 0.018	-0.024 / 0.057
rs2240953	Obesity, BMI, WC	1.2 / 0.047	1.3 / 0.00073	1.1 / 0.12	0.12 / 0.00017	0.12 / 4.1×10-5	0.042 / 0.25	0.041 / 0.31	0.018 / 0.57	-0.0011 / 0.97	0.024 / 0.45	0.047 / 0.14	0.034 / 0.27
chr17_39281652	HDL	1.1 / 0.24	1.1 / 0.38	0.99 / 0.95	0.032 / 0.34	0.038 / 0.23	0.028 / 0.48	0.041 / 0.33	-0.034 / 0.34	-0.18 / 7.2×10-8	0.12 / 0.00066	0.00013 / 1	0.0089 / 0.79
chr5_149340823	HDL	0.96 / 0.64	1 / 0.66	1 / 0.69	0.015 / 0.72	0.015 / 0.71	0.037 / 0.45	0.013 / 0.8	-0.012 / 0.79	-0.18 / 1.5×10-5	0.12 / 0.0073	0.035 / 0.43	-0.008 / 0.85
rs41265897	FINS	0.88 / 0.079	0.81 / 0.0047	0.78 / 0.00094	-0.078 / 0.013	-0.08 / 0.0074	-0.058 / 0.1	-0.14 / 0.00042	-0.08 / 0.015	-0.0039 / 0.9	-0.081 / 0.012	-0.09 / 0.0056	-0.09 / 0.0043
chr13_112385690	FINS	0.88 / 0.24	0.95 / 0.63	1 / 0.84	-0.057 / 0.24	-0.073 / 0.1	-0.1 / 0.06	-0.21 / 0.00042	0.034 / 0.49	0.074 / 0.12	-0.08 / 0.11	0.0057 / 0.91	-0.0089 / 0.85
rs12434581	FINS	1 / 0.99	1 / 0.67	1 / 0.51	-0.0025 / 0.84	-0.001 / 0.93	-0.0024 / 0.87	-0.064 / 5.3×10-5	-0.0057 / 0.66	0.0092 / 0.46	0.005 / 0.7	0.0052 / 0.69	0.0041 / 0.74
rs35118457	FINS	1 / 0.59	1 / 0.85	1 / 0.94	0.028 / 0.29	0.028 / 0.27	-0.0015 / 0.96	0.11 / 9×10-4	0.0025 / 0.93	-0.052 / 0.047	0.044 / 0.11	-0.00016 / 1	-0.017 / 0.53
chr19_8082890	FINS	0.9 / 0.25	0.82 / 0.035	0.94 / 0.49	-0.11 / 0.0058	-0.099 / 0.0078	-0.03 / 0.51	-0.16 / 0.001	-0.0077 / 0.85	0.1 / 0.0083	-0.12 / 0.0043	-0.0082 / 0.84	-0.019 / 0.62

SNP	Main association	Type 2 diabetes	Obesity	Hypertension	BMI	Waist circumference	Fasting plasma glucose	Fasting serum insulin	Fasting total cholesterol	Fasting serum HDL-cholesterol	Fasting serum triacylglycerol	Systolic BP	Diastolic BP
rs35605687	FINS	0.99 / 0.83	0.93 / 0.064	0.94 / 0.19	-0.041 / 0.02	-0.04 / 0.017	-0.014 / 0.5	-0.096 / 1.1×10-5	-0.02 / 0.28	0.011 / 0.53	-0.045 / 0.012	-0.0058 / 0.74	-0.015 / 0.38
rs41289373	FINS	0.86 / 0.21	0.93 / 0.52	1 / 0.75	-0.067 / 0.19	-0.11 / 0.027	-0.072 / 0.23	-0.25 / 9×10-5	0.074 / 0.16	0.075 / 0.13	-0.019 / 0.72	0.038 / 0.46	0.062 / 0.22
chr6_56588459	FINS	0.91 / 0.2	0.93 / 0.37	0.96 / 0.64	-0.03 / 0.37	-0.018 / 0.56	-0.017 / 0.66	-0.18 / 1.7×10-5	-0.025 / 0.47	0.0092 / 0.78	-0.046 / 0.18	0.0067 / 0.84	-0.013 / 0.69
rs41318029	FGLU	1.1 / 0.5	1.1 / 0.25	1 / 0.73	0.065 / 0.19	0.068 / 0.14	0.19 / 0.00086	0.092 / 0.14	-0.087 / 0.082	0.01 / 0.83	0.015 / 0.77	0.024 / 0.62	-0.024 / 0.61
rs1801319	FGLU	1 / 0.72	1 / 0.62	0.93 / 0.32	0.037 / 0.22	0.0038 / 0.9	0.13 / 0.00019	0.059 / 0.11	0.016 / 0.61	0.0034 / 0.91	0.035 / 0.26	-0.014 / 0.66	0.014 / 0.63
chr12_51840193	FGLU	0.99 / 0.94	0.67 / 0.0077	0.75 / 0.06	-0.15 / 0.013	-0.12 / 0.041	-0.24 / 0.00057	-0.052 / 0.49	-0.089 / 0.17	-0.019 / 0.76	-0.031 / 0.63	-0.074 / 0.23	-0.13 / 0.039
rs16986309	FGLU	0.98 / 0.66	0.96 / 0.45	0.96 / 0.45	-0.031 / 0.12	-0.03 / 0.12	-0.077 / 0.00087	-0.027 / 0.29	-0.047 / 0.023	-0.013 / 0.51	-0.041 / 0.047	-0.0064 / 0.75	-0.014 / 0.47
rs61750009	FGLU	0.91 / 0.2	0.99 / 0.91	0.92 / 0.28	0.0085 / 0.78	-0.0055 / 0.85	-0.12 / 0.00065	-0.024 / 0.53	-0.046 / 0.15	-0.054 / 0.071	0.02 / 0.53	-0.035 / 0.26	-0.04 / 0.19
rs1058065	FGLU	1.3 / 0.018	0.96 / 0.69	1.2 / 0.066	-0.0039 / 0.93	-0.0041 / 0.92	0.18 / 3×10-4	0.025 / 0.64	0.028 / 0.53	-0.11 / 0.012	0.073 / 0.1	0.042 / 0.34	0.11 / 0.01
rs34609592	FGLU	1 / 0.75	1.1 / 0.31	1 / 0.71	-0.02 / 0.68	-0.0067 / 0.88	0.19 / 0.00056	0.16 / 0.0086	-0.081 / 0.099	0.04 / 0.4	-0.07 / 0.15	0.004 / 0.93	-0.035 / 0.47
rs28633659	T2D	0.76 / 2×10-4	0.84 / 0.024	0.88 / 0.11	-0.06 / 0.057	-0.067 / 0.025	-0.06 / 0.093	-0.1 / 0.007	0.0063 / 0.85	0.02 / 0.51	-0.03 / 0.35	-0.05 / 0.12	-0.046 / 0.14
rs2296172	T2D	1.1 / 0.00065	1.1 / 0.011	1.1 / 0.14	0.035 / 0.016	0.037 / 0.0074	0.042 / 0.012	0.021 / 0.25	-0.025 / 0.096	-0.049 / 0.00061	0.018 / 0.24	0.013 / 0.39	0.019 / 0.18
chr16_25147310	T2D	0.71 / 0.00038	0.8 / 0.026	0.94 / 0.51	-0.098 / 0.016	-0.1 / 0.0093	-0.024 / 0.6	-0.015 / 0.75	-0.071 / 0.09	0.052 / 0.19	-0.094 / 0.023	-0.045 / 0.27	-0.047 / 0.23
rs16985907	T2D	1.3 / 7.5×10-5	1.2 / 0.008	1.3 / 0.0034	0.074 / 0.017	0.085 / 0.0042	0.014 / 0.72	0.043 / 0.28	0.041 / 0.2	-0.051 / 0.1	0.09 / 0.0055	0.083 / 0.0098	0.037 / 0.24
rs7607980	T2D	0.8 / 3×10-7	0.92 / 0.076	0.91 / 0.052	-0.019 / 0.3	-0.025 / 0.16	0.03 / 0.15	-0.022 / 0.33	-0.00071 / 0.97	0.072 / 6.8×10-5	-0.05 / 0.0091	-0.026 / 0.17	0.0031 / 0.87
rs9856575	T2D	1.4 / 0.00055	1.2 / 0.17	1.3 / 0.043	0.09 / 0.04	0.07 / 0.093	0.062 / 0.25	-0.094 / 0.1	0.054 / 0.23	0.077 / 0.072	-0.01 / 0.82	0.11 / 0.013	0.071 / 0.1
chr5_133929838	T2D	0.66 / 0.00089	0.81 / 0.1	0.86 / 0.25	-0.11 / 0.031	-0.12 / 0.017	-0.1 / 0.064	-0.033 / 0.59	-0.00088 / 0.99	0.018 / 0.73	-0.062 / 0.25	-0.03 / 0.57	0.0098 / 0.85
rs34882957	T2D	0.58 / 0.00043	0.68 / 0.011	0.65 / 0.005	-0.18 / 0.0037	-0.15 / 0.012	-0.084 / 0.2	0.021 / 0.77	0.042 / 0.51	0.038 / 0.54	-0.06 / 0.34	-0.1 / 0.1	-0.082 / 0.18
chr7_23272666	T2D	1.4 / 0.00055	1.1 / 0.17	1.3 / 0.02	0.049 / 0.25	0.034 / 0.41	0.046 / 0.37	0.026 / 0.64	-0.012 / 0.79	-0.034 / 0.41	0.054 / 0.22	0.13 / 0.0025	0.097 / 0.021
rs56204700	T2D	1.4 / 0.00076	1.1 / 0.29	1.2 / 0.17	0.056 / 0.19	0.03 / 0.46	0.031 / 0.55	0.018 / 0.74	0.081 / 0.069	-0.028 / 0.51	0.063 / 0.16	0.069 / 0.11	0.048 / 0.25
rs60980157	T2D	0.89 / 0.00028	0.93 / 0.043	1 / 0.7	-0.017 / 0.23	-0.022 / 0.11	-0.026 / 0.11	0.011 / 0.54	0.0099 / 0.51	-0.001 / 0.94	-0.011 / 0.45	0.0022 / 0.88	0.02 / 0.17
rs36103207	T2D, WC, FGLU	1.3 / 0.00083	1.3 / 0.0018	1.2 / 0.048	0.1 / 0.0018	0.1 / 0.00083	0.13 / 0.00055	0.013 / 0.75	-0.026 / 0.44	-0.084 / 0.0082	0.061 / 0.065	0.073 / 0.028	0.03 / 0.36
rs41273264	TRIG	1.2 / 0.0084	1.1 / 0.32	1 / 0.7	0.043 / 0.16	0.037 / 0.21	0.029 / 0.43	0.063 / 0.11	0.038 / 0.23	-0.077 / 0.011	0.13 / 5.2×10-5	0.015 / 0.64	0.0023 / 0.94
rs41273513	WC	0.76 / 0.024	0.7 / 0.0047	0.92 / 0.5	-0.14 / 0.0075	-0.17 / 0.00045	0.038 / 0.5	0.032 / 0.61	-0.061 / 0.25	0.038 / 0.45	-0.083 / 0.11	-0.069 / 0.2	-0.053 / 0.31

Association results for 45 selected SNPs for all 12 traits analysed in Danish data in stage 2. Data are OR / p-value or beta / p-value. The trait for which each SNP is selected for stage 3 is shown in the Main association column. T2D, type 2 diabetes; WC, waist circumference; FGLU, fasting plasma glucose; FINS, fasting serum insulin; TRIG, fasting triacylglycerol; HDL, high-density lipoprotein cholesterol; BMI, body mass index.
