# Supplementary material for: Exome sequencing-driven discovery of coding polymorphisms associated with common metabolic phenotypes
Source: Diabetologia. 2012 Nov 19;56(2):298–310. doi: 10.1007/s00125-012-2756-1 (PMC3536959; doi:10.1007/s00125-012-2756-1)
Supplement: Supplementary file 28 — (PDF 348 kb) [file 125_2012_2756_MOESM28_ESM.pdf]

ESM Table 9 Discovery and replication studies of all 51 associations for 45 SNPs selected for replication

Basic information							Discovery				Replication				Heterogeneity		Combined		
Trait	rs (dbSNP 129)	Chr-Position	Gene	Annotation		Effect / Other	EAF	*n*	Estimate	*P*	EAF	*n*	Estimate	*P*	I^2	*P*	*n*	Estimate	*P*
Type 2 diabetes	rs7607980	2-165259447	*COBLL1*	Mis	N939D	C/T	12.5	12177	0.80 (0.74-0.87)	3 × 10-7	10.8	36407	0.88 (0.84-0.93)	5.4 × 10-6	0	0.69	48584	0.86 (0.82-0.9)	1.2×10-11
Type 2 diabetes	rs2296172	1-39608404	*MACF1*	Mis	M2290V	G/A	23.4	12175	1.12 (1.05-1.19)	0.00065	21.7	63896	1.1 (1.06-1.14)	5.8 × 10-7	48	0.017	76071	1.1 (1.07-1.13)	8.2×10-10
Type 2 diabetes	rs60980157	9-138355236	*GPSM1*	Mis	S368L	T/C	24.6	12175	0.89 (0.84-0.94)	0.00028	23.6	33815	0.92 (0.87-0.98)	0.0049	-59	0.28	45990	0.91 (0.87-0.94)	2.8×10-6
Type 2 diabetes	rs34882957	5-39367651	*C9*	Mis	P167S	A/G	1	12177	0.58 (0.44-0.77)	0.00043	0.7	31592	0.92 (0.74-1.15)	0.46	0	0.58	43769	0.78 (0.65-0.92)	0.0039
Type 2 diabetes	rs56204700	7-97659791	*LMTK2*	Mis	I693T	C/T	2.2	12179	1.38 (1.15-1.65)	0.00076	4.6	32689	1.02 (0.93-1.11)	0.62	6.8	0.17	44868	1.08 (1-1.17)	0.044
Type 2 diabetes	none	7-23272666	*GPNMB*	Mis	A354T	A/G	2.2	12179	1.39 (1.17-1.66)	0.00055	2.1	32733	1.04 (0.92-1.18)	0.56	0	0.93	44912	1.14 (1.03-1.26)	0.01
Type 2 diabetes	none	5-133929838	*PHF15*	Mis	G368V	T/G	1.5	12151	0.66 (0.52-0.83)	0.00089	2	45271	0.99 (0.89-1.1)	0.88	42	0.033	57422	0.93 (0.84-1.02)	0.12
Type 2 diabetes	rs28633659	1-1844406	*C1orf222*	Mis	E187K	T/C	3.9	12179	0.76 (0.66-0.87)	2 × 10-4	4.3	57180	1 (0.93-1.07)	0.97	0	0.45	69359	0.95 (0.89-1.01)	0.077
Type 2 diabetes	rs36103207	10-103978255	*ELOVL3*	Mis	D109N	A/G	3.7	12178	1.28 (1.12-1.47)	0.00083	4	58789	0.99 (0.91-1.08)	0.8	-6.4	0.25	70967	1.06 (0.99-1.14)	0.098
Type 2 diabetes	rs16985907	19-59938624	*KIR3DL3*	Mis	A348P	C/G	4.1	12179	1.31 (1.15-1.49)	7.5 × 10-5	4.5	32190	0.94 (0.87-1.03)	0.2	0	0.79	44369	1.05 (0.98-1.13)	0.2
Type 2 diabetes	none	16-25147310	*AQP8*	Mis	R261Q	A/G	2.4	12175	0.71 (0.59-0.85)	0.00038	2.3	31597	1.13 (1-1.27)	0.056	45	0.027	43772	0.98 (0.88-1.08)	0.61
Type 2 diabetes	rs9856575	3-52485632	*NISCH*	Mis	I299V	G/A	2.1	12177	1.4 (1.17-1.68)	0.00055	1.8	32675	0.91 (0.8-1.03)	0.15	0	0.67	44852	1.05 (0.94-1.17)	0.33
Obesity	rs11553746	2-262203	*ACP1*	Mis	T95I	T/C	37.8	10324	0.89 (0.84-0.94)	9.4 × 10-5	36.2	23744	0.95 (0.91-0.98)	0.0051	0	0.68	34068	0.93 (0.9-0.96)	3.5×10-6
Obesity	rs743581	15-72115194	*PML*	Mis	G780V	T/G	37.1	10346	1.11 (1.05-1.18)	0.00079	37.3	16628	1.04 (0.99-1.09)	0.1	0	0.98	26974	1.07 (1.03-1.11)	0.00045
Obesity	none	6-41982766	*MED20*	3'UTR	NA	A/C	1.6	10345	0.67 (0.54-0.84)	0.00085	1.4	19794	0.89 (0.75-1.07)	0.21	0	0.54	30139	0.8 (0.7-0.92)	0.0015
Obesity	rs2147439	10-11951710	*C10orf47*	Mis	E203K	A/G	1.5	10346	1.53 (1.22-1.93)	0.00057	1.1	25187	1.07 (0.89-1.29)	0.46	0	0.74	35533	1.24 (1.07-1.43)	0.0041
Obesity	none	10-132851444	*TCERG1L*	Mis	G322E	T/C	3.7	10346	1.37 (1.18-1.59)	0.00012	2.3	20766	1.04 (0.89-1.21)	0.6	23	0.1	31112	1.2 (1.08-1.34)	0.0011
Obesity	rs2425632	20-42325348	*GDAP1L1*	Syn	NA	T/C	4.2	10346	0.78 (0.68-0.89)	0.00049	4	20881	0.98 (0.89-1.09)	0.74	0	0.9	31227	0.9 (0.84-0.98)	0.014

Basic information							Discovery				Replication				Heterogeneity		Combined		
Trait	rs (dbSNP 129)	Chr-Position	Gene	Annotation		Effect / Other	EAF	*n*	Estimate	*P*	EAF	*n*	Estimate	*P*	I^2	*P*	*n*	Estimate	*P*
Obesity	rs11960429	5-180099283	*OR2Y1*	Mis	R128C	A/G	1.2	10346	0.64 (0.5-0.82)	0.00098	1.4	20027	1 (0.84-1.18)	0.95	0	0.55	30373	0.87 (0.75-1)	0.043
Obesity	rs12720062	2-220148160	*INHA*	Mis	A257T	A/G	2.1	10306	0.68 (0.56-0.83)	0.00038	1.8	16543	1 (0.85-1.18)	0.99	0.1	0.17	26849	0.85 (0.75-0.97)	0.016
Obesity	rs157581	19-50087554	*TOMM40*	Syn	NA	C/T	24.2	10344	0.89 (0.83-0.95)	0.00076	23	16635	1.01 (0.96-1.06)	0.78	0	0.44	26979	0.96 (0.92-1)	0.042
Obesity	rs2240953	9-130150713	*SLC27A4*	Mis	G209S	A/G	4.2	10308	1.29 (1.12-1.48)	0.00073	4.5	21255	0.97 (0.88-1.07)	0.55	0	0.75	31563	1.07 (0.99-1.16)	0.11
Obesity	none	5-14924560	*ANKH*	5'UTR	NA	T/C	7.7	10346	0.83 (0.75-0.92)	0.00073	8	16050	1 (0.92-1.07)	0.91	26	0.08	26396	0.94 (0.88-0.99)	0.027
BMI	rs11553746	2-262203	*ACP1*	Mis	T95I	T/C	37.8	14793	-0.056 (0.012)	1.1 × 10-5	36.5	39931	-0.018 (0.0073)	0.014	0	0.96	54724	0.0062 (0.0062)	1.2×10-5
BMI	rs4468717	18-3447606	*TGIF1*	Mis	P163S	T/C	7.2	14759	0.077 (0.021)	0.00064	7.7	9217	0.038 (0.027)	0.19	20	0.052	23976	0.017 (0.017)	0.00047
BMI	none	21-37003350	*SIM2*	Mis	A63V	T/C	5.1	14827	-0.095 (0.026)	0.00076	2.8	23195	-0.022 (0.028)	0.61	-77	0.32	38022	0.019 (0.019)	0.012
BMI	rs35739383	18-8773835	*CCDC165*	Mis	M242T	T/C	39.6	14820	-0.044 (0.012)	5 × 10-4	39.7	21596	-0.0012 (0.012)	0.94	-28	0.21	36416	0.0085 (0.0085)	0.023
BMI	rs2240953	9-130150713	*SLC27A4*	Mis	G209S	A/G	4.2	14776	0.12 (0.029)	0.00017	4.6	36610	-0.0048 (0.02)	0.92	0	0.66	51386	0.016 (0.016)	0.054
Waist circumference	rs11553746	2-262203	*ACP1*	Mis	T95I	T/C	37.8	14513	-0.046 (0.011)	0.00011	36	29044	-0.0043 (0.0083)	0.5	0	0.96	43557	0.0066 (0.0066)	0.0052
Waist circumference	rs41273513	1-156843597	*OR10Z1*	Mis	I249V	G/A	1.5	14544	-0.17 (0.045)	0.00045	3	24134	0.0018 (0.026)	0.71	-13	0.26	38678	0.022 (0.022)	0.015
Waist circumference	rs36103207	10-103978255	*ELOVL3*	Mis	D109N	A/G	3.7	14545	0.1 (0.028)	0.00083	4.1	37186	0.003 (0.019)	0.68	0	0.62	51731	0.016 (0.016)	0.034
Waist circumference	rs2240953	9-130150713	*SLC27A4*	Mis	G209S	A/G	4.2	14496	0.12 (0.027)	4.1 × 10-5	4.8	29202	-0.02 (0.02)	0.3	0	0.99	43698	0.016 (0.016)	0.13
Fasting plasma glucose	rs61750009	2-219780675	*ZFAND2B*	Mis	P71H	A/C	4.2	9080	-0.12 (0.035)	0.00065	2.8	25560	-0.044 (0.025)	0.12	29	0.081	34640	0.02 (0.02)	0.0021
Fasting plasma glucose	none	12-51840193	*CSAD*	Mis	D382N	T/C	1	9087	-0.24 (0.069)	0.00057	1.7	24270	-0.02 (0.032)	0.27	20	0.12	33357	0.029 (0.029)	0.0062
Fasting plasma glucose	rs16986309	19-60401886	*PTPRH*	Mis	L543F	A/G	10.9	9087	-0.077 (0.023)	0.00087	11.5	23231	-0.0098 (0.013)	0.44	0	0.93	32318	0.012 (0.012)	0.016
Fasting plasma glucose	rs36103207	10-103978255	*ELOVL3*	Mis	D109N	A/G	3.7	9086	0.13 (0.039)	0.00055	3.7	23254	0.016 (0.023)	0.61	-50	0.4	32340	0.02 (0.02)	0.024
Fasting plasma glucose	rs1058065	2-27441228	*EIF2B4*	Syn	NA	A/G	2.1	9087	0.18 (0.049)	3 × 10-4	1.9	21950	9e-04 (0.033)	0.96	-5.3	0.22	31037	0.028 (0.028)	0.046
Fasting plasma glucose	rs41318029	10-101580476	*ABCC2*	Mis	G921S	A/G	1.6	9087	0.19 (0.056)	0.00086	0.8	23319	0.0092 (0.049)	0.98	0	0.85	32406	0.037 (0.037)	0.081

Basic information							Discovery				Replication				Heterogeneity		Combined		
Trait	rs (dbSNP 129)	Chr-Position	Gene	Annotation		Effect / Other	EAF	*n*	Estimate	*P*	EAF	*n*	Estimate	*P*	I^2	*P*	*n*	Estimate	*P*
Fasting plasma glucose	rs34609592	9-135905516	*BRD3*	Mis	A172V	A/G	1.7	9087	0.19 (0.055)	0.00056	2.4	22049	-0.01 (0.028)	0.88	0	0.62	31136	0.025 (0.025)	0.083
Fasting plasma glucose	rs1801319	11-104330859	*CASP4*	5'UTR	NA	T/C	4.3	9087	0.13 (0.034)	0.00019	2.9	18034	-0.04 (0.029)	0.17	0	0.9	27121	0.022 (0.022)	0.3
Fasting serum insulin	rs35605687	4-2985351	*GRK4*	Mis	V215I	A/G	14.6	8417	-0.096 (0.022)	1.1 × 10-5	20.3	17256	-0.028 (0.014)	0.059	9.6	0.11	25673	0.012 (0.012)	4.8×10-5
Fasting serum insulin	none	6-56588459	*DST*	Mis	I2589V	C/T	3.6	8418	-0.18 (0.042)	1.7 × 10-5	2.2	12220	-0.06 (0.043)	0.2	0	0.6	20638	0.03 (0.03)	0.00019
Fasting serum insulin	rs12434581	14-73524572	*ENTPD5*	5'UTR	NA	A/T	40.8	8413	-0.064 (0.016)	5.3 × 10-5	42.2	5135	-0.012 (0.02)	0.55	0	1	13548	0.012 (0.012)	0.00038
Fasting serum insulin	rs41265897	1-33608751	*PHC2*	Mis	A145V	A/G	4	8417	-0.14 (0.039)	0.00042	4.6	18477	-0.016 (0.025)	0.55	0	0.37	26894	0.021 (0.021)	0.014
Fasting serum insulin	none	19-8082890	*FBN3*	Mis	G1311A	G/C	2.6	8418	-0.16 (0.049)	0.001	1.2	18410	-0.038 (0.048)	0.46	0	0.56	26828	0.034 (0.034)	0.014
Fasting serum insulin	none	13-112385690	*C13orf35*	Mis	R121W	T/C	1.7	8419	-0.21 (0.059)	0.00042	4.2	18556	-0.01 (0.026)	0.57	-0.5	0.14	26975	0.024 (0.024)	0.014
Fasting serum insulin	rs35118457	17-37516926	*DHX58*	Mis	R95Q	T/C	5.8	8418	0.11 (0.033)	9 × 10-4	4.4	17195	0.0085 (0.026)	0.76	-27	0.21	25613	0.021 (0.021)	0.032
Fasting serum insulin	rs41289373	6-151714763	*AKAP12*	Mis	A1182T	A/G	1.3	8381	-0.26 (0.065)	9 × 10-5	1.1	18547	0.039 (0.048)	0.44	0	0.52	26928	0.039 (0.039)	0.12
Fasting plasma HDL-cholesterol	none	17-39281652	*CD300LG*	Mis	R82C	T/C	3.5	13063	-0.18 (0.032)	7.2 × 10-8	3.1	20822	-0.14 (0.027)	1.5 × 10-7	0	0.85	33885	0.02 (0.02)	8.5×10-14
Fasting plasma HDL-cholesterol	none	5-149340823	*SLC26A2*	Mis	R492W	T/C	2.1	13060	-0.18 (0.039)	1.5 × 10-5	2.2	18804	0.0063 (0.033)	0.75	2.6	0.19	31864	0.025 (0.025)	0.011
Fasting plasma triacylglycerol	rs41273264	6-31708085	*PRRC2A*	Mis	S1219Y	A/C	4.2	13326	0.13 (0.03)	5.2 × 10-5	2.2	16135	0.1 (0.037)	0.0093	-22	0.29	29461	0.023 (0.023)	3.4×10-6

Estimates are OR (95% CI) for binary variables (type 2 diabetes and obesity) or beta (standard error) on a rank normalized scale for quantitative traits (Fasting plasma HDL-cholesterol, fasting plasma triacylglycerol, fasting plasma glucose and BMI). Reported estimates are based on replication (stage 3) data. Estimates of effects and *P*-values for binary traits in replication and combined meta-analyses were calculated based on effect size and standard error, where effect size was weighted according to the estimated standard errors by using the inverse corresponding standard error. For quantitative traits an overall z-statistics was calculated relative to each reference allele estimated based on *P*-value and direction of effect adjusted for the number of individuals in each sample. Heterogeneity was calculated including only the replication samples. Alleles are given on the positive strand. rs-numbers are from dbSNP 129. Chromosome and position for SNPs are stated according to Build 36.3 (hg18). Chr: chromosome; EAF: Effect allele frequency.
