# Supplementary material for: Exome sequencing-driven discovery of coding polymorphisms associated with common metabolic phenotypes
Source: Diabetologia. 2012 Nov 19;56(2):298–310. doi: 10.1007/s00125-012-2756-1 (PMC3536959; doi:10.1007/s00125-012-2756-1)
Supplement: Supplementary file 29 — (PDF 290 kb) [file 125_2012_2756_MOESM29_ESM.pdf]

ESM Table 10 In silico replication in existing GWAS meta-analysis databases for eight associations with nominal replication in stage 3 data.

Trait	rs (dbSNP 129)	Chr-Position (build 36)	Gene	EAF (%)	Proxy (r^2)	N	Effect/P	Consistent direction	Reason for missing results
Fasting serum HDL-cholesterol	None	17-39281652	*CD300LG*	3.5					No proxy
Type 2 diabetes	rs7607980	2-165259447	*COBLL1*	12.5		22570	0.93 (0.87-0.99), *P*=0.016	Y	
Type 2 diabetes	rs2296172	1-39608404	*MACF1*	23.4		22570	1.06 (1.01-1.11), *P*=0.016	Y	
Type 2 diabetes	rs60980157	9-138355236	*GPSM1*	24.6					No proxy
Fasting serum triacylglycerol	rs41273264	6-31708085	*PRRC2A*	4.2					No proxy
Obesity	rs11553746	2-262203	*ACP1*	37.8					No data for this trait
BMI	rs11553746	2-262203	*ACP1*	37.8	rs3791221 (1)	123842	P=0.093	Y	

Due to a minor overlap in samples between stage 3 and the GWAS meta-analysis data these data were not meta-analysed with stage 2 and stage 3 data and consequently they represent semi-independent replication. Data for type 2 diabetes were contributed by the DIAGRAM Consortium [27], data on BMI by GIANT Consortium [28] (http://www.broadinstitute.org/collaboration/giant/index.php/GIANT_consortium) and data on glycaemic traits were contributed by MAGIC investigators [29] and were downloaded from www.magicinvestigators.org. For two SNPs the lead SNPs were in the GWAS meta-analyses data while *in silico* replication for two SNPs was based on proxies. Here LD was estimated from data from the 1000 Genomes Project. Three SNPs had no good proxy ($r^2 > 0.4$). For one trait no GWAS data could be investigated.
