# Supplementary material for: Exome sequencing-driven discovery of coding polymorphisms associated with common metabolic phenotypes
Source: Diabetologia. 2012 Nov 19;56(2):298–310. doi: 10.1007/s00125-012-2756-1 (PMC3536959; doi:10.1007/s00125-012-2756-1)
Supplement: Supplementary file 30 — (PDF 296 kb) [file 125_2012_2756_MOESM30_ESM.pdf]

ESM Table 11 Pleiotropic effects of SNPs associated with a metabolic phenotype in the main analysis

	chr17_39281652	rs7607980	rs2296172
Locus	*CD300LG*	*COBLL1*	*MACF1*
Lead Trait	HDL-cholesterol	Type 2 diabetes	Type 2 diabetes
	n / β / *p*	*n* / β / *p*	*n* / β / *p*
Type 2 diabetes	50167 / 1 / 0.44	48584 / 0.88 / 1.2e-11	76071 / 1.1 / 8.2e-10
Obesity	34203 / 0.97 / 0.97	33109 / 1.1 / 0.29	43166 / 1 / 0.021
BMI (kg/m2)	54931 / -0.02 / 0.75	52981 / 0.045 / 0.0069	70764 / 0.0016 / 0.19
Waist-to-hip ratio	10836 / 0.02 / 0.48	10834 / -0.038 / 0.016	10831 / 0.014 / 0.28
Waist circumference (cm)	43744 / -0.034 / 0.73	42050 / 0.02 / 0.83	56677 / 0.0079 / 0.054
Height (cm)	15558 / 0.06 / 0.016	15557 / 0.0073 / 0.59	15554 / 0.016 / 0.13
Fasting serum insulin (pM)	26908 / 0.034 / 0.14	25461 / -0.029 / 0.057	26831 / 0.003 / 0.4
30 min. serum insulin (pM)	5277 / 0.0093 / 0.86	5275 / -0.0074 / 0.8	5274 / 0.04 / 0.091
120 min. serum insulin (pM)	5330 / 0.035 / 0.5	5328 / -0.031 / 0.28	5327 / 0.032 / 0.17
Fasting plasma glucose (mM)	34572 / -0.013 / 0.91	33094 / 0.013 / 0.14	34452 / 0.016 / 0.0072
30 min. plasma glucose (mM)	5472 / 0.086 / 0.085	5470 / 0.035 / 0.19	5469 / 0.031 / 0.16
120 min. plasma glucose (mM)	5761 / 0.029 / 0.57	5759 / -0.044 / 0.11	5758 / 0.043 / 0.055
ISI Matsuda	5813 / -0.061 / 0.23	5811 / 0.014 / 0.61	5811 / -0.057 / 0.011
HOMA-IR (mU/L×mM)	8414 / 0.038 / 0.37	8412 / -0.016 / 0.48	8412 / 0.029 / 0.11
BIGTT-SI	4906 / -0.046 / 0.38	4904 / 0.018 / 0.53	4904 / -0.043 / 0.062
Insulinogenic index	5089 / -0.0086 / 0.87	5087 / -0.0062 / 0.83	5087 / 0.023 / 0.33
BIGTT-AIR	4897 / -0.035 / 0.52	4895 / -0.044 / 0.14	4895 / 0.011 / 0.64
HbA1C (%)	12377 / 0.024 / 0.51	12375 / -0.046 / 0.021	12374 / 0.029 / 0.065
Fasting serum triacylglycerol (mmol/l)	35765 / 0.12 / 1.8e-08	34624 / -0.0053 / 0.057	35674 / 0.0086 / 0.2
Fasting serum cholesterol (mmol/l)	13183 / -0.034 / 0.34	13181 / -0.00071 / 0.97	13179 / -0.025 / 0.096
Fasting serum HDL-cholesterol (mmol/l)	33885 / -0.14 / 8.5e-14	32758 / 0.0033 / 0.0056	33811 / -0.037 / 3.6e-06
Systolic blod pressure (mmHg)	12651 / 0.00013 / 1	12649 / -0.026 / 0.17	12648 / 0.013 / 0.39
Diatolic blod pressure (mmHg)	12650 / 0.0089 / 0.79	12648 / 0.0031 / 0.87	12647 / 0.019 / 0.18
Leptin (unit)	5396 / 0.038 / 0.39	5394 / 0.038 / 0.11	5393 / -0.0046 / 0.81
Adiponectin (unit)	5483 / -0.045 / 0.37	5481 / 0.059 / 0.029	5480 / -0.029 / 0.19
hs-CRP (unit)	5233 / 0.066 / 0.22	5231 / -0.0098 / 0.74	5230 / 0.024 / 0.31

Data are *N* / effect / *P*-value. Effects of quantitative traits are on a rank normalized scale while effects for binary traits (type 2 diabetes and obesity) are odds ratio. Association analysis for each single SNP was performed in largest possible sample size available. Study samples included are all samples described in ESM Methods section 0. Lead trait describes the metabolic phenotype for which main association is found (Table 2). Statistical analyses within each study sample were done as described in section 3.3. Meta-analysis of several study samples were done as described in section 4.2. Derived indices of insulin secretion are Insulinogenic Index (calculated as: serum insulin$[pM]_{30\ min}$ – serum insulin$[pM]_{0min}$)/plasma glucose$[mM]_{30\ min}$) and BIGTT-acute insulin response (AIR) [30]. Derived indices of insulin resistance are homeostasis model assessment of insulin resistance (HOMA-IR) [31], Insulin Sensitivity Index (ISI) Matsuda [32] and BIGTT-sensitivity index (SI) [30]. Hs-CRP: high sensitivity C-reactive protein
